# Elucidating the pharmacological mechanism by which Si-Wu-Tang induces cellular senescence in breast cancer via multilevel data integration

**DOI:** 10.18632/aging.204185

**Published:** 2022-07-19

**Authors:** Minhong Zhao, Botao Pan, Yanjun He, Bo Niu, Xiuan Gao

**Affiliations:** 1 Affiliated Foshan Maternity and Child Healthcare Hospital, Southern Medical University, Foshan 528000, PR China; 2 Department of Emergency, Affiliated Foshan Maternity and Child Healthcare Hospital, Southern Medical University, Foshan 528000, PR China

**Keywords:** Si-Wu-Tang (SWT), cellular senescence, breast cancer, aging/senescence-induced genes, senescence-associated secretory phenotype (SASP)

## Abstract

Traditional Chinese medicine (TCM) is a promising strategy for effectively treating cancer by inducing cellular senescence with minimal side effects. Si-Wu-Tang (SWT) is a TCM composed of four herbs that is commonly used in China for the treatment of gynecological diseases; SWT can prevent breast cancer (BC), but the molecular mechanism by which SWT induces cellular senescence and its clinical application value remain unknown. We identified 335 differentially expressed genes (DEGs) in SWT-treated MCF-7 cells through Gene Expression Omnibus (GEO) dataset analysis. Gene Ontology (GO) and Kyoto Encyclopedia of Genes and Genomes (KEGG) analyses revealed the enrichment of biological processes and key signaling pathways including cellular senescence, the cell cycle, the MAPK signaling pathway, and the p53 signaling pathway. Additionally, SWT induced BC cell senescence by upregulating the expression of 33 aging/senescence-induced genes (ASIGs). According to LASSO regression analysis, NDRG1, ERRFI1, SOCS1, IRS2, IGFBP4, and BIRC3 levels were associated with BC prognosis and were used to develop risk scores. ERRFI1, SOCS1, IRS2, IGFBP4, and BIRC3 were identified as protective factors (*P* < 0.05, HR < 1), while NDRG1 was identified as a risk factor (*P* < 0.05, HR > 1). Notably, patients with low risk scores had increased senescence-associated secretory phenotypes (SASPs) and immune cell infiltration. Overall, we systematically integrated biological databases and biocomputational methods to reveal the mechanisms by which SWT induces senescence in breast cancer and its clinical value.

## INTRODUCTION

According to the International Agency for Research on Cancer, female breast cancer (BC) has surpassed lung cancer as the most frequently diagnosed cancer, and an estimated 2,261,419 new cases and 684,996 related deaths were recorded worldwide in 2020 [1]. Although the survival rates of BC patients have improved with early screening and standard of care, BC mortality rates remain high. The 5-year overall survival (OS) rate of patients with metastasis-free breast cancer is higher than 80%, but the 5-year survival rate of patients with metastatic BC at the time of diagnosis decreases sharply to approximately 10–33% [2]. BC remains a primary cause of cancer-related death in females.

Depending on the clinical tumor subtype, the mainstay of BC treatment includes endocrine therapy, anti-HER2-targeted therapy, radiotherapy, and chemotherapy. While these therapeutic approaches have prolonged patient survival, substantial issues, including relapse after an objective response to chemotherapy, drug-induced side effects, and drug resistance, remain unresolved; these issues ultimately lead to relapse or the development of advanced primary and metastatic tumors [3–5]. In recent years, a promising strategy, namely, the regulation of cellular senescence, has emerged; cellular senescence permanently inhibits the proliferative capacity of cells, inducing irreversible cell cycle arrest. Senescent cells are characterized by morphological and metabolic changes, chromatin remodeling, changes in gene expression, and the appearance of a proinflammatory phenotype known as the senescence-associated secretory phenotype (SASP) [6].

Traditional Chinese medicine (TCM) has been used in clinics in Asia for thousands of years. Many modern pharmacological studies have shown that TCM alone or as an adjunct to conventional chemotherapy is effective in the clinical treatment of cancer, including breast cancer [7, 8]. In recent years, many studies have shown that TCM induces cell senescence by inhibiting telomerase activity, inducing DNA damage, inducing SASP development, and activating or inactivating oncogene expression, thereby inhibiting the occurrence and development of tumors [9]. Compared with other antitumor strategies, the process of senescence induced by TCM is relatively slow and has the strong advantage that it does not cause extensive damage to surrounding tissues or skin. Due to its remarkable efficiency and minimal side effects, TCM is considered a promising strategy for treating cancer via the induction of cellular senescence.

Si-Wu-Tang (SWT), which consists of four herbs, namely, *Radix Paeoniae Alba* (Bai Shao), *Radix Angelicae sinensis* (Dang Gui), *Rhizoma Chuanxiong* (Chuan Xiong), and *Radix Rehmanniae Preparata* (Shu Di Huang), has been widely used in East Asia as a traditional formulation for treating gynecological diseases for over 1000 years [10]. In the clinic, SWT is often used to relieve menstrual discomfort, peri- or postmenopausal syndromes, climacteric syndrome, and other estrogen-related diseases. A study by Zhining Wen et al. [11] showed that the characteristics of SWT-treated MCF-7 cells closely matched those of estradiol (E2)-treated MCF-7 cells, which is consistent with the use of SWT to treat diseases that arise specifically in women, and revealed the estrogen-like effects of SWT. Other modern pharmacological studies have shown that SWT exerts a regulatory effect on the proliferation of BC cells by regulating HER-2, PI3K/AKT, and MAPK signaling [12]. However, few studies have explored the molecular mechanism by which SWT affects breast cancer from the perspective of cellular senescence induction.

Due to the complexity of the components and targets of TCM and the pathological mechanism of breast cancer, it is difficult to efficiently and comprehensively explore the molecular mechanism underlying the antitumor effects of TCM in a single experiment, and this difficulty increases the gap in research on Chinese and Western medicine. With the rapid development of systems biology and integrative pharmacology techniques, the emergence of network pharmacology has provided opportunities for breakthroughs in TCM research [13]. This method has been successfully used to elucidate the multitarget effects of TCM in various diseases, effectively bridging the gap between research on Western and Chinese medicine [14, 15].

**Figure 1:**
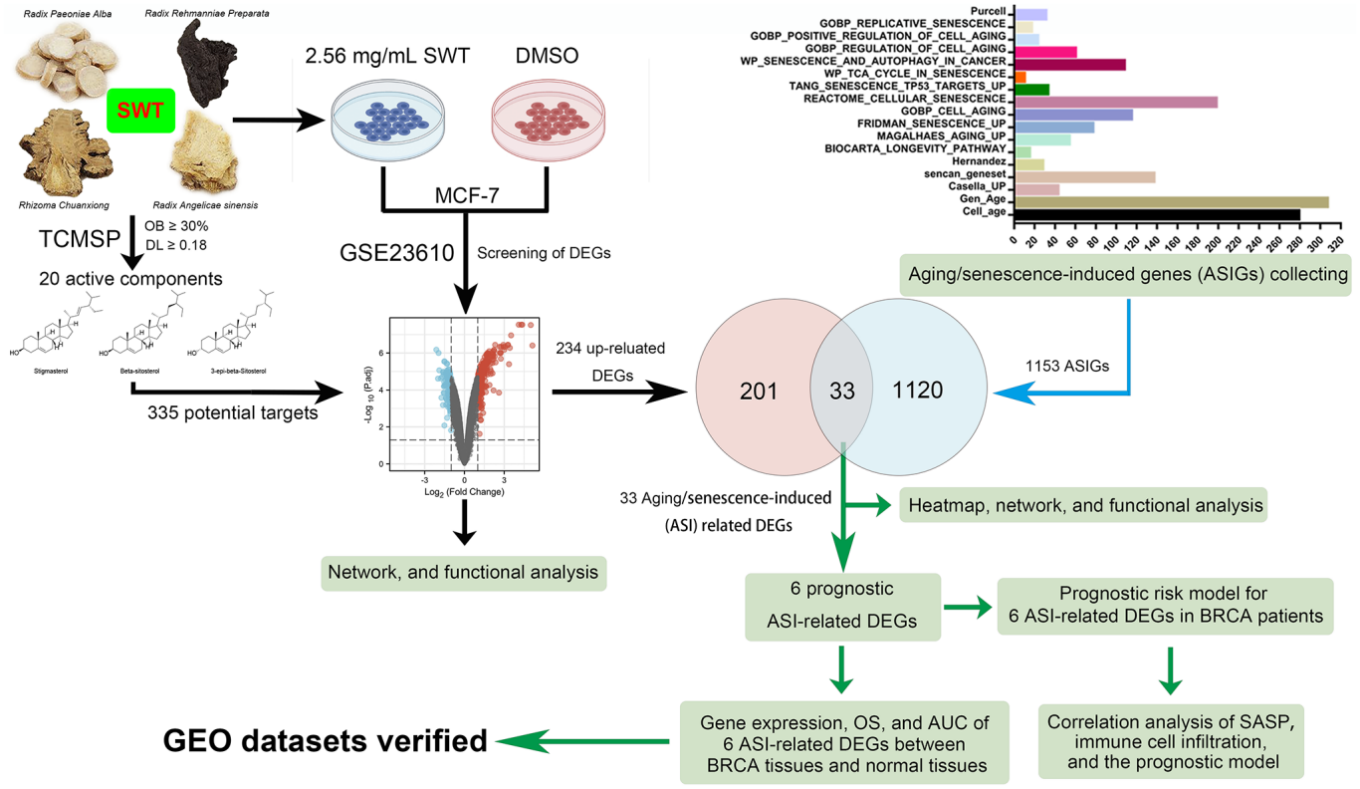
Flowchart of the analytical procedures of the study.

In this study, we explored the active components of SWT and investigated data on gene transcription after treatment of MCF-7 cells with SWT using an open-source database. We then explored the key targets and pharmacological mechanisms underlying the effects of SWT from the perspective of TCM-induced cellular senescence using various biological databases and biocomputational methods. The clinical prognosis and characteristics associated with these key targets were further explored to reveal the value of the clinical application of SWT for the prevention or treatment of breast cancer. Finally, the SASP and immune infiltration were analyzed together to explore whether SWT could be developed for use in combination with immunotherapy. The research results provide new information for the development and application of SWT. The overall study flowchart is shown in Figure 1.

## RESULTS

### Active components of SWT

**Figure 2:**
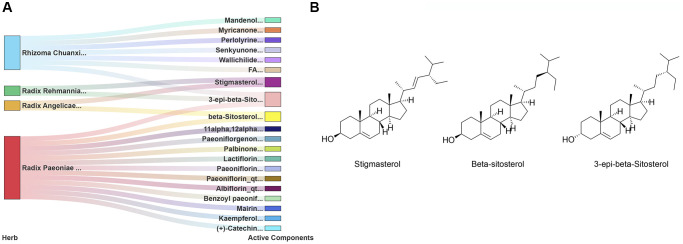
**Identification of 20 active components of SWT.** (**A**) Herb-active component network. (**B**) Chemical structures of 3 key active compounds.

SWT is produced by mixing four herbs, namely, *Radix Angelicae sinensis*, *Rhizoma Chuanxiong*, *Radix Paeoniae Alba*, and *Radix Rehmanniae Preparata,* in equal proportions. According to the two criteria of drug likeness (DL) ≥0.18 and oral bioavailability (OB) ≥ 30%, a total of 20 active components of SWT were identified in the TCMSP (Supplementary Table 1). *Radix Angelicae sinensis*, *Rhizoma Chuanxiong*, *Radix Paeoniae Alba*, and *Radix Rehmanniae Preparata* in SWT contain 2, 7, 13, and 2 active ingredients, respectively. A network diagram of the active compounds of these herbs is shown in Figure 2A. *Radix Angelicae sinensis* and *Radix Paeoniae Alba* all contain beta-sitosterol, *Radix Angelicae sinensis* and *Radix Rehmanniae Preparata* contain stigmasterol, and *Radix Paeoniae Alba*, *Rhizoma Chuanxiong* and *Radix Rehmanniae Preparata* all contain 3-epi-beta-sitosterol. The chemical structures of these active compounds are shown in Figure 2B.

### Identification of DEGs in SWT-treated MCF-7 cells

**Figure 3:**
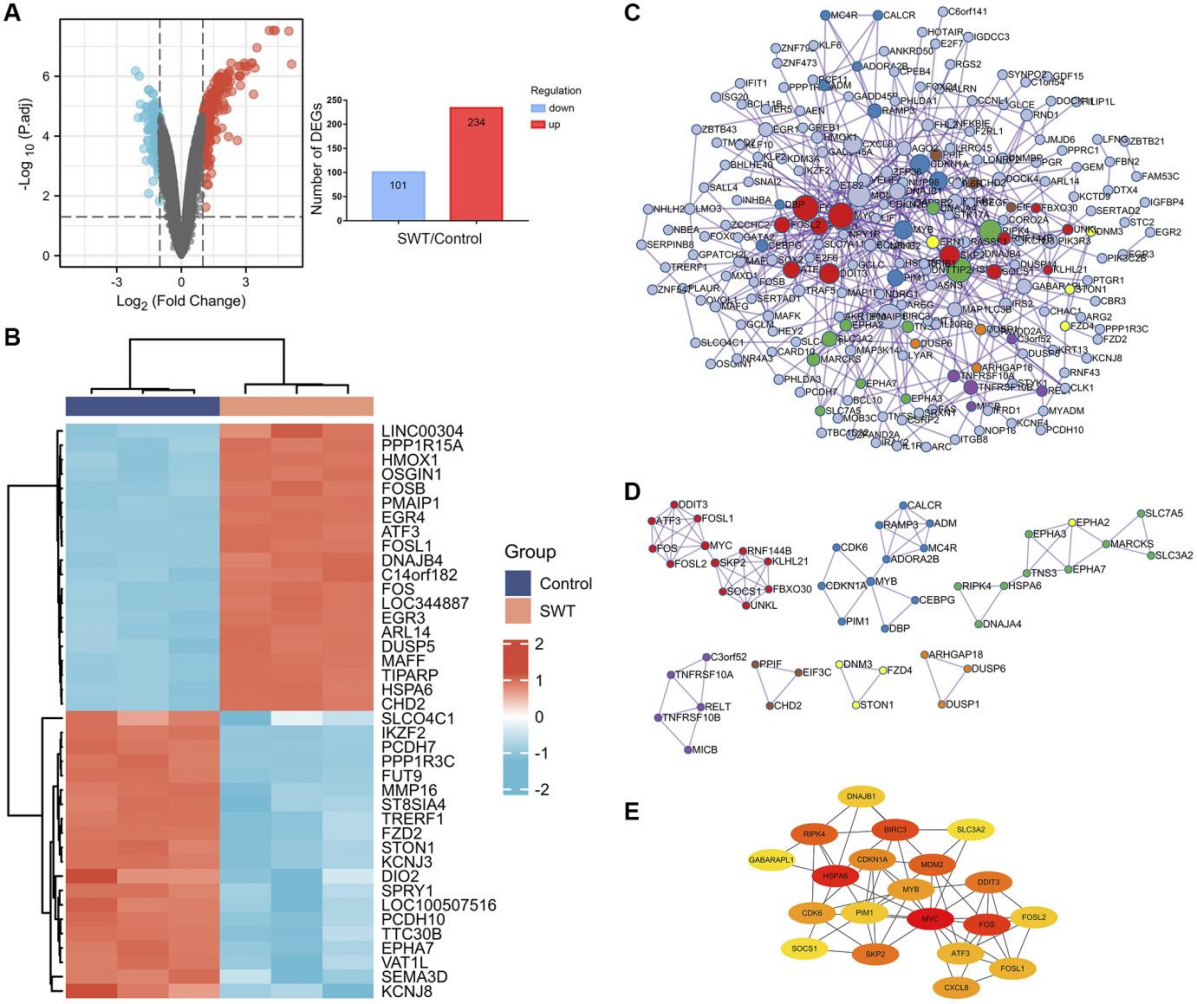
**Differential gene expression patterns and network analysis of SWT-treated MCF7 cells.** (**A**) Volcano plot (left) showing the gene expression patterns of SWT-treated MCF-7 samples. Red and blue represent upregulated genes (logFC ≥ 1) and downregulated genes (logFC ≤ -1), respectively, while gray indicates genes with no significant difference in expression. In addition, the respective numbers of significantly regulated genes are presented in histograms (right). (**B**) Heatmap analysis of the top 20 up- or downregulated DEGs. (**C**) PPI network analysis of DEGs. (**D**) MCODE module for gene clustering analysis. (**E**) Hub gene analysis of DEGs.

The Gene Expression Omnibus (GEO) dataset GSE23610 was analyzed to identify DEGs between DMSO-treated MCF-7 cells and 2.56 mg/mL SWT-treated MCF-7 cells. The results are shown as a volcano plot and histogram. After SWT treatment, there were 335 DEGs, of which 234 were upregulated and 101 were downregulated (Figure 3A, Supplementary Table 2). In addition, the top 20 up- and downregulated DEGs are shown in subsets in heatmaps (Figure 3B). The results showed that after SWT treatment, the expression levels of PPP1R15A, HMOX1, FOSB, PMAIP1, EGR4, ATF3, FOSL1, FOS, HSPA6, DUSP5, and other genes were significantly upregulated and the expression levels of SLCO4C1, IKZF2, FUT9, MMP16, DIO2, STON1, and other genes were significantly downregulated in MCF-7 cells (*P* < 0.05).

### Protein–protein interaction network, module, and hub gene analysis of DEGs in SWT-treated MCF-7 cells

We used the Metascape online database to construct protein–protein interaction (PPI) networks of 335 DEGs, which contained 218 nodes and 507 edges (Figure 3C). Then, seven hub subnetworks of the PPI network were filtered through the MCODE plug-in (Figure 3D). Among them, MCODE 1 contained the FOS, FOSL1, FOSL2, MYC, ATF3, DDIT3, SKP2, SOCS1, UNKL, FBXO30, KLHL21, and RNF144B genes. MCODE 2 contained CDK6, CDKN1A, MYB, PIM1, DBP, CEBPG, ADORA2B, ADM, RAMP3, and CALCR. Furthermore, we screened the top 20 hub genes and performed a topological analysis of this PPI network, and the results showed that CDKN1A, PIM1, SKP2, CXCL8, SOCS1, and CDK6 play key roles in this network (Figure 3E).

### Enrichment analysis of the DEGs in SWT-treated MCF-7 cells

We performed Gene Ontology (GO) and Kyoto Encyclopedia of Genes and Genomes (KEGG) pathway analyses to explore the functions and pathways of the DEGs that are involved in the effect of SWT on breast cancer. The 335 DEGs identified in this study were associated with 1849 GO terms and 59 KEGG pathways (Supplementary Tables 3 and 4). We used a bubble plot to display the top 20 GO/KEGG enrichment analysis results. The larger the ordinate value in the bubble chart, the more significant the corresponding GO/KEGG outcome was. The abscissa represents the normalized upregulation and downregulation value (the ratio of the difference between the number of upregulated genes and the number of downregulated genes to the total number of differential genes). The higher the value is, the higher the number of upregulated genes enriched in the GO/KEGG pathway results; conversely, the lower the value is, the higher the number of downregulated genes enriched in the GO/KEGG pathway results.

**Figure 4:**
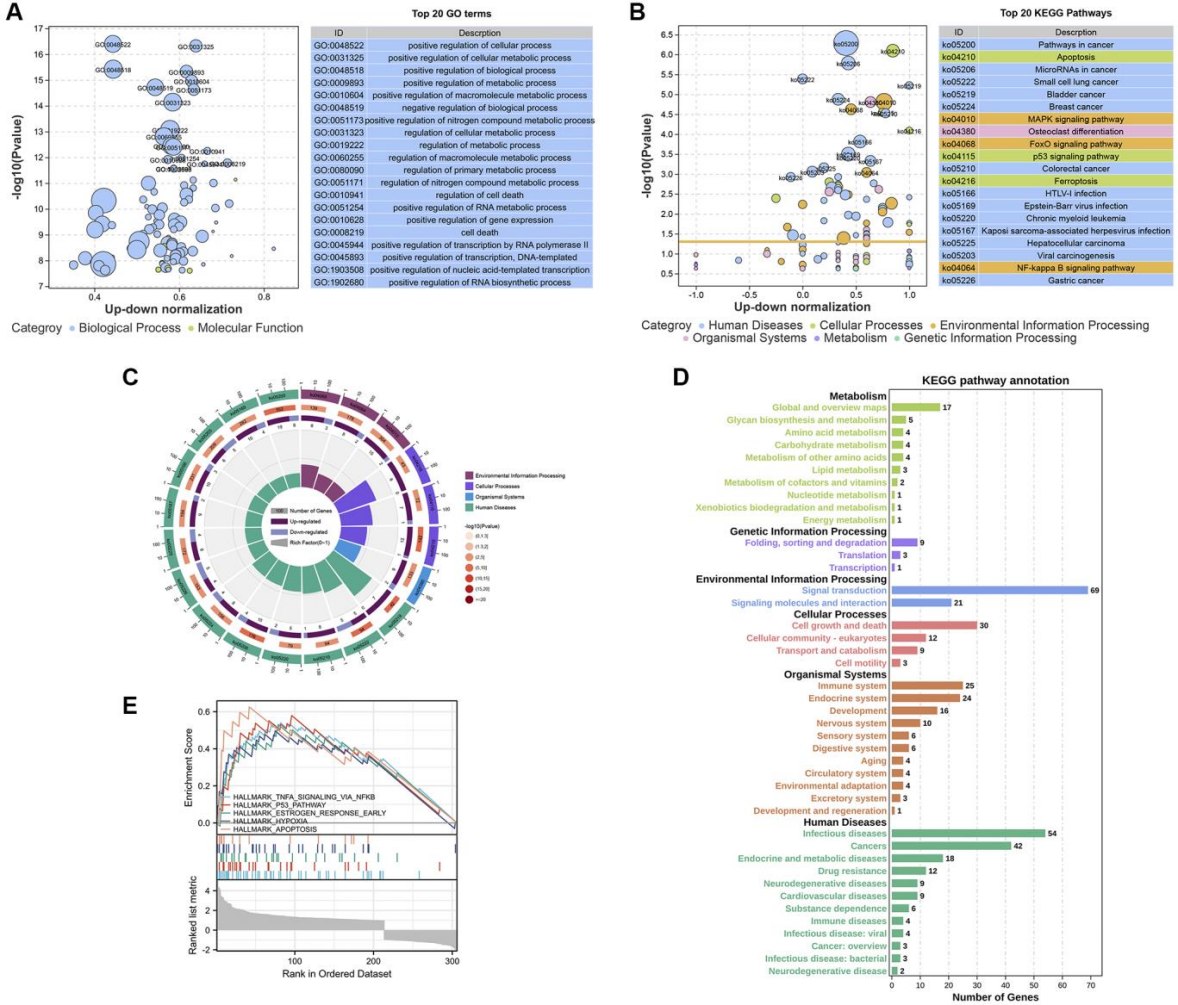
**Enrichment analysis of the DEGs in SWT-treated MCF-7 cells.** (**A** and **B**) Bubble plot showing the top 20 GO and KEGG enrichment analysis results. The larger the ordinate value in the bubble chart, the more significant the corresponding GO or KEGG result is. The abscissa represents the normalized upregulation and downregulation value (the ratio of the difference between the number of upregulated genes and the number of downregulated genes to the total number of differential genes). The higher the value is, the higher the number of upregulated genes enriched in the GO/KEGG pathway results; conversely, the lower the value is, the higher the number of downregulated genes enriched in the GO/KEGG pathway results. (**C**) Secondary classification of the top 20 KEGG pathways. (**D**) Secondary classification of all KEGG pathways. (**E**) GSEA of the DEGs.

The top 20 GO enrichment analysis results revealed that these DEGs were mainly enriched in the following biological processes (Figure 4A): “positive regulation of cellular process (GO:0048522)”, “positive regulation of cellular metabolic process (GO:0031325)”, “positive regulation of biological process (GO:0048518)”, “positive regulation of metabolic process (GO:0009893)”, and “positive regulation of macromolecule metabolic process (GO:0010604)”. In the KEGG pathway enrichment analysis, the results showed that SWT mainly exerts its effects on BC through the following pathways: “Apoptosis (ko04210, *P* value = 5.17E-07)”, “MAPK signaling pathway (ko04010, *P* value = 1.53E-05”, “FoxO signaling pathway (ko04068, *P* value = 2.37E-05)”, “p53 signaling pathway (ko04115, *P* value = 2.83E-05)”, “NF-kappa B signaling pathway (ko04064, *P* value = 0.000912974)”, “Cell cycle (ko04110, *P* value = 0.00159648)”, and “Cellular senescence (ko04218, *P* value = 0.002102546)”. The top 20 KEGG pathways are presented with bubble graphs (Figure 4B).

These 20 KEGG pathways were mainly divided into 4 categories, including environmental information processing, cellular processes, organismal systems, and human diseases (Figure 4C). Next, we conducted a secondary classification of all KEGG pathways, and the results are shown in Figure 4D. In the category of cellular process, the KEGG pathways were mainly enriched in cell growth and death, cellular community-eukaryotes, transport and catabolism, and cell motility. SWT mainly regulates cellular processes via the following KEGG pathways: “Apoptosis (ko04210, *P* value = 5.17E-07)”, “p53 signaling pathway (ko04115, *P* value = 2.83E-05)”, “Cell cycle (ko04110, *P* value = 0.00159648)”, and “Cellular senescence (ko04218, *P* value = 0.002102546)”. Moreover, we performed GSEA on these DEGs to screen significantly enriched pathways based on the hallmark gene set background. The results showed that DEGs were enriched in 5 significant pathways, including TNFα signaling via NF-kappa B, the p53 pathway, the early estrogen response pathway, hypoxia, and apoptosis (*P* < 0.05, Figure 4E).

### Identification of aging/senescence-induced genes and enrichment analysis

**Figure 5:**
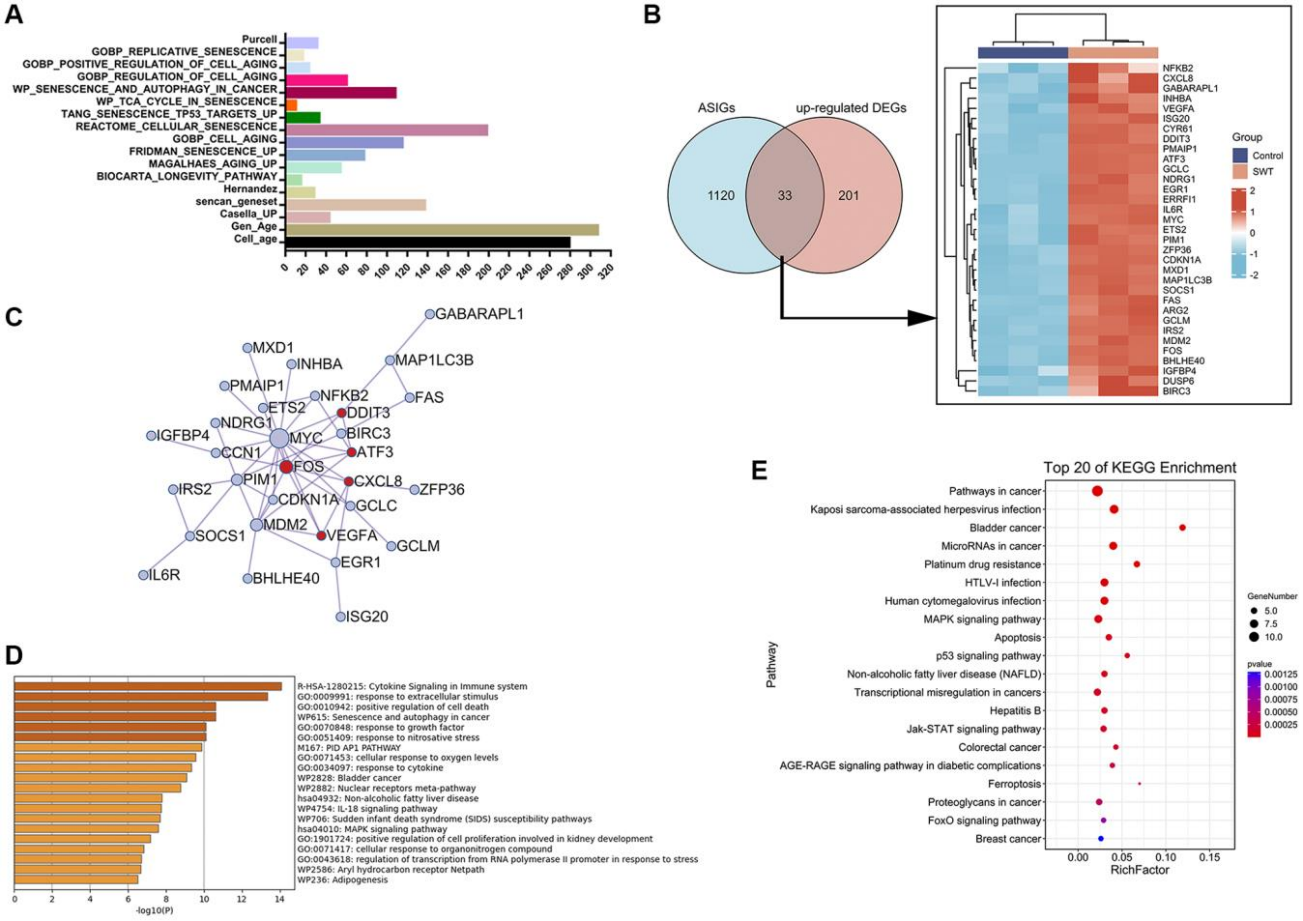
**Venn diagram, network, and enrichment analyses of cellular aging/senescence-induced genes in SWT-treated MCF7 cells.** (**A**) Bar graph representing 1153 aging/senescence-induced genes identified in 17 databases or studies. (**B**) Venn diagram and heatmap analysis of 33 ASIGs that were significantly upregulated by SWT. (**C**) PPI network analysis of 33 ASI-related DEGs. (**D**) Top 20 enriched terms associated with 33 ASI-related DEGs by Metascape database. (**E**) Top 20 KEGG pathways associated with 33 ASI-related DEGs.

As shown in Figure 5A, 1535 aging/senescence-induced genes (ASIGs) were identified in 17 databases or studies, and a total of 1153 genes remained after deduplication. Enrichment analysis was performed to further investigate the GO and KEGG pathways associated with the 1153 ASIGs (Supplementary Figure 1 and Supplementary Table 5). As expected, the top 20 KEGG enrichment results for the 1153 ASIGs showed that these genes were markedly enriched in the cell cycle and cellular senescence-related pathways. Interestingly, a total of 156 KEGG pathways (*P* value < 0.05) enriched by the 1153 AISGs were compared with a total of 59 KEGG pathways (*P* value < 0.05) enriched by the 335 DEGs in SWT-treated breast cancer cells, and 58 pathways overlapped. These pathways were highly enriched in pathways related to tumor proliferation and cellular senescence, including the cell cycle, cellular senescence, MAPK signaling pathway, apoptosis, p53 signaling pathway, and PI3K-Akt signaling pathway. GO enrichment analysis of the ASIGs revealed enrichment of biological processes notably related to cellular response to stress, cell aging, apoptotic process, metabolic process, and cell death, confirming the important role of cellular senescence in tumor progression.

### Identification of aging/senescence-induced DEGs and enrichment analysis

We next compared the upregulated DEGs in the SWT-treated samples with ASIGs to comprehensively characterize the expression pattern of ASIGs in the SWT-treated samples. The results of Venn analysis showed that SWT could upregulate the expression levels of 33 ASIGs in breast cancer (Figure 5B). Subsequently, we constructed a PPI network of these 33 ASI-related DEGs using the Metascape database to assess the correlation and complexity of these 33 genes (Figure 5C). The results showed that MYC, FOS, MDM2, and SOCS1 have greater degrees of involvement, indicating that these genes play a more critical role in this network. GO and KEGG analyses were performed to elucidate the biological processes to which the 33 ASI-related DEGs are related. As expected, the 33 DEGs were markedly enriched in cell death, apoptosis, and cellular senescence-related pathways. These include “GO:0010942: positive regulation of cell death” and “WP615: senescence and autophagy in cancer”, which were identified in the Metascape database analysis (Figure 5D). The “MAPK signaling pathway”, “Apoptosis”, “Cell cycle”, “Cellular senescence”, and “p53 signaling pathway” KEGG pathways were identified by the OmicShare tool analysis (Figure 5E and Supplementary Table 6).

### Identification of aging/senescence-induced DEGs related to prognosis

**Figure 6:**
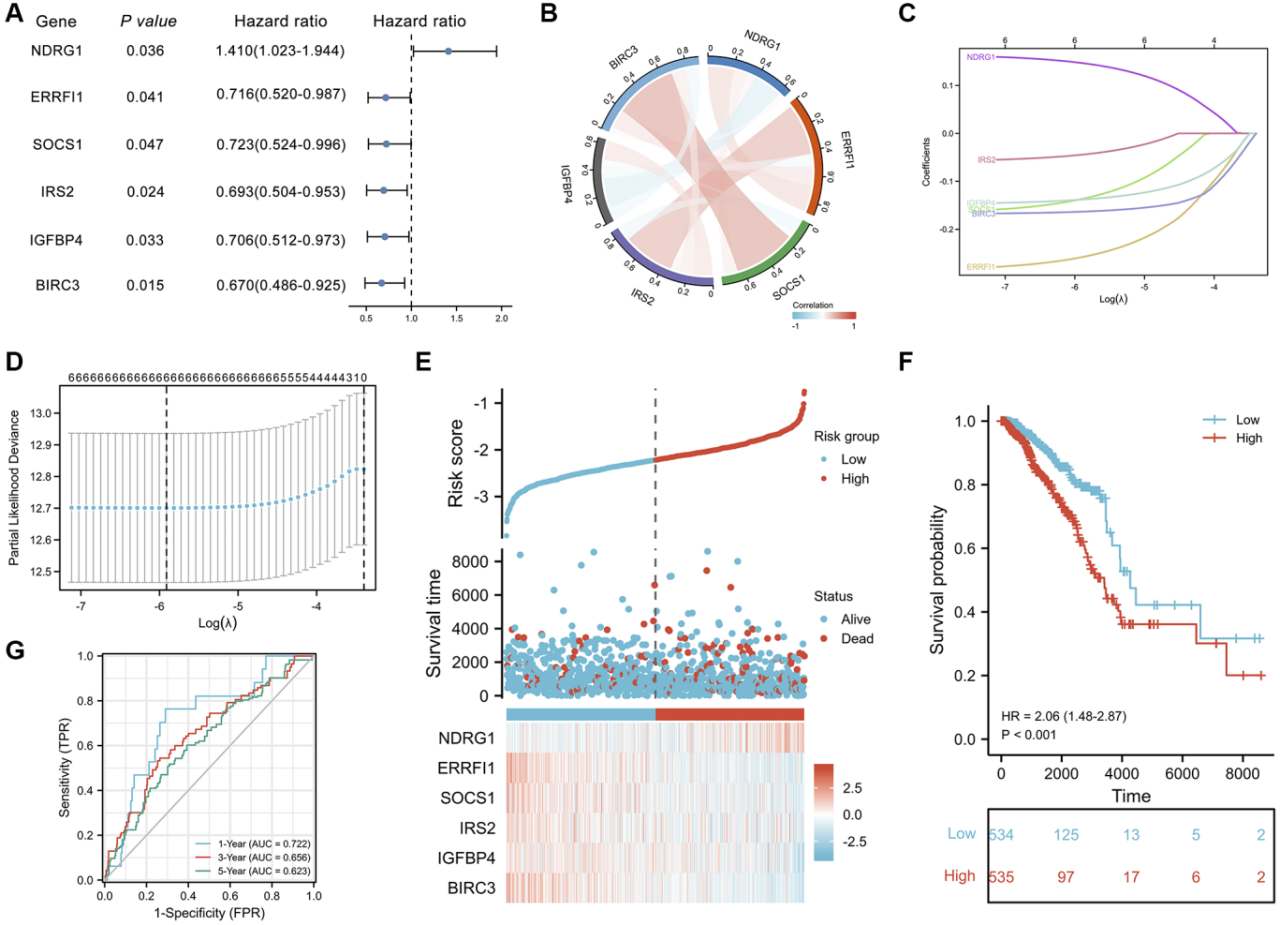
**Prognostic analysis of ASI-related DEGs and establishment of a prognostic model.** (**A**) Forest plot of the univariate Cox analysis of 6 ASI-related DEGs. (**B**) Correlation network of 6 ASI-related DEGs. (**C**) LASSO coefficient profiles of 6 ASI-related DEGs. (**D**) Cross-validation for tuning parameter selection in the LASSO regression. (**E**) The distribution of risk scores, gene expression levels, and survival status of BRCA patients in the training cohort. (**F**) Kaplan–Meier curves of the OS of all the BRCA patients in the TCGA cohort based on the risk score. (**G**) Time-dependent ROC curve analysis of the prognostic model (1, 3, and 5 years).

The clinical characteristics of the breast invasive carcinoma (BRCA) patients in the TCGA datasets are shown in Supplementary Table 7. Next, a univariate Cox proportional hazard regression analysis was initially performed to identify ASI-related DEGs associated with OS (Supplementary Table 8). A total of 6 ASI-related DEGs were significantly associated with OS (*P* < 0.05, Figure 6A). Five of the six genes (ERRFI1, SOCS1, IRS2, IGFBP4, and BIRC3) were considered protective factors (*P* < 0.05, HR < 1), while NDRG1 was considered a risk factor (*P* < 0.05, HR > 1). In addition, the expression levels of 4 prognostic ASI-related DEGs, except NDRG1 and IGFBP4, were positively correlated with each other (Figure 6B).

### Construction of prognostic risk scores with aging/senescence-induced-related DEGs identified from a TCGA dataset

The 6 aging/senescence-induced related DEGs mentioned above were analyzed by least absolute shrinkage and selection operator (LASSO) Cox regression analysis to establish a cellular senescence-related signature for predicting survival. Through LASSO Cox regression analysis, the six ASI-related DEGs were used to establish a risk score to predict the OS of BRCA patients in the TCGA training set (Figure 6C and 6D). The risk score of every patient was then calculated using a formula, and patients from the TCGA training set were then divided into low- and high-risk groups according to the median risk score. The risk plot distribution in the TCGA set is shown in Figure 6E. Additionally, a heatmap showing the expression profiles of 6 ASI-related DEGs in the low- and high-risk groups is presented. Kaplan–Meier survival analysis demonstrated that the overall survival of the low-risk group was significantly better than that of the high-risk group (*P* < 0.001, HR = 2.06(1.48-2.87), Figure 6F). Time-dependent receiver operating characteristic (ROC) analysis was performed and revealed good performance of the risk score in predicting 1-, 3-, and 5-year OS, with areas under the curve (AUCs) of 0.722, 0.656, and 0.623, respectively (Figure 6G).

### Six aging/senescence-induced DEGs are associated with changes in the SASP and immune cell infiltration

**Figure 7:**
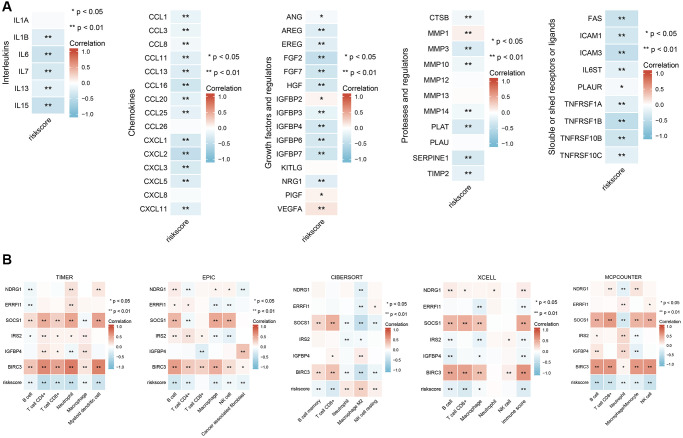
**Correlation analysis of six ASI-related DEG expression with SASP-related factor expression and immune cell infiltration in BRCA patients.** (**A**) Correlation analysis between the expression of different types of SASP-related factors and risk score. (**B**) Correlation analysis between risk scores and infiltration levels of different immune cells estimated by TIMER, EPIC, XCELL, CIBERSORT, and MCPCOUNTER. ^*^, and ^**^ represent *P* < 0.05, and *P* < 0.01, respectively.

One of the characteristics of senescent cells is that they exhibit a proinflammatory senescence-associated secretion phenotype and can secrete inflammatory cytokines, proteases, chemokines, and growth factors that affect the tissue microenvironment in different ways [16]. The SASP can have protumor effects and sometimes antitumor effects [17]. There are many remaining questions about the effect of the SASP on tumors, and an in-depth understanding of the SASP will help reveal the mechanism by which SWT affects breast cancer via the induction of senescence. Our results revealed an inverse relationship between the expression of different types of SASP markers and risk values. As shown in Figure 7A, low-risk patients had higher levels of SASP markers, including interleukins (IL1B, IL6, IL7, IL13, and IL15), chemokines (CCL1, CCL3, CCL8, CCL11, CCL13, CCL16, CCL20, CCL25, CXCL1, CXCL2, CXCL3, CXCL5, and CXCL11), growth factors and regulators (ANG, AREG, EREG, FGF2, FGF7, HGF, IGFBP3, IGFBP4, and NRG1), proteases and regulators (CTSB, MMP3, MMP10, MMP14, PLAT, SERPINE1, and TIMP2), and soluble or secreted receptors or ligands (FAS, ICAM1, ICAM3, IL6ST, PLAUR, TNFRSF1A, TNFRSF1B, TNFRSF10B, and TNFRSF10C). Notably, the lower the risk score was, the lower the levels of secreted PIGF, VEGF, and MMP1 were.

We analyzed the correlations between the abundance of immune cell markers and the expression of 6 ASI-related DEGs to better understand their role in immune responses. Next, we characterized the immune AIS-related DEG profile by assessing infiltrating immune cell populations using RNA-seq data and TIMER2.0 (Figure 7B). The correlation analysis showed that the levels of infiltrating B cells, CD8^+^ T cells, CD4^+^ T cells, NK cells, and macrophages were negatively correlated with the risk score, while the levels of NK resting cells and M2 macrophages were positively correlated with risk scores. Moreover, we found that IGFBP4, BIRC3, and SOCS1 expression levels were significantly and strongly positively correlated with numbers of infiltrating B cells, CD4^+^ T cells, CD8^+^ T cells, macrophages, and NK cells (*P* < 0.05).

### Screening of key therapeutic targets of SWT in breast cancer based on cellular senescence

**Figure 8:**
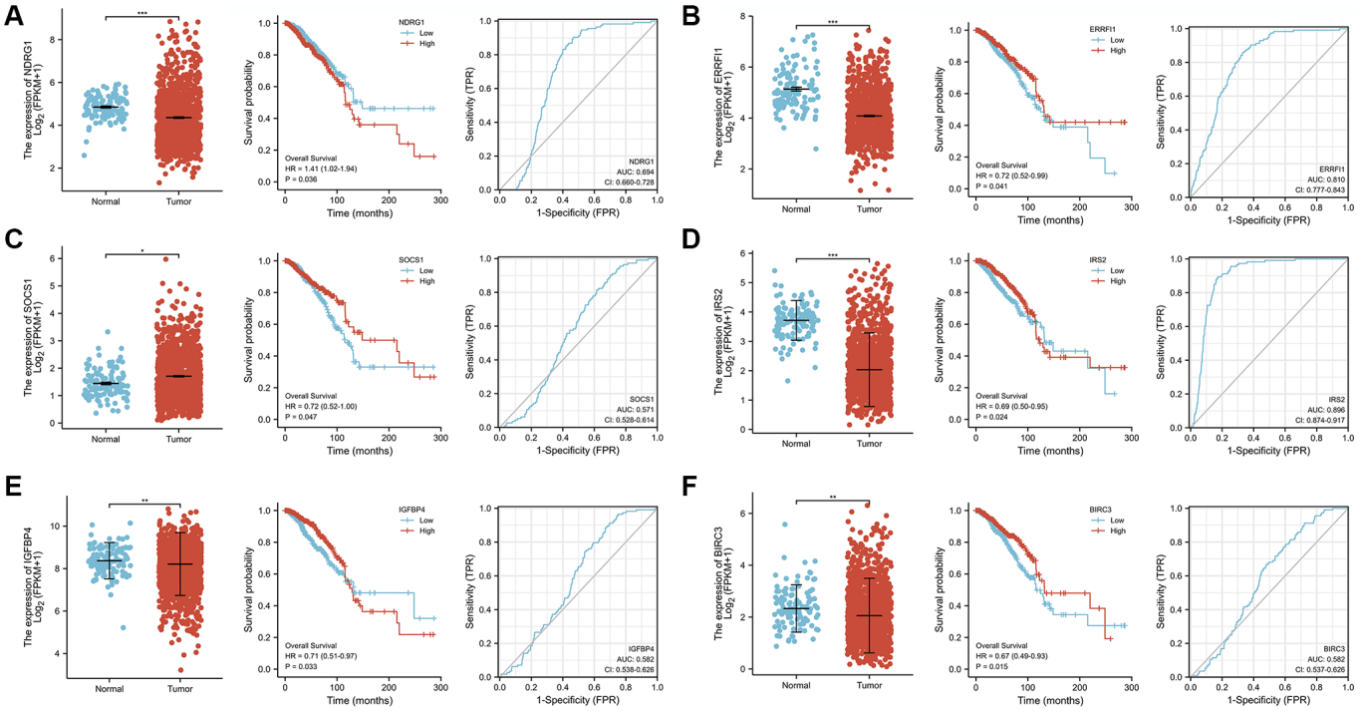
**Analysis of gene expression, OS, and AUC of 6 ASI-related DEGs in BRCA tissues and adjacent tissues.** (**A**) NDRG1. (**B**) ERRFI1. (**C**) SOCS1. (**D**) IRS2. (**E**) IGFBP4. (**F**) BIRC3.

The transcriptional levels of 6 AIS-related DEGs in BRCA patient tumor tissues and adjacent normal tissues in the TCGA-BRCA database were quantitatively analyzed to further explore their expression patterns. The results showed that the transcriptional expression levels of NDRG1 (*P* < 0.001), ERRFI1 (*P* < 0.001), IRS2 (*P* < 0.001), IGFBP4 (*P* < 0.01), and BIRC3 (*P* < 0.01) were significantly decreased in tumor tissues, while SOCS1 (*P* < 0.05) expression was increased (Figure 8A–8F). The overall survival analysis showed that patients with high expression levels of ERRFI1 (*P* = 0.041, HR = 0.72(0.52–0.99)), SOCS1 (*P* = 0.047, HR = 0.72(0.52–1.00)), IRS2 (*P* = 0.024, HR = 0.69(0.50–0.95)), IGFBP4 (*P* = 0.033, HR = 0.71(0.51–0.97)), and BIRC3 (*P* = 0.015, HR = 0.67(0.49–0.93)) had prolonged OS compared with patients with low expression levels. Conversely, NDRG1 (*P* = 0.035, HR = 1.41(1.02–1.94)) expression was negatively correlated with prognosis. Next, the time-dependent receiver operating characteristic results showed that the ERRFI1 (AUC = 0.810) and IRS2 (AUC = 0.896) genes showed better predictive performance, and the rest showed moderate predictive performance.

**Figure 9:**
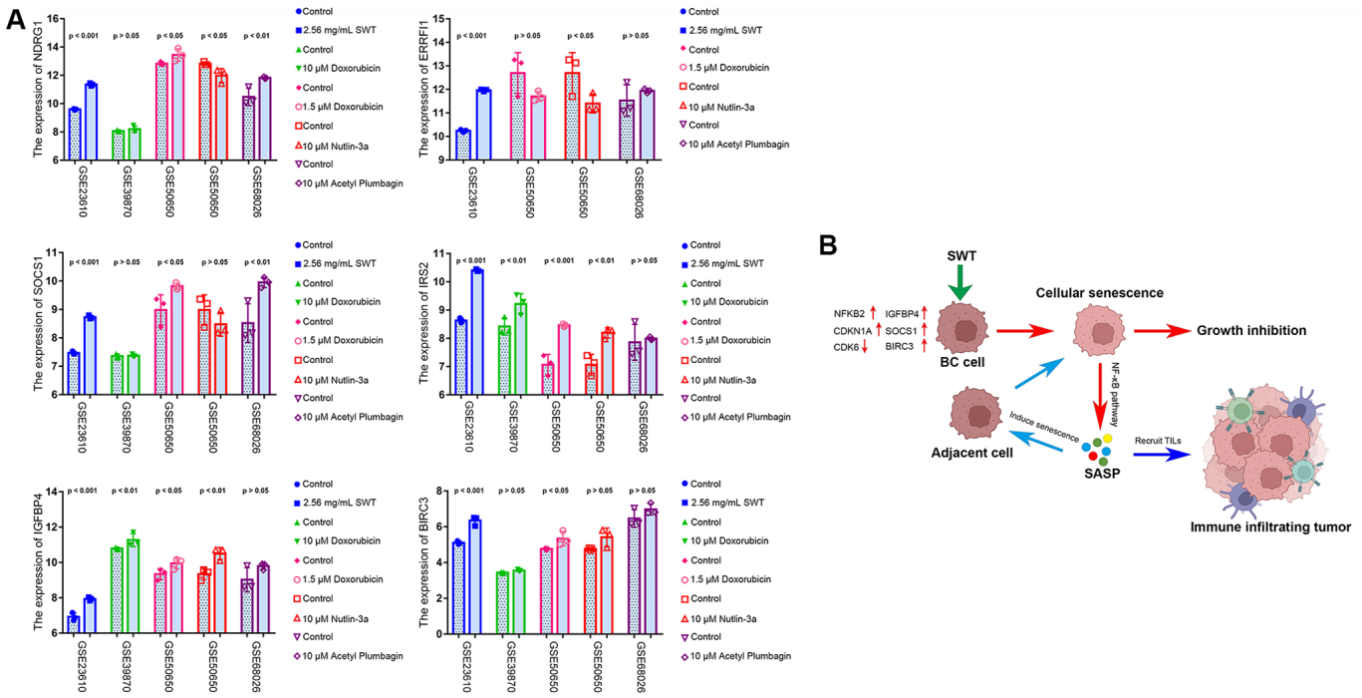
**GEO dataset validation and proposed mechanisms underlying SWT-mediated induction of senescence in breast cancer cells.** (**A**) Validation of the expression patterns of 6 ASI-related DEGs in MCF-7 cells treated with different drugs via analysis of different GEO datasets. (**B**) Graphical abstract showing the mechanisms underlying SWT-mediated induction of cellular senescence in BC.

Next, to explore the expression patterns of these six ASI-related DEGs in MCF-7 cells treated with SWT, we used the GEO database (GSE23610) to quantitatively analyze changes in their transcriptional level. Doxorubicin (GSE39870, GSE50650), a drug that is commonly used for breast cancer treatment, has been used in aging studies in recent years [18]. In addition, nutlin-3a (GSE50650) is often used in studies of cellular senescence [19], and acetyl plumbagin (GSE68026) is used in studies on breast cancer [20]. Therefore, we simultaneously explored the expression patterns of these 6 genes in MCF-7 cells treated with doxorubicin, nutlin-3a or acetyl plumbagin to determine whether the mechanism by which SWT affects breast cancer is similar to that of these drugs (Figure 9A**)**. As expected, the results of treatment with SWT or other drug were similar, with SWT, 1.5 μM doxorubicin, 10 μM nutlin-3a, or 10 μM acetyl plumbagin upregulating the transcription of six ASI-related DEGs compared to the controls.

## DISCUSSION

Since the pathological mechanism of BC and the multicomponent and multitarget characteristics of SWT are very complex, a single experiment cannot systematically reveal the pharmacological mechanism by which SWT affects BC. An efficient method that integrates systems biology and *in silico* technologies is more suitable for such studies. Therefore, we attempted to use this integrated strategy to explore how SWT exerts pharmacological anti-BC effects by inducing cellular senescence.

According to the herb-active component network diagram, the roles of stigmasterol, β-sitosterol, and 3-epi-beta-sitosterol, which are three hub components, are substantial. We hypothesize that these active components may be an important basis for the synergistic anti-BC effect of the 4 herbs in SWT. Previous studies have shown that β-sitosterol can inhibit the growth of MCF-7 human breast cancer cells by activating Fas signaling and regulating apoptosis [21]. Stigmasterol is a plant sterol that has been shown to exert anticancer effects against various cancers, including breast cancer [22]. Although the anticancer activity of 3-epi-beta-sitosterol has not yet been reported in the literature, phytosterols are structurally similar; they appear to exert anticancer effects by inhibiting cancer cell growth, invasion and metastasis, promoting cancer cell apoptosis, and other mechanisms [23]. In addition to phytosterols, other active components have been shown to exert anti-BC effects. Kaempferol induces cell cycle arrest, apoptosis, and DNA damage, thereby inhibiting the proliferation of BC cells [24]. Paeoniflorin inhibits the proliferation and invasion of BC cells by inhibiting the Notch-1 signaling pathway [25]. Overall, these active components of SWT exert strong pharmacological anti-breast cancer effects.

Diverging from the conventional network pharmacology approach of mining potential TCM targets in a target prediction database, we used the public GEO dataset to analyze the gene expression profile of the BC cell line MCF-7 after treatment with SWT in order to increase reliability. A total of 234 significantly upregulated DEGs and 101 significantly downregulated DEGs were identified, suggesting that SWT may exert its anti-BC effects through the effects of multiple active components on these targets. Moreover, the PPI results indicated that SWT inhibits BC through a complex molecular network. Some DEGs with high topological parameters were defined as hub genes, including genes that play important roles in cell cycle arrest in senescence (CDK6, CDKN1A) [26], genes that are key targets of cancer therapy (PIM1, SKP2) [27, 28], genes related to the tumor microenvironment (CXCL8) [29], and genes that encode tumor suppressors (SOCS1) [30].

We analyzed 234 upregulated DEGs and 1153 ASIGs to better elucidate the mechanism by which SWT induces BC cell senescence. The results showed that SWT might induce BC cell senescence by upregulating the expression of these 33 ASIGs. An enrichment analysis of these 33 aging/senescence-induced related DEGs revealed that SWT-induced cellular senescence is mainly regulated by cellular senescence-related processes or pathways, including senescence and autophagy in cancer, the MAPK signaling pathway, apoptosis, the p53 signaling pathway, cellular senescence, the cell cycle, and the FoxO signaling pathway. Recent studies have shown that these biological processes and signaling pathways are related to cellular senescence [31–34].

Next, the association of these 33 ASI-related DEGs with the prognosis and clinical characteristics of BRCA patients was explored to determine the value of the clinical application of SWT for BC treatment. Notably, we found 6 ASI-related DEGs, namely, NDRG1, ERRFI1, SOCS1, IRS2, IGFBP4, and BIRC3, that were significantly associated with the overall survival of BRCA patients. These 6 signature genes have been reported to be regulators of cellular senescence in various cancers and play important roles in tumor development. ERRFI1 is involved in the induction of cellular senescence and tumor suppressor events [35]. Wen-Jing Lu et al. [36] showed that NDRG1 is involved in regulating cellular senescence in hepatocellular carcinoma. Studies by Viviane Calabrese et al. [37] demonstrated that SOCS1 is sufficient to induce p53-dependent senescence in fibroblasts and revealed the mechanism by which SOCS1 functions as a tumor suppressor. Increased IRS2 levels in a mouse model of Huntington’s disease significantly shortens lifespan and increases neuronal oxidative stress and mitochondrial dysfunction [38]. A study by Nicola Alessio et al. [39] revealed a significant increase in senescent cell numbers in the lungs, hearts, and kidneys of mice intraperitoneally administered IGFBP4 twice weekly for two months. Moreover, studies have shown that senescent cells upregulate the expression of the antiapoptotic protein BIRC3 [40].

Subsequently, 6 ASI-related DEGs were used to construct a risk score through a LASSO Cox regression model, and this risk score showed good performance in predicting prognosis in the training set. Based on the Kaplan–Meier method, the overall survival trend of low-risk BRCA patients was found to be higher than that of high-risk patients. The 1-year AUC of this prognostic model was 0.722, indicating high predictive accuracy. In the 6 ASI-related DEG model, ERRFI1, SOCS1, IRS2, IGFBP4, and BIRC3 acted as protective factors, whereas NDRG1 acted as a risk factor. Moreover, a significant increase in the RNA transcription levels of ERRFI1, SOCS1, IRS2, IGFBP4, and BIRC3 was observed in the low-risk group compared with the high-risk group, demonstrating the protective effect of the high RNA transcription levels of these genes on the prognosis of BRCA patients. Consistent with previous reports, NDRG1 was found to be a risk factor for BC. NDRG1 has been shown to drive tumor progression and brain metastasis in aggressive breast cancer [41]. ERRFI1 is a tumor suppressor in tumor cells *in situ*, and its expression is required to prevent apoptosis and BC cell metastasis [42]. Additionally, reduced ERRFI1 expression is associated with poor prognosis in breast cancer patients [43]. IGFBP4 mRNA expression is an independent prognostic factor in breast cancer patients, and its mRNA expression level is positively correlated with estrogen receptor status [44]. Furthermore, breast cancer patients with high IGFBP4 mRNA expression had better disease-free survival and overall survival rates than patients with low expression. Studies have also shown that IGFBP4 interferes with the E2-induced activation of the Akt/PKB pathway and prevents fully hormone-dependent activation of ERα and breast cancer cell growth in an IGF- and IGF-IR-independent manner [45]. Univariate survival analysis showed that patients with low SOCS1 expression levels had a poor prognosis [46]. Higher expression levels of SOCS1 are associated with earlier tumor stages and better clinical outcomes in human breast cancer patients [47]. IRS2 expression is low in ductal carcinoma *in situ* but increases significantly as tumor invasiveness increases [48]. BIRC3 plays a role in preventing rapid mammary involution and promoting survival during tumorigenesis [49].

In the current study, we verified the expression patterns of these genes after drug treatment using GEO datasets. Doxorubicin is one of the most effective drugs for treating early and advanced breast tumors. Recent studies have shown that doxorubicin can induce senescence in tumor cells. Our study showed that the effects of SWT on SOCS1, IRS2, IGFBP4, and BIRC3 gene expression levels were similar to those of doxorubicin in MCF-7 cells [18]. Based on the results of our previous study and other previous studies, we hypothesize that ERRFI1, SOCS1, and IGFBP4 are protective factors in patients with BRCA, and their high expression yields a better prognosis and is beneficial to the clinical outcome of BRCA. To the best of our knowledge, this is the first systematic study of the SWT-mediated induction of cellular senescence in BC treatment that explored the key cellular senescence-inducing genes involved and the clinical value of these genes in BC prognosis.

Importantly, revealing how SWT-induced BC cell senescence affects the tumor microenvironment will help improve tumor outcomes by providing information that is necessary for combining TCM and immunotherapy in the future. In the present study, we found that the risk score was negatively correlated with the levels of infiltrating B cells, CD4^+^ T cells, CD8^+^ T cells, macrophages, and neutrophils in BRCA, whereas resting NK cell levels were positively correlated with the risk score. High levels of tumor-infiltrating lymphocytes (TILs) have been consistently associated with a favorable prognosis in BC. These infiltrates reflect a favorable host antitumor immune response, suggesting that immune activation is important for improved survival outcomes [50]. Our results suggest that SWT may promote immune cell infiltration in BC patients by upregulating the expression levels of SOCS1, IGFBP4, and BIRC3.

Cellular senescence has cell-autonomous and paracrine effects that significantly impact the microenvironment, and senescent cells can be eliminated through an immune response elicited by the SASP that involves both innate and adaptive immunity [17]. To further explore the mechanism underlying immune remodeling caused by increased proportions of senescent cells in tumors, we explored how the 6 ASI-related DEGs induced by SWT alter the SASP and affect the tumor immune microenvironment (TIME), ultimately inhibiting tumor development. The SASP has several positive short-term effects. However, these effects may become detrimental in the long term, promoting the immunosuppressive cancer environment and tumor development. Our results showed that various SASP-related factors secreted by tumor cells were inversely associated with risk scores in the established model. While multiple studies have shown that SASP-related factors can support or inhibit antitumor immune responses, their specific function depends on different circumstances [17, 51]. Once cellular senescence is induced, it is reinforced by the activation of the MAPK pathway and the expression of SASP-related factors [16, 52]. Additionally, loss of the p53 tumor suppressor pathway allows cells to bypass senescence, leading to malignant transformation [53]. Therefore, early tumor cell senescence can serve as a tumor-suppressive mechanism.

In addition, SASP-related factors, including IL-6, CXCL1, and CXCL2, can act via a positive feedback loop to enhance senescence through continued activation of the NF-κB pathway [16, 54, 55]. Furthermore, multiple studies have revealed that SASP-related factors in different cell types can induce senescence and the SASP in adjacent cells, leading to tumor suppression [17]. One benefit of this phenomenon is that it may allow senescence signatures to amplify and trigger immune responses to dysfunctional cells that need to be cleared, since many SASP-related factors are inflammatory. Studies on the molecular mechanism underlying SASP development have shown that different transcription factors regulate the SASP, and NF-κB plays a key role in this response. NF-κB signaling can bind to the promoters of SASP-related factors and thus promote the transcription of some, but not all, SASP-related factors [56]. Additionally, silencing the expression of p65, which is an NF-κB subunit, abolished the SASP and resulted in early cancer recurrence and shorter overall survival times.

Notably, our study showed that SWT could significantly upregulate the expression of NF-κB and significantly regulate the NF-κB signaling pathway, MAPK pathway, and p53 pathway in BC. We hypothesize that SWT induces the senescence of breast cancer cells and that this senescence further accelerates the induction of senescence of other unaffected cells by activating the MAPK pathway and increasing the secretion of SASP-related factors, forming an amplification loop. On the other hand, these SASP-related factors can eliminate cancer cells by enhancing host immune surveillance by recruiting more TIL cells. This anticancer SASP may be regulated by the SWT-mediated activation of the NF-κB and MAPK pathways in BC cells. The proposed mechanism is shown in Figure 9B.

## CONCLUSIONS

In conclusion, we elucidated the potential pharmacological mechanism by which SWT functions in the treatment of BC through open databases and systems biology, and our study, revealed that SWT can suppress tumors by inducing cellular senescence. Analysis of TCGA data showed that SWT could improve the prognosis and clinical outcomes of patients by upregulating the expression levels of SOCS1, IGFBP4 and ERRFI1. Additionally, SWT can induce the development of the anticancer SASP through the NF-κB pathway, and SASP-related factors can further affect the TME and alter tumor progression. In recent years, TCM-induced cellular senescence has become a promising strategy for cancer treatment but is limited by the complexity of TCM components and targets; thus, the gap between research on TCM and Western medicine has increased. Therefore, although this study still has limitations, this study systematically revealed the antitumor effect of SWT and identified novel underlying mechanisms, and it also presented innovative research methods and breakthroughs for TCM research.

## MATERIALS AND METHODS

### Screening the chemical components of SWT

We identified the chemical components of SWT with the Traditional Chinese Medicine System Pharmacology Database (TCMSP). First, Chinese terms such as “Dang Gui”, “Chuan Xiong”, “Bai Shao” or “Shu Di Huang” were entered into the database to determine their components and retrieve their pharmacokinetic data. Here, we selected two pharmacokinetic parameters as screening criteria to determine the active ingredients in these herbs. The herbs that met the criteria of oral bioavailability (OB) ≥30% and drug likeness (DL) ≥0.18 were considered to be the active ingredients of SWT for subsequent analyses [57, 58].

### Publicly available expression datasets

An RNA sequencing (RNA-Seq) expression profile dataset of BRCA patients, which included clinicopathological characteristics and survival data, was downloaded from The Cancer Genome Atlas (TCGA, https://portal.gdc.cancer.gov). The fragments per kilobase million (FPKM) values of the TCGA cohort were then transformed into transcripts per million (TPM) values before further analysis. The GSE23610 (GSM578941, GSM578942, GSM578943, GSM578956, GSM578957, and GSM578958) [11], GSE39870 (GSM980568, GSM980569, GSM980570, GSM980571, GSM980572, and GSM980573) [59], GSE50650 (GSM1225765, GSM1225766, GSM1225767, GSM1225768, GSM1225769, GSM1225770, GSM1225771, GSM1225772, and GSM1225773) [60], and GSE68026 (GSM1661360, GSM1661361, GSM1661362, GSM1661363, GSM1661364, and GSM1661365) datasets were downloaded from the GEO (https://www.ncbi.nlm.nih.gov/geo) database.

### Identification of aging/senescence-induced genes

We followed the protocol described by Dominik Saul et al. [61] to identify aging/senescence-induced genes and characterize the molecular patterns associated with cellular senescence. A total of 1535 aging/senescence-inducing genes were identified in 17 databases or studies, and a total of 1153 genes remained after deduplication. Due to the need to investigate whether SWT suppresses or prevents breast cancer by inducing cellular senescence, we focused on genes whose expression was associated with cellular senescence for this study. Since the expression levels of these genes are upregulated during cellular senescence, they are referred to as aging/senescence-induced genes in this manuscript.

### Identification of differentially expressed genes and aging/senescence-induced related DEGs

DEGs between the control and SWT-treated MCF-7 cells were identified by the R software “limma” package. Significantly DEGs were selected based on a Benjamini–Hochberg-adjusted *P* value <0.05, false discovery rate (FDR) <0.05, and log_2_|Fold Change| > 1. Subsequently, the “ggplot2” and “ComplexHeatmap” packages were used to draw DEG volcano plots and heatmaps, respectively. Then, the ASI-related DEGs were identified from among the DEGs and ASIGs using the Venn tool for subsequent studies.

### Construction and validation of a prognostic model involving ASI-related DEGs and LASSO Cox regression

Prognostic ASI-related DEGs were identified by univariate Cox proportional hazard regression analysis of OS using the “survival” package in the TCGA set (*p* < 0.05). Least absolute shrinkage and selection operator Cox regression was conducted with a random seed using the R packages “glmnet” and “survival” to construct the risk score model that best predicted survival in the training cohort. The standardized expression matrix of candidate prognostic ASI-related DEGs was used as the independent variable in the regression, and the response variables were OS and patient status in the TCGA cohort. A risk score was determined for each patient based on the standardized expression level of each gene and its corresponding regression coefficient. The formula is as follows:


$$RiskScore=\sum_{i=1}^{n}Coefficient_{i}\times expression_{i}$$

The patients were then divided into low-risk and high-risk groups according to the median risk score.

### Correlation among different types of SASP-related factors, immune cell infiltration, and the prognostic model

To further verify the potential importance of tumor cell senescence in breast cancer, different types of SASP-related factors, including representative interleukins, chemokines, growth factors and regulators, proteases and regulators, and soluble or secreted receptors or ligands, were selected based on the results of previous studies, and the correlation between risk values and the levels of these factors was analyzed by the Spearman algorithm [62]. Additionally, we estimated immune cell infiltration in the TCGA RNA-seq cohort based on centralized algorithms in the TIMER2.0 (http://timer.comp-genomics.org/) database, including the TIMER, EPIC, XCELL, CIBERSORT, and MCPCOUNTER algorithms. These data were used to analyze the relationship between the risk scores of BRCA patients and the levels of immune cell infiltration. Furthermore, we explored the correlation between immune cell abundance and ASI-related DEG expression. Pearson correlation analysis was used to elucidate the correlation between ASI-related DEG expression and immune cell infiltration.

### Pathway and functional enrichment analysis

To further analyze the DEGs, KEGG and GO enrichment analyses were performed using the OmicShare tool (https://www.omicshare.com/tools) and Metascape (http://metascape.org/gp/index.html). GO enrichment analysis included annotations from three aspects, including biological process (BP), cytological component (CC) and molecular function (MF). KEGG enrichment analysis mainly predicts signaling pathways that are potentially involved. *P* < 0.05 was considered statistically significant in this study.

Gene set enrichment analysis (GSEA) was performed on the DEGs using the “clusterProfiler” package in R language to identify potential pathways or processes with which these genes are associated on the background of hallmark gene sets. Significantly enriched genes were defined as those with a normalized enrichment score (NES) >1.5 and *P* < 0.05.

### Network construction, key module selection, hub gene identification, and coexpression analysis

A list of DEGs was submitted to the Metascape web tool, with the species limited to “*Homo sapiens*”, to construct a PPI network. Subsequently, the Molecular Complex Detection (MCODE) algorithm was used to identify densely connected network components. This novel graph-theoretical clustering algorithm can reveal densely connected regions in large PPI networks of molecular complexes. Then, the data were imported into Cytoscape software (version 3.7.2) for reprocessing, visualization, hub gene analysis, and topological analysis. The herb-active component (H-AC) network was analyzed and displayed using the OmicShare tool. Additionally, based on TCGA-BRCA gene expression data, an interactive gene correlation network of the prognostic ASI-related DEGs was constructed using the R package “corrplot”. The network was visualized using the R package “circlize”.

### Statistical analysis

The data analysis and graph generation were performed in R version 3.6.3 and GraphPad Prism 7.0. Comparisons between two groups were performed using unpaired Student’s *t* test to analyze the statistical significance of normally distributed variables and the Wilcoxon rank-sum test to estimate the statistical significance of nonnormally distributed variables. Categorical variables were compared using the χ test. Kaplan–Meier survival curves of OS analysis were plotted using the R package “survminer”. Receiver operating characteristic curves for 1-, 3-, and 5-year survival were drawn to assess the diagnostic value of the risk score generated using timeROC. Furthermore, the area under the ROC curve was used to analyze the accuracy of ASI-related DEGs in predicting prognosis. Differences with *P* < 0.05 were considered statistically significant.

### Data availability

Publicly available datasets were analyzed in this study. This data can be found here: TCMSP, TCGA, GEO, etc.

## Supplementary Materials

Supplementary Figure 1

Supplementary Table 1, 4, and 6-8

Supplementary Table 2-3 and 5
