# Supplementary material for: Elucidating the pharmacological mechanism by which Si-Wu-Tang induces cellular senescence in breast cancer via multilevel data integration
Source: Aging (Albany NY). 2022 Jul 19;14(14):5812–37. doi: 10.18632/aging.204185 (PMC9365552; doi:10.18632/aging.204185)
Supplement: Supplementary Figure 1 [file aging-14-204185-s001.pdf]

SUPPLEMENTARY FIGURE

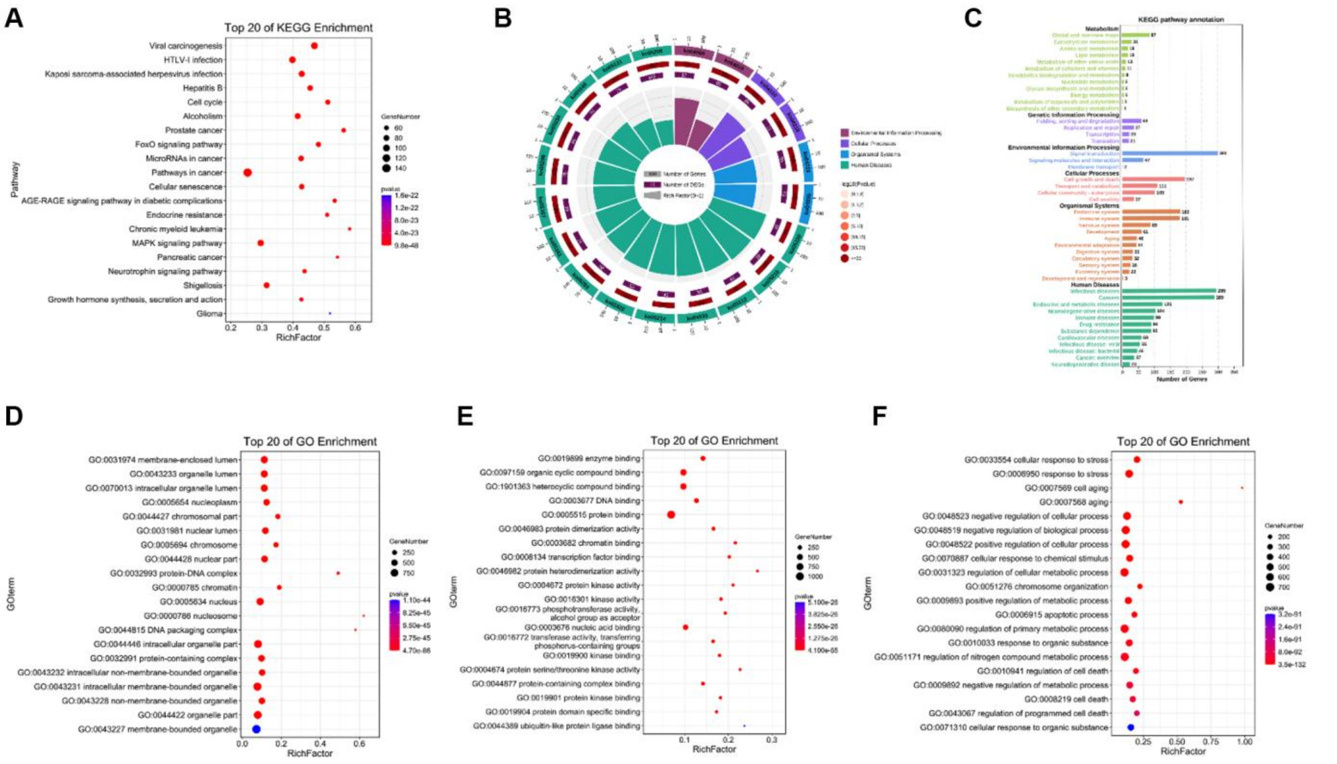

Supplementary Figure 1. GO and KEGG enriched by 1153 aging/senescence-inducing genes. (**A**) The top 20 KEGG pathways are enriched by the 1153 ASIGs. The ratio of genes enriched in each pathway is recorded on the horizontal axis. The size of each bubble indicates the number of genes enriched in each KEGG pathway. The larger the bubble, the more genes are involved in the pathway. The color of each bubble represents the *p*-value of each KEGG path. The redder the color of the term, the smaller the *p*-value. (**B**) These top 20 KEGG pathways were mainly divided into 4 categories, including environmental information processing, cellular processes, organismal systems, and human diseases. (**C**) A secondary classification of all KEGG pathways. (**D**) The top 20 GO_Cellular Component terms enriched by 1153 ASIGs. (**E**) The top 20 GO_Molecular Function terms enriched by 1153 ASIGs. (**F**) The top 20 GO_Biological Process terms enriched by 1153 ASIGs.
