# Supplementary material for: Elucidating the pharmacological mechanism by which Si-Wu-Tang induces cellular senescence in breast cancer via multilevel data integration
Source: Aging (Albany NY). 2022 Jul 19;14(14):5812–37. doi: 10.18632/aging.204185 (PMC9365552; doi:10.18632/aging.204185)
Supplement: Supplementary Table 1, 4, and 6-8 [file aging-14-204185-s002.pdf]

SUPPLEMENTARY TABLES

Supplementary Table 1. Information for 20 active compounds of SWT.

Mol ID	Molecule Name	OB (%)	DL	Source
MOL000358	beta-sitosterol	36.91	0.75	*Radix Angelicae sinensis/ Radix Paeoniae Alba*
MOL000449	Stigmasterol	43.83	0.76	*Radix Angelicae sinensis/ Radix Rehmanniae Preparata*
MOL001494	Mandenol	42	0.19	*Rhizoma Chuanxiong*
MOL002135	Myricanone	40.6	0.51	*Rhizoma Chuanxiong*
MOL002140	Perlolyrine	65.95	0.27	*Rhizoma Chuanxiong*
MOL002151	senkyunone	47.66	0.24	*Rhizoma Chuanxiong*
MOL002157	wallichilide	42.31	0.71	*Rhizoma Chuanxiong*
MOL000433	FA	68.96	0.71	*Rhizoma Chuanxiong*
MOL000492	(+)-catechin	54.83	0.24	*Radix Paeoniae Alba*
MOL000359	3-epi-beta-Sitosterol	36.91	0.75	*Radix Paeoniae Alba/ Rhizoma Chuanxiong/ Radix Rehmanniae Preparata*
MOL001910	11alpha,12alpha-epoxy-3beta-23-dihydroxy-30-norolean-20-en-28,12beta-olide	64.77	0.38	*Radix Paeoniae Alba*
MOL001918	paeoniflorgenone	87.59	0.37	*Radix Paeoniae Alba*
MOL001919	Palbinone	43.56	0.53	*Radix Paeoniae Alba*
MOL001921	Lactiflorin	49.12	0.8	*Radix Paeoniae Alba*
MOL001924	paeoniflorin	53.87	0.79	*Radix Paeoniae Alba*
MOL001925	paeoniflorin_qt	68.18	0.4	*Radix Paeoniae Alba*
MOL001928	albiflorin_qt	66.64	0.33	*Radix Paeoniae Alba*
MOL001930	benzoyl paeoniflorin	31.27	0.75	*Radix Paeoniae Alba*
MOL000211	Mairin	55.38	0.78	*Radix Paeoniae Alba*
MOL000422	kaempferol	41.88	0.24	*Radix Paeoniae Alba*

Supplementary Table 4. The KEGG pathways enriched by 335 DEGs.

ID	Description	Class	Up	Down	*P* value
ko05200	Pathways in cancer	Human Diseases	19	8	5.17E-07
ko04210	Apoptosis	Cellular Processes	12	1	8.04E-07
ko05206	MicroRNAs in cancer	Human Diseases	10	4	1.64E-06
ko05222	Small cell lung cancer	Human Diseases	5	5	4.10E-06
ko05219	Bladder cancer	Human Diseases	7	0	5.99E-06
ko05224	Breast cancer	Human Diseases	8	4	1.33E-05
ko04010	MAPK signaling pathway	Environmental Information Processing	15	2	1.53E-05
ko04380	Osteoclast differentiation	Organismal Systems	9	2	1.56E-05
ko04068	FoxO signaling pathway	Environmental Information Processing	8	3	2.37E-05
ko04115	p53 signaling pathway	Cellular Processes	7	1	2.83E-05
ko05210	Colorectal cancer	Human Diseases	8	1	2.98E-05
ko04216	Ferroptosis	Cellular Processes	6	0	8.10E-05
ko05166	HTLV-I infection	Human Diseases	10	3	0.000153
ko05169	Epstein-Barr virus infection	Human Diseases	10	4	0.000315
ko05220	Chronic myeloid leukemia	Human Diseases	5	2	0.000381
ko05167	Kaposi sarcoma-associated herpesvirus infection	Human Diseases	9	2	0.000469
ko05225	Hepatocellular carcinoma	Human Diseases	6	4	0.0007
ko05203	Viral carcinogenesis	Human Diseases	6	5	0.000873
ko04064	NF-kappa B signaling pathway	Environmental Information Processing	8	2	0.000913
ko05226	Gastric cancer	Human Diseases	4	5	0.001193
ko05223	Non-small cell lung cancer	Human Diseases	4	2	0.001195
ko04110	Cell cycle	Cellular Processes	5	3	0.001596
ko01524	Platinum drug resistance	Human Diseases	5	1	0.001711
ko05218	Melanoma	Human Diseases	4	2	0.001832
ko04218	Cellular senescence	Cellular Processes	6	3	0.002103
ko05163	Human cytomegalovirus infection	Human Diseases	7	4	0.002168
ko05214	Glioma	Human Diseases	4	2	0.002234
ko04625	C-type lectin receptor signaling pathway	Organismal Systems	6	1	0.00243
ko05212	Pancreatic cancer	Human Diseases	4	2	0.002537
ko04915	Estrogen signaling pathway	Organismal Systems	5	3	0.002794
ko04668	TNF signaling pathway	Environmental Information Processing	5	2	0.003295
ko05205	Proteoglycans in cancer	Human Diseases	7	3	0.003347
ko05216	Thyroid cancer	Human Diseases	4	0	0.00384
ko04550	Signaling pathways regulating pluripotency of stem cells	Cellular Processes	3	5	0.004084
ko05213	Endometrial cancer	Human Diseases	4	1	0.004306
ko05217	Basal cell carcinoma	Human Diseases	3	2	0.004607
ko04060	Cytokine-cytokine receptor interaction	Environmental Information Processing	11	1	0.005443
ko04390	Hippo signaling pathway	Environmental Information Processing	4	4	0.005791
ko04932	Non-alcoholic fatty liver disease (NAFLD)	Human Diseases	7	1	0.006949
ko05161	Hepatitis B	Human Diseases	7	1	0.007723

ko04630	Jak-STAT signaling pathway	Environmental Information Processing	7	1	0.008855
ko05418	Fluid shear stress and atherosclerosis	Human Diseases	5	2	0.010745
ko05164	Influenza A	Human Diseases	6	2	0.011486
ko01521	EGFR tyrosine kinase inhibitor resistance	Human Diseases	3	2	0.014926
ko05202	Transcriptional misregulation in cancers	Human Diseases	9	1	0.016327
ko05134	Legionellosis	Human Diseases	4	0	0.01699
ko04012	ErbB signaling pathway	Environmental Information Processing	4	1	0.017122
ko04215	Apoptosis - multiple species	Cellular Processes	3	0	0.018319
ko04150	mTOR signaling pathway	Environmental Information Processing	3	4	0.021468
ko04657	IL-17 signaling pathway	Organismal Systems	4	1	0.024964
ko04061	Viral protein interaction with cytokine and cytokine receptor	Environmental Information Processing	5	0	0.029085
ko04933	AGE-RAGE signaling pathway in diabetic complications	Human Diseases	4	1	0.031298
ko01522	Endocrine resistance	Human Diseases	4	1	0.032443
ko05221	Acute myeloid leukemia	Human Diseases	3	1	0.034365
ko05165	Human papillomavirus infection	Human Diseases	5	6	0.034422
ko05230	Central carbon metabolism in cancer	Human Diseases	2	2	0.037457
ko04928	Parathyroid hormone synthesis, secretion and action	Organismal Systems	4	1	0.038559
ko04151	PI3K-Akt signaling pathway	Environmental Information Processing	9	4	0.040505
ko04660	T cell receptor signaling pathway	Organismal Systems	4	1	0.04119

Supplementary Table 6. The KEGG pathways enriched by 33 aging/senescence-induced DEGs.

ID	Description	Class	Ratio	*P* value
ko05200	Pathways in cancer	Human Diseases	0.022	2.67E-08
ko05167	Kaposi sarcoma-associated herpesvirus infection	Human Diseases	0.041	8.31E-08
ko05219	Bladder cancer	Human Diseases	0.119	1.57E-07
ko05206	MicroRNAs in cancer	Human Diseases	0.04	7.93E-07
ko01524	Platinum drug resistance	Human Diseases	0.067	2.97E-06
ko05166	HTLV-I infection	Human Diseases	0.03	4.90E-06
ko05163	Human cytomegalovirus infection	Human Diseases	0.03	5.34E-06
ko04010	MAPK signaling pathway	Environmental Information Processing	0.023	2.95E-05
ko04210	Apoptosis	Cellular Processes	0.035	6.68E-05
ko04115	p53 signaling pathway	Cellular Processes	0.056	6.70E-05
ko04932	Non-alcoholic fatty liver disease (NAFLD)	Human Diseases	0.03	0.000132
ko05202	Transcriptional misregulation in cancers	Human Diseases	0.022	0.000142
ko05161	Hepatitis B	Human Diseases	0.03	0.000144
ko04630	Jak-STAT signaling pathway	Environmental Information Processing	0.029	0.000161

ko05210	Colorectal cancer	Human Diseases	0.043	0.00019
ko04933	AGE-RAGE signaling pathway in diabetic complications	Human Diseases	0.039	0.00027
ko04216	Ferroptosis	Cellular Processes	0.07	0.000309
ko05205	Proteoglycans in cancer	Human Diseases	0.024	0.000437
ko04068	FoxO signaling pathway	Environmental Information Processing	0.029	0.000841
ko05224	Breast cancer	Human Diseases	0.026	0.001293
ko05221	Acute myeloid leukemia	Human Diseases	0.042	0.001404
ko05169	Epstein-Barr virus infection	Human Diseases	0.018	0.001588
ko04218	Cellular senescence	Cellular Processes	0.024	0.001627
ko05220	Chronic myeloid leukemia	Human Diseases	0.038	0.001835
ko05222	Small cell lung cancer	Human Diseases	0.032	0.003016
ko05203	Viral carcinogenesis	Human Diseases	0.019	0.00376
ko01522	Endocrine resistance	Human Diseases	0.029	0.004014
ko04928	Parathyroid hormone synthesis, secretion and action	Organismal Systems	0.028	0.004579
ko05142	Chagas disease (American trypanosomiasis)	Human Diseases	0.028	0.004579
ko04215	Apoptosis - multiple species	Cellular Processes	0.061	0.004681
ko05132	Salmonella infection	Human Diseases	0.018	0.004816
ko04668	TNF signaling pathway	Environmental Information Processing	0.026	0.005191
ko04066	HIF-1 signaling pathway	Environmental Information Processing	0.025	0.006128
ko05216	Thyroid cancer	Human Diseases	0.053	0.006172
ko04935	Growth hormone synthesis, secretion and action	Organismal Systems	0.024	0.006558
ko04110	Cell cycle	Cellular Processes	0.023	0.007315
ko04380	Osteoclast differentiation	Organismal Systems	0.023	0.007957
ko04120	Ubiquitin mediated proteolysis	Genetic Information Processing	0.021	0.010077
ko04930	Type II diabetes mellitus	Human Diseases	0.04	0.010511
ko04151	PI3K-Akt signaling pathway	Environmental Information Processing	0.011	0.011114
ko00270	Cysteine and methionine metabolism	Metabolism	0.038	0.011757
ko04060	Cytokine-cytokine receptor interaction	Environmental Information Processing	0.013	0.013578
ko05160	Hepatitis C	Human Diseases	0.018	0.013826
ko05134	Legionellosis	Human Diseases	0.034	0.013969
ko05323	Rheumatoid arthritis	Human Diseases	0.018	0.014753
ko00480	Glutathione metabolism	Metabolism	0.033	0.015376
ko05213	Endometrial cancer	Human Diseases	0.032	0.016347
ko04064	NF-kappa B signaling pathway	Environmental Information Processing	0.017	0.017484
ko04621	NOD-like receptor signaling pathway	Organismal Systems	0.016	0.019086
ko05165	Human papillomavirus infection	Human Diseases	0.012	0.021365

ko05211	Renal cell carcinoma	Human Diseases	0.027	0.022702
ko05218	Melanoma	Human Diseases	0.026	0.023271
ko04917	Prolactin signaling pathway	Organismal Systems	0.026	0.024427
ko05133	Pertussis	Human Diseases	0.026	0.024427
ko05214	Glioma	Human Diseases	0.025	0.025014
ko05212	Pancreatic cancer	Human Diseases	0.025	0.026205
ko01521	EGFR tyrosine kinase inhibitor resistance	Human Diseases	0.024	0.028655
ko04012	ErbB signaling pathway	Environmental Information Processing	0.023	0.030551
ko04350	TGF-beta signaling pathway	Environmental Information Processing	0.021	0.035844
ko04657	IL-17 signaling pathway	Organismal Systems	0.021	0.036529
ko04061	Viral protein interaction with cytokine and cytokine receptor	Environmental Information Processing	0.02	0.039321
ko05215	Prostate cancer	Human Diseases	0.019	0.040746
ko05131	Shigellosis	Human Diseases	0.012	0.042406
ko04620	Toll-like receptor signaling pathway	Organismal Systems	0.019	0.044396
ko04625	C-type lectin receptor signaling pathway	Organismal Systems	0.019	0.044396
ko04659	Th17 cell differentiation	Organismal Systems	0.018	0.048164
ko05145	Toxoplasmosis	Human Diseases	0.018	0.048164

Supplementary Table 7. The clinical characteristics of the BRCA patients in the TCGA datasets.

Characteristic	Levels	Overall
n		1083
T stage, *n* (%)	T1	277 (25.6%)
	T2	629 (58.2%)
	T3	139 (12.9%)
	T4	35 (3.2%)
N stage, *n* (%)	N0	514 (48.3%)
	N1	358 (33.6%)
	N2	116 (10.9%)
	N3	76 (7.1%)
M stage, *n* (%)	M0	902 (97.8%)
	M1	20 (2.2%)
Pathologic stage, *n* (%)	Stage I	181 (17.1%)
	Stage II	619 (58.4%)
	Stage III	242 (22.8%)
	Stage IV	18 (1.7%)
Race, *n* (%)	Asian	60 (6%)
	Black or African American	181 (18.2%)
	White	753 (75.8%)
Age, *n* (%)	<=60	601 (55.5%)
	>60	482 (44.5%)
Histological type, *n* (%)	Infiltrating Ductal Carcinoma	772 (79%)
	Infiltrating Lobular Carcinoma	205 (21%)
PR status, *n* (%)	Negative	342 (33.1%)
	Indeterminate	4 (0.4%)

	Positive	688 (66.5%)
ER status, *n* (%)	Negative	240 (23.2%)
	Indeterminate	2 (0.2%)
	Positive	793 (76.6%)
HER2 status, *n* (%)	Negative	558 (76.8%)
	Indeterminate	12 (1.7%)
	Positive	157 (21.6%)
Age, mean ± SD		58.25 ± 13.18

Supplementary Table 8. Univariate Cox proportional hazards regression analysis of 33 ASI-related DEGs.

Gene	HR (95% CI)	*P* value
NFKB2	0.787 (0.571–1.084)	0.142
CXCL8	1.032 (0.750–1.420)	0.846
GABARAPL1	1.010 (0.733–1.393)	0.950
INHBA	1.132 (0.822–1.557)	0.448
VEGFA	1.072 (0.779–1.475)	0.670
ISG20	0.754 (0.546–1.041)	0.086
CCN1	0.846 (0.615–1.163)	0.302
DDIT3	1.197 (0.870–1.646)	0.269
PMAIP1	0.848 (0.617–1.166)	0.310
ATF3	0.738 (0.537–1.016)	0.062
GCLC	1.192 (0.866–1.640)	0.282
NDRG1	1.410 (1.023–1.944)	0.036
EGR1	0.780 (0.566–1.073)	0.127
ERRFI1	0.716 (0.520–0.987)	0.041
IL6R	1.100 (0.799–1.515)	0.559
MYC	0.887 (0.645–1.221)	0.462
ETS2	1.014 (0.736–1.396)	0.933
PIM1	0.844 (0.612–1.162)	0.298
ZFP36	0.823 (0.598–1.132)	0.231
CDKN1A	1.200 (0.872–1.650)	0.262
MXD1	1.043 (0.757–1.439)	0.795
MAP1LC3B	1.093 (0.793–1.505)	0.588
SOCS1	0.723 (0.524–0.996)	0.047
FAS	0.903 (0.656–1.243)	0.532
ARG2	0.754 (0.546–1.040)	0.085
GCLM	1.100 (0.799–1.515)	0.560
IRS2	0.693 (0.504–0.953)	0.024
MDM2	1.074 (0.781–1.477)	0.660
FOS	0.801 (0.582–1.101)	0.172
BHLHE40	0.815 (0.592–1.121)	0.208
IGFBP4	0.706 (0.512–0.973)	0.033
DUSP6	0.917 (0.667–1.262)	0.595
BIRC3	0.670 (0.486–0.925)	0.015
