# Supplementary material for: Elucidating the pharmacological mechanism by which Si-Wu-Tang induces cellular senescence in breast cancer via multilevel data integration
Source: Aging (Albany NY). 2022 Jul 19;14(14):5812–37. doi: 10.18632/aging.204185 (PMC9365552; doi:10.18632/aging.204185)
Supplement: Supplementary Table 2-3 and 5 [file aging-14-204185-s003.docx]

**Supplementary Table 2. The DEGs between SWT-treated MCF-7 cell samples and DMSO control cell samples.**

| **Gene name** | **Log_2_FC** | ***P*.Value** | **adj.*P*.Val** | **Gene class** |
| --- | --- | --- | --- | --- |
| HSPA6 | 5.12355 | 1.82E-10 | 3.93E-07 | up |
| MAFF | 4.99707 | 5.67E-12 | 3.07E-08 | up |
| EGR4 | 4.362188 | 1.77E-12 | 2.93E-08 | up |
| ATF3 | 4.278374 | 4.06E-12 | 2.93E-08 | up |
| FOSL1 | 4.091685 | 3.99E-12 | 2.93E-08 | up |
| PMAIP1 | 3.512998 | 2.56E-11 | 1.11E-07 | up |
| EGR3 | 3.419405 | 3.63E-10 | 5.24E-07 | up |
| LINC00304 | 3.356435 | 1.39E-08 | 4.00E-06 | up |
| FOS | 3.280632 | 1.48E-10 | 3.62E-07 | up |
| LOC344887 | 3.269066 | 1.47E-10 | 3.62E-07 | up |
| HMOX1 | 3.061976 | 2.66E-10 | 4.60E-07 | up |
| FOSB | 3.002462 | 5.52E-10 | 6.65E-07 | up |
| CHD2 | 2.944979 | 2.97E-10 | 4.60E-07 | up |
| TIPARP | 2.928597 | 1.11E-10 | 3.62E-07 | up |
| DNAJB4 | 2.754647 | 2.90E-10 | 4.60E-07 | up |
| DUSP5 | 2.741974 | 3.93E-09 | 2.03E-06 | up |
| PPP1R15A | 2.739137 | 1.16E-09 | 9.93E-07 | up |
| ARL14 | 2.707833 | 9.96E-10 | 9.79E-07 | up |
| C14orf182 | 2.652812 | 1.04E-09 | 9.79E-07 | up |
| OSGIN1 | 2.641375 | 2.56E-10 | 4.60E-07 | up |
| RBM24 | 2.602805 | 3.18E-06 | 0.000141 | up |
| GREB1 | 2.345873 | 8.40E-09 | 3.19E-06 | up |
| GADD45A | 2.30976 | 1.50E-10 | 3.62E-07 | up |
| ANKRD37 | 2.287492 | 2.46E-09 | 1.52E-06 | up |
| STC2 | 2.24923 | 6.62E-10 | 7.54E-07 | up |
| SLC7A11 | 2.23004 | 5.39E-10 | 6.65E-07 | up |
| CXCL8 | 2.189773 | 2.00E-06 | 0.000105 | up |
| HBEGF | 2.17942 | 4.48E-09 | 2.20E-06 | up |
| RGCC | 2.169081 | 1.80E-09 | 1.20E-06 | up |
| GEM | 2.160745 | 2.63E-09 | 1.57E-06 | up |
| HSPB8 | 2.151666 | 7.78E-10 | 8.03E-07 | up |
| DDIT3 | 2.134307 | 1.84E-09 | 1.20E-06 | up |
| LIF | 2.112901 | 7.44E-08 | 1.25E-05 | up |
| NBPF8 | 2.104356 | 9.59E-09 | 3.52E-06 | up |
| SRXN1 | 2.08831 | 2.68E-09 | 1.57E-06 | up |
| MOB3C | 2.084781 | 1.23E-09 | 9.93E-07 | up |
| EGR1 | 2.054217 | 4.88E-09 | 2.35E-06 | up |
| IL6R | 1.99944 | 4.76E-08 | 9.54E-06 | up |
| TMPRSS3 | 1.966875 | 8.68E-08 | 1.36E-05 | up |
| RP11-489E7.4 | 1.944569 | 1.23E-08 | 3.76E-06 | up |
| MYC | 1.943457 | 3.11E-08 | 7.40E-06 | up |
| RGS2 | 1.895404 | 3.94E-09 | 2.03E-06 | up |
| EGR2 | 1.885811 | 7.05E-07 | 5.42E-05 | up |
| FBXO30 | 1.864614 | 7.64E-10 | 8.03E-07 | up |
| FAS | 1.832413 | 6.82E-09 | 2.84E-06 | up |
| GDF15 | 1.806988 | 1.69E-09 | 1.20E-06 | up |
| TNFRSF10A | 1.782453 | 1.40E-09 | 1.05E-06 | up |
| DEDD2 | 1.776241 | 1.41E-09 | 1.05E-06 | up |
| IRS2 | 1.775218 | 4.09E-09 | 2.06E-06 | up |
| NDRG1 | 1.75563 | 6.81E-09 | 2.84E-06 | up |
| RP11-21L23.2 | 1.750745 | 7.21E-09 | 2.89E-06 | up |
| LHX4 | 1.740037 | 6.78E-09 | 2.84E-06 | up |
| SERTAD1 | 1.730222 | 1.24E-09 | 9.93E-07 | up |
| TNFSF9 | 1.724256 | 1.01E-07 | 1.46E-05 | up |
| ERRFI1 | 1.723743 | 3.09E-09 | 1.67E-06 | up |
| SLC7A5 | 1.708607 | 1.21E-08 | 3.76E-06 | up |
| AGPAT9 | 1.705192 | 1.19E-08 | 3.76E-06 | up |
| F2RL1 | 1.697483 | 2.58E-08 | 6.36E-06 | up |
| ACRC | 1.695777 | 3.73E-08 | 8.33E-06 | up |
| ZFAND2A | 1.687869 | 2.85E-09 | 1.62E-06 | up |
| FOSL2 | 1.670628 | 1.06E-08 | 3.66E-06 | up |
| MAFG | 1.658105 | 1.98E-09 | 1.26E-06 | up |
| PCF11 | 1.656623 | 5.00E-09 | 2.35E-06 | up |
| ZBTB21 | 1.6479 | 2.84E-08 | 6.91E-06 | up |
| GCLM | 1.644876 | 6.21E-09 | 2.80E-06 | up |
| LRRC15 | 1.641965 | 9.52E-07 | 6.59E-05 | up |
| GCLC | 1.640683 | 1.75E-09 | 1.20E-06 | up |
| PLAUR | 1.637262 | 7.40E-08 | 1.25E-05 | up |
| CBR3 | 1.628631 | 6.94E-09 | 2.84E-06 | up |
| AREG | 1.625606 | 1.25E-08 | 3.76E-06 | up |
| ARC | 1.612243 | 8.50E-07 | 6.14E-05 | up |
| AEN | 1.611437 | 1.55E-08 | 4.41E-06 | up |
| NHLH2 | 1.609116 | 2.87E-06 | 0.00013 | up |
| SNHG17 | 1.567199 | 1.25E-08 | 3.76E-06 | up |
| ETS2 | 1.562951 | 3.36E-08 | 7.67E-06 | up |
| INHBA | 1.554859 | 3.28E-07 | 3.17E-05 | up |
| FOXC1 | 1.548633 | 8.56E-09 | 3.19E-06 | up |
| ARG2 | 1.545434 | 4.28E-08 | 8.92E-06 | up |
| BHLHE40 | 1.530089 | 1.06E-08 | 3.66E-06 | up |
| CYR61 | 1.527131 | 3.79E-08 | 8.38E-06 | up |
| HEY2 | 1.525418 | 1.04E-06 | 6.93E-05 | up |
| JMJD6 | 1.520664 | 2.35E-08 | 5.99E-06 | up |
| PITHD1 | 1.518578 | 2.96E-09 | 1.64E-06 | up |
| PGR | 1.502005 | 7.68E-08 | 1.26E-05 | up |
| C3orf52 | 1.50002 | 1.05E-08 | 3.66E-06 | up |
| NPY1R | 1.491484 | 1.44E-07 | 1.95E-05 | up |
| NR4A3 | 1.48971 | 9.17E-08 | 1.39E-05 | up |
| STK17A | 1.489614 | 8.38E-09 | 3.19E-06 | up |
| MXD1 | 1.47442 | 9.85E-09 | 3.55E-06 | up |
| TXNRD1 | 1.470163 | 1.10E-08 | 3.66E-06 | up |
| MAP1B | 1.469979 | 8.73E-07 | 6.24E-05 | up |
| CCRN4L | 1.46984 | 3.35E-08 | 7.67E-06 | up |
| STYK1 | 1.466741 | 1.96E-08 | 5.21E-06 | up |
| ZNF654 | 1.459442 | 5.27E-08 | 1.04E-05 | up |
| C11orf96 | 1.448893 | 6.33E-08 | 1.15E-05 | up |
| UNKL | 1.427113 | 1.91E-08 | 5.21E-06 | up |
| CSRNP1 | 1.422952 | 1.59E-08 | 4.42E-06 | up |
| IER5 | 1.406254 | 1.96E-08 | 5.21E-06 | up |
| TNFRSF10B | 1.404556 | 1.22E-08 | 3.76E-06 | up |
| RP11-373D23.2 | 1.400921 | 0.000151 | 0.00176 | up |
| RND1 | 1.397529 | 5.68E-09 | 2.62E-06 | up |
| PIM1 | 1.394734 | 6.72E-08 | 1.19E-05 | up |
| GADD45B | 1.391501 | 1.19E-08 | 3.76E-06 | up |
| MDM2 | 1.384877 | 2.00E-08 | 5.21E-06 | up |
| SLC3A2 | 1.377311 | 1.57E-08 | 4.41E-06 | up |
| CALCR | 1.37033 | 6.68E-07 | 5.24E-05 | up |
| KCNE4 | 1.367274 | 3.00E-05 | 0.000588 | up |
| NUPL1 | 1.363857 | 2.90E-08 | 6.97E-06 | up |
| ZNF547 | 1.356586 | 3.67E-06 | 0.00015 | up |
| IL20RB | 1.353351 | 2.27E-07 | 2.52E-05 | up |
| AGR3 | 1.351505 | 7.68E-07 | 5.67E-05 | up |
| SLC25A25 | 1.350023 | 4.52E-08 | 9.23E-06 | up |
| SESN2 | 1.331526 | 2.45E-08 | 6.09E-06 | up |
| AGO2 | 1.323861 | 6.91E-07 | 5.36E-05 | up |
| VEGFA | 1.316338 | 1.71E-07 | 2.16E-05 | up |
| EID3 | 1.31146 | 4.01E-06 | 0.00016 | up |
| NUP98 | 1.309125 | 3.51E-07 | 3.30E-05 | up |
| KLF2 | 1.30301 | 1.72E-06 | 9.63E-05 | up |
| GPATCH2L | 1.300567 | 1.69E-07 | 2.15E-05 | up |
| CSRP2 | 1.300541 | 2.37E-07 | 2.58E-05 | up |
| CREG2 | 1.296056 | 4.05E-07 | 3.67E-05 | up |
| EIF3C | 1.293879 | 6.79E-05 | 0.000995 | up |
| UBALD2 | 1.292986 | 2.39E-08 | 6.01E-06 | up |
| KLF10 | 1.292762 | 4.71E-08 | 9.53E-06 | up |
| DNAJA4 | 1.29262 | 3.82E-06 | 0.000155 | up |
| CLK1 | 1.290756 | 1.38E-07 | 1.91E-05 | up |
| RP11-271C24.3 | 1.289893 | 6.02E-05 | 0.000912 | up |
| ZNF473 | 1.285821 | 9.56E-08 | 1.44E-05 | up |
| LINC00115 | 1.285442 | 9.72E-08 | 1.44E-05 | up |
| RIT1 | 1.285355 | 1.04E-07 | 1.49E-05 | up |
| LSMEM1 | 1.282831 | 3.13E-06 | 0.000139 | up |
| CTA-29F11.1 | 1.282706 | 1.26E-06 | 7.76E-05 | up |
| IRAK2 | 1.274406 | 4.28E-05 | 0.000729 | up |
| MAP3K14 | 1.272869 | 8.91E-08 | 1.37E-05 | up |
| HIST1H2AK | 1.270844 | 2.40E-06 | 0.000117 | up |
| E2F7 | 1.269371 | 1.32E-08 | 3.87E-06 | up |
| C6orf141 | 1.261442 | 1.04E-05 | 0.000289 | up |
| SOCS1 | 1.25732 | 4.34E-08 | 8.94E-06 | up |
| KLHL21 | 1.251907 | 4.16E-08 | 8.76E-06 | up |
| BD495725 | 1.250219 | 2.25E-05 | 0.000484 | up |
| MYB | 1.245067 | 3.46E-07 | 3.28E-05 | up |
| RPS16P5 | 1.245033 | 0.000501 | 0.004172 | up |
| ADM | 1.238999 | 3.27E-07 | 3.17E-05 | up |
| BIRC3 | 1.238474 | 1.05E-05 | 0.000291 | up |
| SERTAD2 | 1.237475 | 2.53E-06 | 0.000121 | up |
| ICOSLG | 1.235752 | 1.47E-07 | 1.96E-05 | up |
| CHAC1 | 1.235112 | 8.84E-07 | 6.25E-05 | up |
| RAMP3 | 1.232479 | 1.60E-06 | 9.17E-05 | up |
| ADORA2B | 1.227696 | 7.65E-08 | 1.26E-05 | up |
| GABARAPL1 | 1.226968 | 6.09E-07 | 4.95E-05 | up |
| RELT | 1.220315 | 6.77E-08 | 1.19E-05 | up |
| FHL2 | 1.219317 | 2.32E-06 | 0.000116 | up |
| CABYR | 1.219032 | 2.53E-07 | 2.70E-05 | up |
| PHLDA1 | 1.218862 | 3.37E-08 | 7.67E-06 | up |
| CIART | 1.218283 | 3.99E-07 | 3.63E-05 | up |
| OSER1 | 1.216967 | 4.97E-08 | 9.88E-06 | up |
| NFKB2 | 1.210982 | 0.000522 | 0.0043 | up |
| RP11-391M1.4 | 1.206213 | 1.43E-07 | 1.95E-05 | up |
| ZBTB43 | 1.204538 | 3.09E-07 | 3.06E-05 | up |
| IFRD1 | 1.198185 | 4.32E-07 | 3.83E-05 | up |
| ZNF674-AS1 | 1.198023 | 1.69E-05 | 0.000401 | up |
| ITPKC | 1.192106 | 1.98E-08 | 5.21E-06 | up |
| KDM3A | 1.187449 | 2.14E-07 | 2.42E-05 | up |
| C10orf2 | 1.18379 | 1.32E-07 | 1.85E-05 | up |
| FOXQ1 | 1.182873 | 0.000235 | 0.002423 | up |
| DNMBP | 1.17677 | 5.66E-08 | 1.05E-05 | up |
| DUSP1 | 1.176248 | 2.06E-08 | 5.32E-06 | up |
| DNAJB1 | 1.174653 | 6.52E-08 | 1.17E-05 | up |
| OVOL1 | 1.172751 | 9.04E-08 | 1.38E-05 | up |
| CPEB4 | 1.164879 | 2.59E-06 | 0.000122 | up |
| PTGR1 | 1.160729 | 1.97E-07 | 2.33E-05 | up |
| SLC25A33 | 1.154021 | 1.74E-07 | 2.17E-05 | up |
| MICB | 1.152385 | 2.25E-07 | 2.51E-05 | up |
| SLC47A1 | 1.150479 | 5.43E-05 | 0.000857 | up |
| LIPG | 1.150309 | 2.39E-06 | 0.000117 | up |
| KRT13 | 1.146189 | 2.86E-05 | 0.000568 | up |
| RIPK4 | 1.139989 | 1.48E-07 | 1.97E-05 | up |
| ASNS | 1.137034 | 3.98E-08 | 8.61E-06 | up |
| LOC399900 | 1.136125 | 0.005175 | 0.023737 | up |
| CDKN1A | 1.13387 | 9.82E-08 | 1.45E-05 | up |
| MIR3682 | 1.132554 | 4.02E-06 | 0.00016 | up |
| LONRF2 | 1.130024 | 8.25E-08 | 1.33E-05 | up |
| BRE-AS1 | 1.124089 | 7.14E-06 | 0.000229 | up |
| CARD10 | 1.123983 | 2.38E-06 | 0.000117 | up |
| DNTTIP2 | 1.118288 | 7.67E-08 | 1.26E-05 | up |
| NCOA7 | 1.11746 | 2.72E-07 | 2.83E-05 | up |
| PPRC1 | 1.116831 | 5.59E-08 | 1.05E-05 | up |
| NOP16 | 1.105355 | 2.98E-07 | 2.99E-05 | up |
| BCL10 | 1.105201 | 6.95E-08 | 1.19E-05 | up |
| C1orf54 | 1.105025 | 2.45E-07 | 2.63E-05 | up |
| E2F6 | 1.101077 | 5.43E-08 | 1.05E-05 | up |
| BCL2L11 | 1.099917 | 3.89E-08 | 8.50E-06 | up |
| PPIF | 1.095214 | 5.49E-08 | 1.05E-05 | up |
| THAP9-AS1 | 1.094673 | 3.78E-07 | 3.51E-05 | up |
| MAFK | 1.093669 | 5.05E-07 | 4.25E-05 | up |
| FAM53C | 1.091063 | 9.85E-07 | 6.70E-05 | up |
| PKIB | 1.088984 | 2.35E-07 | 2.58E-05 | up |
| MGC20647 | 1.083353 | 9.70E-06 | 0.000277 | up |
| EPHA2 | 1.081565 | 8.83E-08 | 1.37E-05 | up |
| MIR5188 | 1.066692 | 1.77E-06 | 9.72E-05 | up |
| ERN1 | 1.066 | 4.19E-07 | 3.75E-05 | up |
| MC4R | 1.064301 | 2.18E-05 | 0.000474 | up |
| RP1-30M3.5 | 1.062046 | 1.66E-07 | 2.14E-05 | up |
| CCDC150 | 1.061454 | 3.60E-05 | 0.000655 | up |
| THUMPD3-AS1 | 1.061003 | 0.000265 | 0.002635 | up |
| KLF6 | 1.060392 | 1.01E-07 | 1.46E-05 | up |
| SNORA72 | 1.058672 | 6.72E-05 | 0.000987 | up |
| MYADM | 1.058575 | 5.67E-06 | 0.000192 | up |
| RP1-151F17.2 | 1.057193 | 1.25E-07 | 1.77E-05 | up |
| MAP1LC3B | 1.056506 | 7.06E-08 | 1.20E-05 | up |
| PHLDA3 | 1.054985 | 3.01E-07 | 3.01E-05 | up |
| NFKBIE | 1.053377 | 1.38E-06 | 8.39E-05 | up |
| EPT1 | 1.045436 | 5.41E-06 | 0.000186 | up |
| ISG20 | 1.043888 | 1.30E-07 | 1.83E-05 | up |
| ZFP36 | 1.041409 | 1.44E-07 | 1.95E-05 | up |
| ZCCHC2 | 1.035534 | 2.85E-07 | 2.90E-05 | up |
| DUSP6 | 1.031702 | 5.29E-06 | 0.000184 | up |
| YRDC | 1.029794 | 1.91E-07 | 2.30E-05 | up |
| C9orf72 | 1.029091 | 1.40E-05 | 0.000351 | up |
| AKIRIN2 | 1.025695 | 1.82E-06 | 9.87E-05 | up |
| CEBPG | 1.02448 | 2.10E-07 | 2.42E-05 | up |
| LYAR | 1.022079 | 9.67E-08 | 1.44E-05 | up |
| KCTD9 | 1.015957 | 1.67E-07 | 2.15E-05 | up |
| DAPP1 | 1.013755 | 7.65E-07 | 5.67E-05 | up |
| BMS1P20 | 1.013015 | 6.89E-06 | 0.000223 | up |
| PLAC1 | 1.013005 | 5.43E-06 | 0.000187 | up |
| IGFBP4 | 1.012797 | 1.95E-05 | 0.000439 | up |
| RASSF1 | 1.01104 | 3.48E-07 | 3.29E-05 | up |
| SERPINB8 | 1.010932 | 1.01E-05 | 0.000284 | up |
| CCNL1 | 1.007051 | 1.18E-06 | 7.51E-05 | up |
| HS3ST3A1 | 1.006612 | 1.94E-06 | 0.000103 | up |
| DUSP14 | 1.003699 | 2.23E-07 | 2.51E-05 | up |
| TRIB1 | 1.002963 | 1.76E-07 | 2.18E-05 | up |
| AKR1B10 | 1.001989 | 5.26E-06 | 0.000184 | up |
| TRAF5 | -1.0001 | 1.92E-07 | 2.30E-05 | down |
| NHS | -1.00214 | 1.16E-05 | 0.000315 | down |
| DNM3 | -1.00297 | 3.11E-05 | 0.000601 | down |
| B3GNT1 | -1.00988 | 3.88E-07 | 3.58E-05 | down |
| RWDD2A | -1.01038 | 2.19E-06 | 0.000111 | down |
| RP11-250B2.6 | -1.01271 | 0.002827 | 0.015207 | down |
| ARHGAP18 | -1.01287 | 1.39E-06 | 8.39E-05 | down |
| TMTC2 | -1.01396 | 1.19E-05 | 0.000319 | down |
| DTX4 | -1.01608 | 6.02E-06 | 0.0002 | down |
| GLCE | -1.01989 | 7.88E-06 | 0.000245 | down |
| DBP | -1.02611 | 6.11E-05 | 0.000922 | down |
| PARP9 | -1.02748 | 1.36E-05 | 0.000346 | down |
| PGM2L1 | -1.03346 | 3.06E-06 | 0.000137 | down |
| KALRN | -1.03946 | 4.49E-07 | 3.97E-05 | down |
| NAPEPLD | -1.04044 | 1.72E-06 | 9.63E-05 | down |
| SALL4 | -1.05792 | 5.43E-07 | 4.50E-05 | down |
| CORO2A | -1.06727 | 6.16E-07 | 4.99E-05 | down |
| PIK3C2B | -1.06809 | 7.45E-07 | 5.60E-05 | down |
| BCL11B | -1.0692 | 4.46E-06 | 0.000171 | down |
| DOCK11 | -1.06947 | 7.39E-05 | 0.001056 | down |
| SYNPO2 | -1.07356 | 0.000164 | 0.001868 | down |
| NBEA | -1.07771 | 5.72E-07 | 4.71E-05 | down |
| ITGB8 | -1.07991 | 1.39E-06 | 8.39E-05 | down |
| SKP2 | -1.08014 | 9.97E-08 | 1.46E-05 | down |
| TBC1D32 | -1.08069 | 9.52E-05 | 0.001255 | down |
| KBTBD3 | -1.08271 | 0.000116 | 0.001446 | down |
| C9orf152 | -1.08702 | 2.65E-07 | 2.78E-05 | down |
| LINC00263 | -1.09384 | 5.87E-05 | 0.0009 | down |
| SLC8A1 | -1.09474 | 2.52E-05 | 0.000525 | down |
| FBN2 | -1.0987 | 5.59E-07 | 4.62E-05 | down |
| FZD4 | -1.09955 | 2.38E-07 | 2.58E-05 | down |
| GATA2 | -1.10289 | 5.63E-08 | 1.05E-05 | down |
| RAB27B | -1.10738 | 8.30E-08 | 1.33E-05 | down |
| GLDN | -1.10787 | 6.84E-08 | 1.19E-05 | down |
| FRMD4A | -1.12532 | 8.40E-06 | 0.000255 | down |
| LFNG | -1.12543 | 8.47E-08 | 1.35E-05 | down |
| GABBR2 | -1.12911 | 2.02E-06 | 0.000105 | down |
| AIM1 | -1.13032 | 1.74E-07 | 2.17E-05 | down |
| SAMD13 | -1.13581 | 2.81E-06 | 0.000128 | down |
| TMOD2 | -1.14059 | 1.74E-07 | 2.17E-05 | down |
| ZNF704 | -1.14759 | 4.47E-06 | 0.000171 | down |
| GALNT12 | -1.15355 | 3.32E-08 | 7.67E-06 | down |
| PVRL3 | -1.15374 | 4.40E-05 | 0.00074 | down |
| SLITRK1 | -1.16786 | 1.33E-05 | 0.000341 | down |
| FGFR2 | -1.18165 | 7.21E-07 | 5.50E-05 | down |
| SUOX | -1.1915 | 1.21E-06 | 7.58E-05 | down |
| MROH2A | -1.20169 | 5.31E-06 | 0.000184 | down |
| APCDD1 | -1.21221 | 7.87E-06 | 0.000245 | down |
| SKIDA1 | -1.21428 | 2.14E-07 | 2.42E-05 | down |
| MARCKS | -1.21742 | 4.69E-07 | 4.08E-05 | down |
| PRR15L | -1.22355 | 3.43E-08 | 7.74E-06 | down |
| SNAI2 | -1.22692 | 3.42E-07 | 3.28E-05 | down |
| IRAK1BP1 | -1.23019 | 5.84E-06 | 0.000196 | down |
| LOC101927391 | -1.23136 | 5.40E-05 | 0.000853 | down |
| ANKRD50 | -1.23823 | 1.62E-07 | 2.11E-05 | down |
| AQP11 | -1.23965 | 2.38E-06 | 0.000117 | down |
| IGDCC3 | -1.24349 | 1.96E-06 | 0.000104 | down |
| HOTAIR | -1.25606 | 3.68E-06 | 0.000151 | down |
| NRK | -1.25981 | 2.43E-07 | 2.62E-05 | down |
| TNS3 | -1.26572 | 7.72E-08 | 1.26E-05 | down |
| LMO3 | -1.2708 | 1.56E-07 | 2.05E-05 | down |
| RNF144B | -1.27304 | 3.97E-07 | 3.63E-05 | down |
| TET1 | -1.28363 | 6.35E-08 | 1.15E-05 | down |
| CDK6 | -1.29478 | 1.16E-07 | 1.65E-05 | down |
| NCKAP5 | -1.29828 | 2.74E-05 | 0.000554 | down |
| RNF43 | -1.30364 | 1.31E-08 | 3.87E-06 | down |
| MKX | -1.30776 | 0.000355 | 0.003232 | down |
| SOX2 | -1.30852 | 4.12E-08 | 8.76E-06 | down |
| IL1R1 | -1.30869 | 5.57E-08 | 1.05E-05 | down |
| KAL1 | -1.32041 | 1.40E-05 | 0.000351 | down |
| DOCK4 | -1.32547 | 1.77E-07 | 2.18E-05 | down |
| PIK3R3 | -1.34064 | 1.08E-08 | 3.66E-06 | down |
| PDE5A | -1.35677 | 1.62E-07 | 2.11E-05 | down |
| EPHA3 | -1.38833 | 1.02E-05 | 0.000287 | down |
| IFIT1 | -1.39282 | 3.34E-05 | 0.000632 | down |
| NPAS3 | -1.39821 | 6.82E-08 | 1.19E-05 | down |
| CDKN2B | -1.40905 | 8.82E-08 | 1.37E-05 | down |
| LOC440934 | -1.4126 | 9.64E-06 | 0.000277 | down |
| FILIP1L | -1.42116 | 2.79E-07 | 2.85E-05 | down |
| ZNF792 | -1.42557 | 5.86E-07 | 4.81E-05 | down |
| LOC102724387 | -1.44445 | 7.75E-07 | 5.69E-05 | down |
| LOC100507516 | -1.48392 | 7.02E-07 | 5.42E-05 | down |
| PCDH7 | -1.5096 | 6.44E-09 | 2.84E-06 | down |
| SLCO4C1 | -1.51644 | 0.001257 | 0.008352 | down |
| FUT9 | -1.51666 | 7.34E-09 | 2.89E-06 | down |
| VAT1L | -1.52762 | 8.94E-07 | 6.30E-05 | down |
| ST8SIA4 | -1.52835 | 4.09E-07 | 3.68E-05 | down |
| KCNJ8 | -1.5321 | 3.37E-06 | 0.000144 | down |
| TTC30B | -1.55388 | 9.00E-07 | 6.33E-05 | down |
| EPHA7 | -1.55456 | 8.60E-08 | 1.36E-05 | down |
| SPRY1 | -1.56834 | 3.24E-06 | 0.000141 | down |
| KCNJ3 | -1.59719 | 5.52E-08 | 1.05E-05 | down |
| MMP16 | -1.62303 | 5.79E-08 | 1.06E-05 | down |
| DIO2 | -1.62366 | 3.60E-05 | 0.000655 | down |
| PCDH10 | -1.68454 | 1.45E-07 | 1.96E-05 | down |
| SEMA3D | -1.73956 | 8.89E-06 | 0.000262 | down |
| FZD2 | -1.78539 | 2.00E-07 | 2.35E-05 | down |
| STON1 | -1.90149 | 4.02E-08 | 8.62E-06 | down |
| PPP1R3C | -1.95376 | 1.12E-09 | 9.93E-07 | down |
| TRERF1 | -1.99645 | 2.14E-07 | 2.42E-05 | down |
| IKZF2 | -2.12411 | 5.16E-10 | 6.65E-07 | down |

**Supplementary Table 3. The GO terms enriched by 335 DEGs (*P* < 0.05).**

| **ID** | **Description** | **Class** | **Up** | **Down** | ***P* value** |
| --- | --- | --- | --- | --- | --- |
| GO:0048522 | positive regulation of cellular process | Biological Process | 109 | 42 | 3.96E-17 |
| GO:0031325 | positive regulation of cellular metabolic process | Biological Process | 91 | 20 | 4.74E-17 |
| GO:0048518 | positive regulation of biological process | Biological Process | 117 | 45 | 3.79E-16 |
| GO:0009893 | positive regulation of metabolic process | Biological Process | 93 | 22 | 4.72E-16 |
| GO:0010604 | positive regulation of macromolecule metabolic process | Biological Process | 88 | 20 | 1.09E-15 |
| GO:0048519 | negative regulation of biological process | Biological Process | 115 | 34 | 1.92E-15 |
| GO:0051173 | positive regulation of nitrogen compound metabolic process | Biological Process | 85 | 19 | 2.36E-15 |
| GO:0031323 | regulation of cellular metabolic process | Biological Process | 126 | 33 | 7.23E-15 |
| GO:0019222 | regulation of metabolic process | Biological Process | 131 | 35 | 8.49E-14 |
| GO:0060255 | regulation of macromolecule metabolic process | Biological Process | 122 | 34 | 1.55E-13 |
| GO:0080090 | regulation of primary metabolic process | Biological Process | 121 | 32 | 3.62E-13 |
| GO:0051171 | regulation of nitrogen compound metabolic process | Biological Process | 119 | 31 | 3.94E-13 |
| GO:0010941 | regulation of cell death | Biological Process | 55 | 11 | 5.66E-13 |
| GO:0051254 | positive regulation of RNA metabolic process | Biological Process | 53 | 13 | 1.05E-12 |
| GO:0010628 | positive regulation of gene expression | Biological Process | 59 | 16 | 1.22E-12 |
| GO:0008219 | cell death | Biological Process | 66 | 11 | 1.67E-12 |
| GO:0045944 | positive regulation of transcription by RNA polymerase II | Biological Process | 44 | 9 | 1.78E-12 |
| GO:0045893 | positive regulation of transcription, DNA-templated | Biological Process | 50 | 13 | 2.70E-12 |
| GO:1903508 | positive regulation of nucleic acid-templated transcription | Biological Process | 50 | 13 | 2.70E-12 |
| GO:1902680 | positive regulation of RNA biosynthetic process | Biological Process | 50 | 13 | 2.78E-12 |
| GO:0001228 | DNA-binding transcription activator activity, RNA polymerase II-specific | Molecular Function | 26 | 4 | 7.35E-12 |
| GO:0042981 | regulation of apoptotic process | Biological Process | 49 | 11 | 7.61E-12 |
| GO:0006915 | apoptotic process | Biological Process | 58 | 11 | 9.94E-12 |
| GO:0048523 | negative regulation of cellular process | Biological Process | 97 | 30 | 1.01E-11 |
| GO:0010557 | positive regulation of macromolecule biosynthetic process | Biological Process | 54 | 13 | 1.22E-11 |
| GO:0043067 | regulation of programmed cell death | Biological Process | 49 | 11 | 1.56E-11 |
| GO:0042325 | regulation of phosphorylation | Biological Process | 47 | 14 | 2.19E-11 |
| GO:0031328 | positive regulation of cellular biosynthetic process | Biological Process | 56 | 13 | 2.25E-11 |
| GO:0009889 | regulation of biosynthetic process | Biological Process | 93 | 22 | 2.72E-11 |
| GO:0009891 | positive regulation of biosynthetic process | Biological Process | 56 | 13 | 4.33E-11 |
| GO:0031399 | regulation of protein modification process | Biological Process | 50 | 16 | 4.36E-11 |
| GO:0050794 | regulation of cellular process | Biological Process | 155 | 63 | 4.74E-11 |
| GO:0045935 | positive regulation of nucleobase-containing compound metabolic process | Biological Process | 54 | 13 | 5.59E-11 |
| GO:0033554 | cellular response to stress | Biological Process | 61 | 10 | 6.25E-11 |
| GO:0043549 | regulation of kinase activity | Biological Process | 32 | 10 | 8.14E-11 |
| GO:0065009 | regulation of molecular function | Biological Process | 71 | 20 | 9.15E-11 |
| GO:0012501 | programmed cell death | Biological Process | 59 | 11 | 9.31E-11 |
| GO:0032268 | regulation of cellular protein metabolic process | Biological Process | 63 | 19 | 1.04E-10 |
| GO:0007275 | multicellular organism development | Biological Process | 94 | 40 | 1.41E-10 |
| GO:0010033 | response to organic substance | Biological Process | 73 | 21 | 1.73E-10 |
| GO:0001932 | regulation of protein phosphorylation | Biological Process | 42 | 13 | 2.07E-10 |
| GO:2000112 | regulation of cellular macromolecule biosynthetic process | Biological Process | 85 | 22 | 2.47E-10 |
| GO:0051338 | regulation of transferase activity | Biological Process | 34 | 10 | 2.98E-10 |
| GO:0031326 | regulation of cellular biosynthetic process | Biological Process | 89 | 22 | 3.25E-10 |
| GO:0009991 | response to extracellular stimulus | Biological Process | 27 | 5 | 3.37E-10 |
| GO:0048856 | anatomical structure development | Biological Process | 100 | 41 | 3.84E-10 |
| GO:0051246 | regulation of protein metabolic process | Biological Process | 66 | 19 | 4.05E-10 |
| GO:0010556 | regulation of macromolecule biosynthetic process | Biological Process | 85 | 22 | 4.12E-10 |
| GO:0009888 | tissue development | Biological Process | 49 | 19 | 4.30E-10 |
| GO:0006357 | regulation of transcription by RNA polymerase II | Biological Process | 64 | 16 | 4.85E-10 |
| GO:0008283 | cell proliferation | Biological Process | 50 | 15 | 5.14E-10 |
| GO:0035556 | intracellular signal transduction | Biological Process | 65 | 20 | 6.15E-10 |
| GO:0048585 | negative regulation of response to stimulus | Biological Process | 50 | 13 | 6.24E-10 |
| GO:0032502 | developmental process | Biological Process | 103 | 44 | 6.37E-10 |
| GO:0071496 | cellular response to external stimulus | Biological Process | 21 | 4 | 7.50E-10 |
| GO:0006468 | protein phosphorylation | Biological Process | 50 | 16 | 9.17E-10 |
| GO:0019220 | regulation of phosphate metabolic process | Biological Process | 48 | 14 | 9.58E-10 |
| GO:0051174 | regulation of phosphorus metabolic process | Biological Process | 48 | 14 | 9.58E-10 |
| GO:0006950 | response to stress | Biological Process | 91 | 19 | 1.16E-09 |
| GO:0000987 | proximal promoter sequence-specific DNA binding | Molecular Function | 37 | 10 | 1.31E-09 |
| GO:0044260 | cellular macromolecule metabolic process | Biological Process | 134 | 44 | 1.84E-09 |
| GO:0010468 | regulation of gene expression | Biological Process | 92 | 25 | 2.10E-09 |
| GO:0006355 | regulation of transcription, DNA-templated | Biological Process | 76 | 20 | 2.16E-09 |
| GO:1903506 | regulation of nucleic acid-templated transcription | Biological Process | 76 | 20 | 2.23E-09 |
| GO:2001141 | regulation of RNA biosynthetic process | Biological Process | 76 | 20 | 2.59E-09 |
| GO:0000978 | RNA polymerase II proximal promoter sequence-specific DNA binding | Molecular Function | 36 | 10 | 2.68E-09 |
| GO:0006366 | transcription by RNA polymerase II | Biological Process | 65 | 16 | 3.03E-09 |
| GO:0016310 | phosphorylation | Biological Process | 55 | 19 | 3.43E-09 |
| GO:0010942 | positive regulation of cell death | Biological Process | 31 | 3 | 3.51E-09 |
| GO:0051716 | cellular response to stimulus | Biological Process | 123 | 41 | 3.80E-09 |
| GO:0042221 | response to chemical | Biological Process | 91 | 26 | 4.07E-09 |
| GO:0009892 | negative regulation of metabolic process | Biological Process | 71 | 16 | 4.37E-09 |
| GO:0010605 | negative regulation of macromolecule metabolic process | Biological Process | 66 | 16 | 4.51E-09 |
| GO:0051252 | regulation of RNA metabolic process | Biological Process | 80 | 20 | 5.47E-09 |
| GO:0006351 | transcription, DNA-templated | Biological Process | 78 | 20 | 6.15E-09 |
| GO:0097659 | nucleic acid-templated transcription | Biological Process | 78 | 20 | 6.33E-09 |
| GO:0031667 | response to nutrient levels | Biological Process | 25 | 4 | 6.74E-09 |
| GO:0072359 | circulatory system development | Biological Process | 31 | 12 | 7.50E-09 |
| GO:0048731 | system development | Biological Process | 82 | 37 | 8.20E-09 |
| GO:0042127 | regulation of cell proliferation | Biological Process | 44 | 12 | 8.21E-09 |
| GO:0051172 | negative regulation of nitrogen compound metabolic process | Biological Process | 59 | 15 | 8.40E-09 |
| GO:0032774 | RNA biosynthetic process | Biological Process | 78 | 20 | 8.54E-09 |
| GO:0031324 | negative regulation of cellular metabolic process | Biological Process | 62 | 15 | 1.06E-08 |
| GO:0048646 | anatomical structure formation involved in morphogenesis | Biological Process | 30 | 13 | 1.12E-08 |
| GO:0044093 | positive regulation of molecular function | Biological Process | 46 | 12 | 1.13E-08 |
| GO:0003700 | DNA-binding transcription factor activity | Molecular Function | 39 | 11 | 1.25E-08 |
| GO:0010648 | negative regulation of cell communication | Biological Process | 39 | 11 | 1.26E-08 |
| GO:0023057 | negative regulation of signaling | Biological Process | 39 | 11 | 1.35E-08 |
| GO:0050789 | regulation of biological process | Biological Process | 157 | 64 | 1.38E-08 |
| GO:0045859 | regulation of protein kinase activity | Biological Process | 29 | 6 | 1.42E-08 |
| GO:0009653 | anatomical structure morphogenesis | Biological Process | 52 | 25 | 1.50E-08 |
| GO:0048513 | animal organ development | Biological Process | 67 | 28 | 1.60E-08 |
| GO:0070887 | cellular response to chemical stimulus | Biological Process | 69 | 19 | 1.89E-08 |
| GO:0140110 | transcription regulator activity | Molecular Function | 48 | 13 | 2.06E-08 |
| GO:0009968 | negative regulation of signal transduction | Biological Process | 37 | 10 | 2.27E-08 |
| GO:0000977 | RNA polymerase II regulatory region sequence-specific DNA binding | Molecular Function | 38 | 11 | 2.27E-08 |
| GO:0001012 | RNA polymerase II regulatory region DNA binding | Molecular Function | 38 | 11 | 2.27E-08 |
| GO:0051239 | regulation of multicellular organismal process | Biological Process | 62 | 25 | 2.37E-08 |
| GO:0051247 | positive regulation of protein metabolic process | Biological Process | 45 | 11 | 2.38E-08 |
| GO:0000981 | DNA-binding transcription factor activity, RNA polymerase II-specific | Molecular Function | 39 | 9 | 2.51E-08 |
| GO:0019219 | regulation of nucleobase-containing compound metabolic process | Biological Process | 84 | 20 | 2.53E-08 |
| GO:0000976 | transcription regulatory region sequence-specific DNA binding | Molecular Function | 40 | 11 | 2.55E-08 |
| GO:0044212 | transcription regulatory region DNA binding | Molecular Function | 40 | 11 | 2.73E-08 |
| GO:0001067 | regulatory region nucleic acid binding | Molecular Function | 40 | 11 | 2.85E-08 |
| GO:0048534 | hematopoietic or lymphoid organ development | Biological Process | 31 | 8 | 3.06E-08 |
| GO:0032270 | positive regulation of cellular protein metabolic process | Biological Process | 43 | 11 | 3.11E-08 |
| GO:0009605 | response to external stimulus | Biological Process | 57 | 19 | 3.47E-08 |
| GO:0048872 | homeostasis of number of cells | Biological Process | 14 | 5 | 3.65E-08 |
| GO:0050896 | response to stimulus | Biological Process | 139 | 46 | 4.03E-08 |
| GO:0007154 | cell communication | Biological Process | 106 | 40 | 4.06E-08 |
| GO:0042326 | negative regulation of phosphorylation | Biological Process | 21 | 4 | 4.09E-08 |
| GO:0014070 | response to organic cyclic compound | Biological Process | 31 | 7 | 4.46E-08 |
| GO:0033993 | response to lipid | Biological Process | 29 | 9 | 4.58E-08 |
| GO:0043565 | sequence-specific DNA binding | Molecular Function | 42 | 12 | 4.90E-08 |
| GO:0005488 | binding | Molecular Function | 189 | 85 | 5.20E-08 |
| GO:0034645 | cellular macromolecule biosynthetic process | Biological Process | 88 | 29 | 6.25E-08 |
| GO:0009059 | macromolecule biosynthetic process | Biological Process | 89 | 29 | 6.81E-08 |
| GO:0043408 | regulation of MAPK cascade | Biological Process | 25 | 7 | 7.07E-08 |
| GO:1901700 | response to oxygen-containing compound | Biological Process | 46 | 8 | 7.88E-08 |
| GO:0043068 | positive regulation of programmed cell death | Biological Process | 27 | 3 | 8.20E-08 |
| GO:0030097 | hemopoiesis | Biological Process | 29 | 8 | 1.00E-07 |
| GO:1990837 | sequence-specific double-stranded DNA binding | Molecular Function | 40 | 11 | 1.06E-07 |
| GO:0030154 | cell differentiation | Biological Process | 72 | 32 | 1.22E-07 |
| GO:0002520 | immune system development | Biological Process | 31 | 8 | 1.24E-07 |
| GO:0000165 | MAPK cascade | Biological Process | 29 | 8 | 1.31E-07 |
| GO:0023014 | signal transduction by protein phosphorylation | Biological Process | 29 | 8 | 1.31E-07 |
| GO:0048869 | cellular developmental process | Biological Process | 74 | 33 | 1.36E-07 |
| GO:0007050 | cell cycle arrest | Biological Process | 14 | 3 | 1.40E-07 |
| GO:0009636 | response to toxic substance | Biological Process | 24 | 2 | 1.77E-07 |
| GO:0032501 | multicellular organismal process | Biological Process | 113 | 47 | 1.86E-07 |
| GO:0042327 | positive regulation of phosphorylation | Biological Process | 31 | 9 | 2.01E-07 |
| GO:0023052 | signaling | Biological Process | 103 | 40 | 2.25E-07 |
| GO:0043065 | positive regulation of apoptotic process | Biological Process | 26 | 3 | 2.30E-07 |
| GO:0031668 | cellular response to extracellular stimulus | Biological Process | 15 | 4 | 2.34E-07 |
| GO:0007165 | signal transduction | Biological Process | 96 | 39 | 2.61E-07 |
| GO:0071900 | regulation of protein serine/threonine kinase activity | Biological Process | 20 | 5 | 2.93E-07 |
| GO:0005515 | protein binding | Molecular Function | 180 | 73 | 3.16E-07 |
| GO:0031401 | positive regulation of protein modification process | Biological Process | 33 | 10 | 3.51E-07 |
| GO:0005634 | nucleus | Cellular Component | 117 | 35 | 3.65E-07 |
| GO:0010562 | positive regulation of phosphorus metabolic process | Biological Process | 32 | 9 | 3.84E-07 |
| GO:0045937 | positive regulation of phosphate metabolic process | Biological Process | 32 | 9 | 3.84E-07 |
| GO:0065007 | biological regulation | Biological Process | 160 | 66 | 4.04E-07 |
| GO:0050790 | regulation of catalytic activity | Biological Process | 50 | 18 | 4.17E-07 |
| GO:0033002 | muscle cell proliferation | Biological Process | 13 | 2 | 4.37E-07 |
| GO:0001933 | negative regulation of protein phosphorylation | Biological Process | 18 | 4 | 4.57E-07 |
| GO:0002521 | leukocyte differentiation | Biological Process | 20 | 6 | 4.60E-07 |
| GO:0050793 | regulation of developmental process | Biological Process | 49 | 23 | 4.62E-07 |
| GO:0048583 | regulation of response to stimulus | Biological Process | 79 | 25 | 4.92E-07 |
| GO:2000026 | regulation of multicellular organismal development | Biological Process | 41 | 20 | 5.20E-07 |
| GO:0035295 | tube development | Biological Process | 27 | 11 | 5.54E-07 |
| GO:0033674 | positive regulation of kinase activity | Biological Process | 22 | 5 | 5.93E-07 |
| GO:1902531 | regulation of intracellular signal transduction | Biological Process | 46 | 11 | 6.16E-07 |
| GO:0003690 | double-stranded DNA binding | Molecular Function | 40 | 11 | 6.23E-07 |
| GO:0051347 | positive regulation of transferase activity | Biological Process | 24 | 5 | 6.57E-07 |
| GO:0046685 | response to arsenic-containing substance | Biological Process | 7 | 0 | 6.62E-07 |
| GO:0071310 | cellular response to organic substance | Biological Process | 54 | 18 | 7.20E-07 |
| GO:0032269 | negative regulation of cellular protein metabolic process | Biological Process | 33 | 5 | 7.34E-07 |
| GO:0030099 | myeloid cell differentiation | Biological Process | 18 | 4 | 7.66E-07 |
| GO:0043170 | macromolecule metabolic process | Biological Process | 140 | 46 | 8.30E-07 |
| GO:0044092 | negative regulation of molecular function | Biological Process | 34 | 6 | 8.91E-07 |
| GO:0045936 | negative regulation of phosphate metabolic process | Biological Process | 22 | 4 | 9.21E-07 |
| GO:0050673 | epithelial cell proliferation | Biological Process | 13 | 8 | 9.48E-07 |
| GO:0010563 | negative regulation of phosphorus metabolic process | Biological Process | 22 | 4 | 9.52E-07 |
| GO:0048661 | positive regulation of smooth muscle cell proliferation | Biological Process | 8 | 2 | 9.96E-07 |
| GO:0007166 | cell surface receptor signaling pathway | Biological Process | 55 | 24 | 1.05E-06 |
| GO:0000122 | negative regulation of transcription by RNA polymerase II | Biological Process | 28 | 6 | 1.08E-06 |
| GO:0051248 | negative regulation of protein metabolic process | Biological Process | 34 | 5 | 1.16E-06 |
| GO:2000113 | negative regulation of cellular macromolecule biosynthetic process | Biological Process | 39 | 9 | 1.18E-06 |
| GO:0034654 | nucleobase-containing compound biosynthetic process | Biological Process | 80 | 21 | 1.20E-06 |
| GO:0051094 | positive regulation of developmental process | Biological Process | 31 | 14 | 1.39E-06 |
| GO:0010558 | negative regulation of macromolecule biosynthetic process | Biological Process | 39 | 9 | 1.44E-06 |
| GO:0042542 | response to hydrogen peroxide | Biological Process | 11 | 1 | 1.45E-06 |
| GO:0061061 | muscle structure development | Biological Process | 22 | 5 | 1.46E-06 |
| GO:0010629 | negative regulation of gene expression | Biological Process | 47 | 11 | 1.53E-06 |
| GO:0009890 | negative regulation of biosynthetic process | Biological Process | 41 | 9 | 1.58E-06 |
| GO:0019438 | aromatic compound biosynthetic process | Biological Process | 81 | 21 | 1.59E-06 |
| GO:0044271 | cellular nitrogen compound biosynthetic process | Biological Process | 90 | 23 | 1.64E-06 |
| GO:0000785 | chromatin | Cellular Component | 34 | 6 | 1.66E-06 |
| GO:0001944 | vasculature development | Biological Process | 24 | 5 | 1.70E-06 |
| GO:0072358 | cardiovascular system development | Biological Process | 24 | 5 | 1.70E-06 |
| GO:0048660 | regulation of smooth muscle cell proliferation | Biological Process | 10 | 2 | 1.82E-06 |
| GO:0001934 | positive regulation of protein phosphorylation | Biological Process | 28 | 8 | 1.94E-06 |
| GO:0008284 | positive regulation of cell proliferation | Biological Process | 27 | 7 | 2.19E-06 |
| GO:0048659 | smooth muscle cell proliferation | Biological Process | 10 | 2 | 2.27E-06 |
| GO:0051240 | positive regulation of multicellular organismal process | Biological Process | 40 | 13 | 2.28E-06 |
| GO:0033673 | negative regulation of kinase activity | Biological Process | 13 | 3 | 2.51E-06 |
| GO:0009628 | response to abiotic stimulus | Biological Process | 39 | 2 | 2.59E-06 |
| GO:0018130 | heterocycle biosynthetic process | Biological Process | 80 | 21 | 2.68E-06 |
| GO:0009058 | biosynthetic process | Biological Process | 101 | 32 | 2.70E-06 |
| GO:0001822 | kidney development | Biological Process | 11 | 6 | 2.70E-06 |
| GO:0031400 | negative regulation of protein modification process | Biological Process | 22 | 4 | 3.15E-06 |
| GO:0060548 | negative regulation of cell death | Biological Process | 30 | 5 | 3.37E-06 |
| GO:0051348 | negative regulation of transferase activity | Biological Process | 14 | 3 | 3.40E-06 |
| GO:0008134 | transcription factor binding | Molecular Function | 24 | 3 | 3.40E-06 |
| GO:0006464 | cellular protein modification process | Biological Process | 70 | 29 | 3.59E-06 |
| GO:0036211 | protein modification process | Biological Process | 70 | 29 | 3.59E-06 |
| GO:0006469 | negative regulation of protein kinase activity | Biological Process | 12 | 3 | 3.88E-06 |
| GO:0043085 | positive regulation of catalytic activity | Biological Process | 35 | 9 | 3.95E-06 |
| GO:0072001 | renal system development | Biological Process | 11 | 6 | 4.06E-06 |
| GO:1901362 | organic cyclic compound biosynthetic process | Biological Process | 82 | 21 | 4.17E-06 |
| GO:0080135 | regulation of cellular response to stress | Biological Process | 23 | 6 | 4.21E-06 |
| GO:0045597 | positive regulation of cell differentiation | Biological Process | 25 | 10 | 4.39E-06 |
| GO:0040011 | locomotion | Biological Process | 36 | 19 | 4.52E-06 |
| GO:0001655 | urogenital system development | Biological Process | 11 | 7 | 4.58E-06 |
| GO:0031327 | negative regulation of cellular biosynthetic process | Biological Process | 39 | 9 | 5.00E-06 |
| GO:0002262 | myeloid cell homeostasis | Biological Process | 10 | 2 | 5.20E-06 |
| GO:2001236 | regulation of extrinsic apoptotic signaling pathway | Biological Process | 11 | 1 | 5.20E-06 |
| GO:0050678 | regulation of epithelial cell proliferation | Biological Process | 10 | 8 | 5.39E-06 |
| GO:0031669 | cellular response to nutrient levels | Biological Process | 13 | 3 | 5.40E-06 |
| GO:0035239 | tube morphogenesis | Biological Process | 22 | 9 | 5.46E-06 |
| GO:0080134 | regulation of response to stress | Biological Process | 42 | 9 | 5.49E-06 |
| GO:1901701 | cellular response to oxygen-containing compound | Biological Process | 31 | 7 | 5.77E-06 |
| GO:0097237 | cellular response to toxic substance | Biological Process | 15 | 0 | 5.80E-06 |
| GO:0097190 | apoptotic signaling pathway | Biological Process | 24 | 1 | 5.82E-06 |
| GO:0044249 | cellular biosynthetic process | Biological Process | 97 | 32 | 5.85E-06 |
| GO:0002573 | myeloid leukocyte differentiation | Biological Process | 11 | 3 | 6.25E-06 |
| GO:0071901 | negative regulation of protein serine/threonine kinase activity | Biological Process | 8 | 3 | 6.51E-06 |
| GO:0001568 | blood vessel development | Biological Process | 22 | 5 | 6.52E-06 |
| GO:0009725 | response to hormone | Biological Process | 31 | 4 | 6.74E-06 |
| GO:0009966 | regulation of signal transduction | Biological Process | 58 | 20 | 6.89E-06 |
| GO:1901698 | response to nitrogen compound | Biological Process | 32 | 5 | 6.95E-06 |
| GO:0005654 | nucleoplasm | Cellular Component | 73 | 19 | 7.03E-06 |
| GO:0016477 | cell migration | Biological Process | 32 | 14 | 7.06E-06 |
| GO:0006979 | response to oxidative stress | Biological Process | 20 | 1 | 7.12E-06 |
| GO:1902532 | negative regulation of intracellular signal transduction | Biological Process | 20 | 3 | 7.30E-06 |
| GO:0010467 | gene expression | Biological Process | 95 | 26 | 7.72E-06 |
| GO:0060429 | epithelium development | Biological Process | 28 | 13 | 8.59E-06 |
| GO:0097191 | extrinsic apoptotic signaling pathway | Biological Process | 13 | 1 | 8.99E-06 |
| GO:0045595 | regulation of cell differentiation | Biological Process | 35 | 18 | 9.10E-06 |
| GO:0097193 | intrinsic apoptotic signaling pathway | Biological Process | 15 | 1 | 1.01E-05 |
| GO:0051409 | response to nitrosative stress | Biological Process | 4 | 0 | 1.02E-05 |
| GO:0042493 | response to drug | Biological Process | 30 | 5 | 1.04E-05 |
| GO:0048608 | reproductive structure development | Biological Process | 16 | 4 | 1.06E-05 |
| GO:0051090 | regulation of DNA-binding transcription factor activity | Biological Process | 17 | 3 | 1.13E-05 |
| GO:0000302 | response to reactive oxygen species | Biological Process | 13 | 1 | 1.16E-05 |
| GO:0061458 | reproductive system development | Biological Process | 16 | 4 | 1.17E-05 |
| GO:1901576 | organic substance biosynthetic process | Biological Process | 97 | 32 | 1.20E-05 |
| GO:0006796 | phosphate-containing compound metabolic process | Biological Process | 59 | 22 | 1.24E-05 |
| GO:0043409 | negative regulation of MAPK cascade | Biological Process | 10 | 2 | 1.24E-05 |
| GO:0043066 | negative regulation of apoptotic process | Biological Process | 26 | 5 | 1.28E-05 |
| GO:0071243 | cellular response to arsenic-containing substance | Biological Process | 5 | 0 | 1.31E-05 |
| GO:0044752 | response to human chorionic gonadotropin | Biological Process | 3 | 0 | 1.38E-05 |
| GO:0001890 | placenta development | Biological Process | 8 | 3 | 1.38E-05 |
| GO:0042594 | response to starvation | Biological Process | 13 | 1 | 1.40E-05 |
| GO:0032147 | activation of protein kinase activity | Biological Process | 16 | 1 | 1.41E-05 |
| GO:0006928 | movement of cell or subcellular component | Biological Process | 36 | 22 | 1.63E-05 |
| GO:0006793 | phosphorus metabolic process | Biological Process | 59 | 22 | 1.67E-05 |
| GO:0009719 | response to endogenous stimulus | Biological Process | 37 | 11 | 1.67E-05 |
| GO:0051384 | response to glucocorticoid | Biological Process | 11 | 0 | 1.79E-05 |
| GO:0002040 | sprouting angiogenesis | Biological Process | 8 | 2 | 2.01E-05 |
| GO:0002376 | immune system process | Biological Process | 62 | 16 | 2.09E-05 |
| GO:0043412 | macromolecule modification | Biological Process | 71 | 29 | 2.10E-05 |
| GO:0010646 | regulation of cell communication | Biological Process | 62 | 21 | 2.11E-05 |
| GO:0043069 | negative regulation of programmed cell death | Biological Process | 26 | 5 | 2.15E-05 |
| GO:0045860 | positive regulation of protein kinase activity | Biological Process | 20 | 2 | 2.23E-05 |
| GO:2000145 | regulation of cell motility | Biological Process | 21 | 11 | 2.32E-05 |
| GO:0072537 | fibroblast activation | Biological Process | 4 | 0 | 2.36E-05 |
| GO:0009790 | embryo development | Biological Process | 24 | 10 | 2.41E-05 |
| GO:0043281 | regulation of cysteine-type endopeptidase activity involved in apoptotic process | Biological Process | 11 | 2 | 2.42E-05 |
| GO:0071229 | cellular response to acid chemical | Biological Process | 10 | 3 | 2.42E-05 |
| GO:0034101 | erythrocyte homeostasis | Biological Process | 8 | 2 | 2.47E-05 |
| GO:1901652 | response to peptide | Biological Process | 21 | 1 | 2.65E-05 |
| GO:0048870 | cell motility | Biological Process | 32 | 16 | 2.68E-05 |
| GO:0051674 | localization of cell | Biological Process | 32 | 16 | 2.68E-05 |
| GO:0016070 | RNA metabolic process | Biological Process | 83 | 20 | 2.69E-05 |
| GO:0009967 | positive regulation of signal transduction | Biological Process | 37 | 10 | 2.71E-05 |
| GO:0051241 | negative regulation of multicellular organismal process | Biological Process | 28 | 10 | 2.79E-05 |
| GO:0045892 | negative regulation of transcription, DNA-templated | Biological Process | 31 | 9 | 2.80E-05 |
| GO:0048545 | response to steroid hormone | Biological Process | 15 | 3 | 2.86E-05 |
| GO:1903507 | negative regulation of nucleic acid-templated transcription | Biological Process | 31 | 9 | 2.90E-05 |
| GO:0009612 | response to mechanical stimulus | Biological Process | 12 | 1 | 2.94E-05 |
| GO:1902679 | negative regulation of RNA biosynthetic process | Biological Process | 31 | 9 | 3.00E-05 |
| GO:0017017 | MAP kinase tyrosine/serine/threonine phosphatase activity | Molecular Function | 4 | 0 | 3.22E-05 |
| GO:0036003 | positive regulation of transcription from RNA polymerase II promoter in response to stress | Biological Process | 5 | 0 | 3.26E-05 |
| GO:0009408 | response to heat | Biological Process | 11 | 0 | 3.27E-05 |
| GO:0023051 | regulation of signaling | Biological Process | 62 | 21 | 3.38E-05 |
| GO:0044424 | intracellular part | Cellular Component | 172 | 70 | 3.39E-05 |
| GO:0032762 | mast cell cytokine production | Biological Process | 3 | 0 | 3.41E-05 |
| GO:0032763 | regulation of mast cell cytokine production | Biological Process | 3 | 0 | 3.41E-05 |
| GO:0005622 | intracellular | Cellular Component | 172 | 70 | 3.42E-05 |
| GO:0045934 | negative regulation of nucleobase-containing compound metabolic process | Biological Process | 35 | 10 | 3.57E-05 |
| GO:0051253 | negative regulation of RNA metabolic process | Biological Process | 32 | 10 | 3.69E-05 |
| GO:0019221 | cytokine-mediated signaling pathway | Biological Process | 22 | 6 | 3.71E-05 |
| GO:0001817 | regulation of cytokine production | Biological Process | 23 | 4 | 3.73E-05 |
| GO:0010243 | response to organonitrogen compound | Biological Process | 30 | 3 | 3.73E-05 |
| GO:0031981 | nuclear lumen | Cellular Component | 75 | 20 | 3.85E-05 |
| GO:0030334 | regulation of cell migration | Biological Process | 21 | 9 | 3.89E-05 |
| GO:0044706 | multi-multicellular organism process | Biological Process | 12 | 1 | 3.90E-05 |
| GO:0001816 | cytokine production | Biological Process | 23 | 4 | 4.17E-05 |
| GO:0043491 | protein kinase B signaling | Biological Process | 11 | 3 | 4.53E-05 |
| GO:0030330 | DNA damage response, signal transduction by p53 class mediator | Biological Process | 8 | 1 | 4.66E-05 |
| GO:0070059 | intrinsic apoptotic signaling pathway in response to endoplasmic reticulum stress | Biological Process | 7 | 0 | 4.67E-05 |
| GO:0071407 | cellular response to organic cyclic compound | Biological Process | 16 | 6 | 4.71E-05 |
| GO:0040007 | growth | Biological Process | 24 | 7 | 5.07E-05 |
| GO:0009266 | response to temperature stimulus | Biological Process | 13 | 0 | 5.13E-05 |
| GO:0044267 | cellular protein metabolic process | Biological Process | 82 | 30 | 5.21E-05 |
| GO:0048584 | positive regulation of response to stimulus | Biological Process | 50 | 12 | 5.25E-05 |
| GO:0044237 | cellular metabolic process | Biological Process | 149 | 49 | 5.26E-05 |
| GO:0031098 | stress-activated protein kinase signaling cascade | Biological Process | 12 | 3 | 5.30E-05 |
| GO:0031960 | response to corticosteroid | Biological Process | 11 | 0 | 5.42E-05 |
| GO:0009792 | embryo development ending in birth or egg hatching | Biological Process | 16 | 8 | 5.58E-05 |
| GO:0022603 | regulation of anatomical structure morphogenesis | Biological Process | 21 | 13 | 5.62E-05 |
| GO:0030335 | positive regulation of cell migration | Biological Process | 13 | 8 | 5.72E-05 |
| GO:0034698 | response to gonadotropin | Biological Process | 5 | 0 | 5.80E-05 |
| GO:0048468 | cell development | Biological Process | 35 | 21 | 5.97E-05 |
| GO:0033549 | MAP kinase phosphatase activity | Molecular Function | 4 | 0 | 6.01E-05 |
| GO:0043405 | regulation of MAP kinase activity | Biological Process | 14 | 2 | 6.04E-05 |
| GO:0043534 | blood vessel endothelial cell migration | Biological Process | 7 | 2 | 6.17E-05 |
| GO:0046677 | response to antibiotic | Biological Process | 14 | 2 | 6.25E-05 |
| GO:0032354 | response to follicle-stimulating hormone | Biological Process | 4 | 0 | 6.27E-05 |
| GO:0001701 | in utero embryonic development | Biological Process | 13 | 4 | 6.55E-05 |
| GO:0007517 | muscle organ development | Biological Process | 14 | 3 | 6.55E-05 |
| GO:0034599 | cellular response to oxidative stress | Biological Process | 14 | 1 | 6.64E-05 |
| GO:1903708 | positive regulation of hemopoiesis | Biological Process | 11 | 1 | 7.09E-05 |
| GO:0051091 | positive regulation of DNA-binding transcription factor activity | Biological Process | 11 | 3 | 7.09E-05 |
| GO:1901342 | regulation of vasculature development | Biological Process | 14 | 2 | 7.19E-05 |
| GO:0044428 | nuclear part | Cellular Component | 79 | 21 | 7.29E-05 |
| GO:2000116 | regulation of cysteine-type endopeptidase activity | Biological Process | 11 | 2 | 7.29E-05 |
| GO:0034097 | response to cytokine | Biological Process | 30 | 6 | 7.68E-05 |
| GO:0030218 | erythrocyte differentiation | Biological Process | 7 | 2 | 8.07E-05 |
| GO:0008285 | negative regulation of cell proliferation | Biological Process | 16 | 9 | 8.57E-05 |
| GO:0001704 | formation of primary germ layer | Biological Process | 7 | 2 | 8.62E-05 |
| GO:0070371 | ERK1 and ERK2 cascade | Biological Process | 11 | 4 | 9.22E-05 |
| GO:1902533 | positive regulation of intracellular signal transduction | Biological Process | 29 | 5 | 9.22E-05 |
| GO:0043009 | chordate embryonic development | Biological Process | 15 | 8 | 9.45E-05 |
| GO:0040012 | regulation of locomotion | Biological Process | 21 | 11 | 9.48E-05 |
| GO:0071495 | cellular response to endogenous stimulus | Biological Process | 29 | 11 | 9.52E-05 |
| GO:0043232 | intracellular non-membrane-bounded organelle | Cellular Component | 79 | 24 | 9.87E-05 |
| GO:0019899 | enzyme binding | Molecular Function | 41 | 14 | 0.000103 |
| GO:0043228 | non-membrane-bounded organelle | Cellular Component | 79 | 24 | 0.000105 |
| GO:0007369 | gastrulation | Biological Process | 9 | 2 | 0.000106 |
| GO:0071850 | mitotic cell cycle arrest | Biological Process | 4 | 0 | 0.000107 |
| GO:2000147 | positive regulation of cell motility | Biological Process | 13 | 8 | 0.000107 |
| GO:0098739 | import across plasma membrane | Biological Process | 7 | 3 | 0.000108 |
| GO:0043231 | intracellular membrane-bounded organelle | Cellular Component | 145 | 50 | 0.00011 |
| GO:0044427 | chromosomal part | Cellular Component | 36 | 7 | 0.000112 |
| GO:0051403 | stress-activated MAPK cascade | Biological Process | 12 | 2 | 0.000113 |
| GO:0043086 | negative regulation of catalytic activity | Biological Process | 21 | 6 | 0.000114 |
| GO:0001101 | response to acid chemical | Biological Process | 13 | 3 | 0.000115 |
| GO:0032870 | cellular response to hormone stimulus | Biological Process | 21 | 4 | 0.000117 |
| GO:0097647 | amylin receptor signaling pathway | Biological Process | 3 | 0 | 0.000117 |
| GO:0006986 | response to unfolded protein | Biological Process | 11 | 0 | 0.000117 |
| GO:0070373 | negative regulation of ERK1 and ERK2 cascade | Biological Process | 5 | 2 | 0.000121 |
| GO:0051270 | regulation of cellular component movement | Biological Process | 21 | 11 | 0.000122 |
| GO:0070848 | response to growth factor | Biological Process | 17 | 8 | 0.000124 |
| GO:0030155 | regulation of cell adhesion | Biological Process | 18 | 7 | 0.000127 |
| GO:0006750 | glutathione biosynthetic process | Biological Process | 4 | 0 | 0.000136 |
| GO:2001233 | regulation of apoptotic signaling pathway | Biological Process | 16 | 1 | 0.000139 |
| GO:0048514 | blood vessel morphogenesis | Biological Process | 17 | 5 | 0.000149 |
| GO:0006807 | nitrogen compound metabolic process | Biological Process | 142 | 46 | 0.000149 |
| GO:0010837 | regulation of keratinocyte proliferation | Biological Process | 2 | 3 | 0.000152 |
| GO:0010647 | positive regulation of cell communication | Biological Process | 38 | 10 | 0.000155 |
| GO:0070372 | regulation of ERK1 and ERK2 cascade | Biological Process | 10 | 4 | 0.000156 |
| GO:0048732 | gland development | Biological Process | 12 | 6 | 0.00016 |
| GO:0002042 | cell migration involved in sprouting angiogenesis | Biological Process | 4 | 2 | 0.000164 |
| GO:0071704 | organic substance metabolic process | Biological Process | 151 | 49 | 0.000165 |
| GO:0023056 | positive regulation of signaling | Biological Process | 38 | 10 | 0.000167 |
| GO:0051272 | positive regulation of cellular component movement | Biological Process | 13 | 8 | 0.000168 |
| GO:0030728 | ovulation | Biological Process | 4 | 0 | 0.00017 |
| GO:0003006 | developmental process involved in reproduction | Biological Process | 20 | 4 | 0.00017 |
| GO:0009267 | cellular response to starvation | Biological Process | 10 | 1 | 0.000171 |
| GO:0035296 | regulation of tube diameter | Biological Process | 7 | 2 | 0.00018 |
| GO:0050880 | regulation of blood vessel size | Biological Process | 7 | 2 | 0.00018 |
| GO:0097746 | regulation of blood vessel diameter | Biological Process | 7 | 2 | 0.00018 |
| GO:0007507 | heart development | Biological Process | 13 | 8 | 0.00018 |
| GO:0071396 | cellular response to lipid | Biological Process | 15 | 7 | 0.000183 |
| GO:0035733 | hepatic stellate cell activation | Biological Process | 3 | 0 | 0.000184 |
| GO:0097646 | calcitonin family receptor signaling pathway | Biological Process | 3 | 0 | 0.000184 |
| GO:1901724 | positive regulation of cell proliferation involved in kidney development | Biological Process | 3 | 0 | 0.000184 |
| GO:0035150 | regulation of tube size | Biological Process | 7 | 2 | 0.00019 |
| GO:0071345 | cellular response to cytokine stimulus | Biological Process | 27 | 6 | 0.000192 |
| GO:0003018 | vascular process in circulatory system | Biological Process | 8 | 2 | 0.000202 |
| GO:0019184 | nonribosomal peptide biosynthetic process | Biological Process | 4 | 0 | 0.00021 |
| GO:0017109 | glutamate-cysteine ligase complex | Cellular Component | 2 | 0 | 0.000214 |
| GO:0150058 | amylin receptor complex 3 | Cellular Component | 2 | 0 | 0.000214 |
| GO:1990184 | amino acid transport complex | Cellular Component | 2 | 0 | 0.000214 |
| GO:1990622 | CHOP-ATF3 complex | Cellular Component | 2 | 0 | 0.000214 |
| GO:0048729 | tissue morphogenesis | Biological Process | 16 | 7 | 0.000214 |
| GO:0071260 | cellular response to mechanical stimulus | Biological Process | 7 | 0 | 0.000215 |
| GO:0003677 | DNA binding | Molecular Function | 46 | 13 | 0.000219 |
| GO:0071453 | cellular response to oxygen levels | Biological Process | 11 | 1 | 0.000219 |
| GO:1902041 | regulation of extrinsic apoptotic signaling pathway via death domain receptors | Biological Process | 6 | 0 | 0.000222 |
| GO:0042770 | signal transduction in response to DNA damage | Biological Process | 8 | 1 | 0.000225 |
| GO:0004357 | glutamate-cysteine ligase activity | Molecular Function | 2 | 0 | 0.000226 |
| GO:0032542 | sulfiredoxin activity | Molecular Function | 2 | 0 | 0.000226 |
| GO:0040017 | positive regulation of locomotion | Biological Process | 13 | 8 | 0.000228 |
| GO:0015823 | phenylalanine transport | Biological Process | 2 | 0 | 0.000231 |
| GO:0038041 | cross-receptor inhibition within G protein-coupled receptor heterodimer | Biological Process | 2 | 0 | 0.000231 |
| GO:0097069 | cellular response to thyroxine stimulus | Biological Process | 2 | 0 | 0.000231 |
| GO:0097532 | stress response to acid chemical | Biological Process | 2 | 0 | 0.000231 |
| GO:0097533 | cellular stress response to acid chemical | Biological Process | 2 | 0 | 0.000231 |
| GO:1904387 | cellular response to L-phenylalanine derivative | Biological Process | 2 | 0 | 0.000231 |
| GO:0008152 | metabolic process | Biological Process | 155 | 51 | 0.000235 |
| GO:0097648 | G protein-coupled receptor complex | Cellular Component | 2 | 1 | 0.000244 |
| GO:0043410 | positive regulation of MAPK cascade | Biological Process | 16 | 4 | 0.000245 |
| GO:0001570 | vasculogenesis | Biological Process | 5 | 2 | 0.000251 |
| GO:0005623 | cell | Cellular Component | 183 | 81 | 0.000253 |
| GO:0044464 | cell part | Cellular Component | 183 | 81 | 0.000253 |
| GO:0033613 | activating transcription factor binding | Molecular Function | 7 | 0 | 0.000254 |
| GO:0048589 | developmental growth | Biological Process | 15 | 7 | 0.000262 |
| GO:0008625 | extrinsic apoptotic signaling pathway via death domain receptors | Biological Process | 7 | 0 | 0.000271 |
| GO:1903589 | positive regulation of blood vessel endothelial cell proliferation involved in sprouting angiogenesis | Biological Process | 2 | 1 | 0.000274 |
| GO:0072073 | kidney epithelium development | Biological Process | 5 | 4 | 0.00028 |
| GO:0009791 | post-embryonic development | Biological Process | 5 | 2 | 0.000292 |
| GO:0043618 | regulation of transcription from RNA polymerase II promoter in response to stress | Biological Process | 8 | 0 | 0.000296 |
| GO:1905898 | positive regulation of response to endoplasmic reticulum stress | Biological Process | 5 | 0 | 0.000296 |
| GO:0060537 | muscle tissue development | Biological Process | 14 | 2 | 0.000305 |
| GO:0051412 | response to corticosterone | Biological Process | 4 | 0 | 0.000309 |
| GO:2001235 | positive regulation of apoptotic signaling pathway | Biological Process | 10 | 0 | 0.000311 |
| GO:0002682 | regulation of immune system process | Biological Process | 40 | 6 | 0.000318 |
| GO:0044238 | primary metabolic process | Biological Process | 145 | 48 | 0.000323 |
| GO:0035966 | response to topologically incorrect protein | Biological Process | 11 | 0 | 0.000328 |
| GO:0071236 | cellular response to antibiotic | Biological Process | 9 | 0 | 0.000346 |
| GO:0065008 | regulation of biological quality | Biological Process | 65 | 23 | 0.000355 |
| GO:0001525 | angiogenesis | Biological Process | 15 | 4 | 0.000359 |
| GO:1901214 | regulation of neuron death | Biological Process | 13 | 1 | 0.000363 |
| GO:0002043 | blood vessel endothelial cell proliferation involved in sprouting angiogenesis | Biological Process | 3 | 1 | 0.00037 |
| GO:0045649 | regulation of macrophage differentiation | Biological Process | 3 | 1 | 0.00037 |
| GO:0090304 | nucleic acid metabolic process | Biological Process | 87 | 20 | 0.000371 |
| GO:0008330 | protein tyrosine/threonine phosphatase activity | Molecular Function | 3 | 0 | 0.000374 |
| GO:0090184 | positive regulation of kidney development | Biological Process | 5 | 0 | 0.000376 |
| GO:0070997 | neuron death | Biological Process | 14 | 1 | 0.000384 |
| GO:0045604 | regulation of epidermal cell differentiation | Biological Process | 6 | 0 | 0.000386 |
| GO:0071372 | cellular response to follicle-stimulating hormone stimulus | Biological Process | 3 | 0 | 0.000386 |
| GO:0097084 | vascular smooth muscle cell development | Biological Process | 3 | 0 | 0.000386 |
| GO:0042592 | homeostatic process | Biological Process | 37 | 11 | 0.000399 |
| GO:0036294 | cellular response to decreased oxygen levels | Biological Process | 10 | 1 | 0.000402 |
| GO:0045682 | regulation of epidermis development | Biological Process | 7 | 0 | 0.000416 |
| GO:0043620 | regulation of DNA-templated transcription in response to stress | Biological Process | 8 | 0 | 0.000423 |
| GO:0002053 | positive regulation of mesenchymal cell proliferation | Biological Process | 3 | 1 | 0.000438 |
| GO:0045786 | negative regulation of cell cycle | Biological Process | 18 | 3 | 0.000445 |
| GO:0043535 | regulation of blood vessel endothelial cell migration | Biological Process | 6 | 1 | 0.000445 |
| GO:0070301 | cellular response to hydrogen peroxide | Biological Process | 7 | 0 | 0.000476 |
| GO:0005694 | chromosome | Cellular Component | 37 | 7 | 0.000498 |
| GO:0002761 | regulation of myeloid leukocyte differentiation | Biological Process | 6 | 2 | 0.000501 |
| GO:0051093 | negative regulation of developmental process | Biological Process | 20 | 9 | 0.00052 |
| GO:0036462 | TRAIL-activated apoptotic signaling pathway | Biological Process | 3 | 0 | 0.000525 |
| GO:1902237 | positive regulation of endoplasmic reticulum stress-induced intrinsic apoptotic signaling pathway | Biological Process | 3 | 0 | 0.000525 |
| GO:0040008 | regulation of growth | Biological Process | 19 | 3 | 0.000525 |
| GO:0048286 | lung alveolus development | Biological Process | 4 | 1 | 0.000526 |
| GO:0089718 | amino acid import across plasma membrane | Biological Process | 5 | 0 | 0.000526 |
| GO:0032642 | regulation of chemokine production | Biological Process | 6 | 1 | 0.000543 |
| GO:0046620 | regulation of organ growth | Biological Process | 6 | 1 | 0.000543 |
| GO:0032879 | regulation of localization | Biological Process | 42 | 21 | 0.000545 |
| GO:0045444 | fat cell differentiation | Biological Process | 7 | 4 | 0.000549 |
| GO:0032602 | chemokine production | Biological Process | 6 | 1 | 0.00058 |
| GO:0035914 | skeletal muscle cell differentiation | Biological Process | 6 | 0 | 0.000584 |
| GO:0038066 | p38MAPK cascade | Biological Process | 5 | 0 | 0.000584 |
| GO:0043616 | keratinocyte proliferation | Biological Process | 2 | 3 | 0.000584 |
| GO:0097755 | positive regulation of blood vessel diameter | Biological Process | 4 | 1 | 0.000584 |
| GO:0030856 | regulation of epithelial cell differentiation | Biological Process | 8 | 1 | 0.000595 |
| GO:0000188 | inactivation of MAPK activity | Biological Process | 4 | 0 | 0.000602 |
| GO:0019538 | protein metabolic process | Biological Process | 86 | 32 | 0.000603 |
| GO:0035690 | cellular response to drug | Biological Process | 13 | 2 | 0.000607 |
| GO:0043434 | response to peptide hormone | Biological Process | 16 | 1 | 0.000634 |
| GO:0071364 | cellular response to epidermal growth factor stimulus | Biological Process | 4 | 1 | 0.000647 |
| GO:0051101 | regulation of DNA binding | Biological Process | 8 | 0 | 0.000657 |
| GO:1902282 | voltage-gated potassium channel activity involved in ventricular cardiac muscle cell action potential repolarization | Molecular Function | 1 | 2 | 0.000671 |
| GO:0003150 | muscular septum morphogenesis | Biological Process | 1 | 1 | 0.000685 |
| GO:0032765 | positive regulation of mast cell cytokine production | Biological Process | 2 | 0 | 0.000685 |
| GO:0060948 | cardiac vascular smooth muscle cell development | Biological Process | 2 | 0 | 0.000685 |
| GO:1901491 | negative regulation of lymphangiogenesis | Biological Process | 2 | 0 | 0.000685 |
| GO:1905665 | positive regulation of calcium ion import across plasma membrane | Biological Process | 2 | 0 | 0.000685 |
| GO:0007584 | response to nutrient | Biological Process | 8 | 3 | 0.000688 |
| GO:0008585 | female gonad development | Biological Process | 6 | 1 | 0.0007 |
| GO:0007498 | mesoderm development | Biological Process | 7 | 1 | 0.000769 |
| GO:0002762 | negative regulation of myeloid leukocyte differentiation | Biological Process | 3 | 2 | 0.000789 |
| GO:0030225 | macrophage differentiation | Biological Process | 4 | 1 | 0.000789 |
| GO:2001238 | positive regulation of extrinsic apoptotic signaling pathway | Biological Process | 5 | 0 | 0.000789 |
| GO:0007565 | female pregnancy | Biological Process | 10 | 0 | 0.000799 |
| GO:0090200 | positive regulation of release of cytochrome c from mitochondria | Biological Process | 4 | 0 | 0.000805 |
| GO:0048598 | embryonic morphogenesis | Biological Process | 12 | 8 | 0.000817 |
| GO:0001102 | RNA polymerase II activating transcription factor binding | Molecular Function | 5 | 0 | 0.000826 |
| GO:0003151 | outflow tract morphogenesis | Biological Process | 4 | 2 | 0.000852 |
| GO:0034614 | cellular response to reactive oxygen species | Biological Process | 8 | 1 | 0.000855 |
| GO:0005035 | death receptor activity | Molecular Function | 3 | 0 | 0.000862 |
| GO:0043090 | amino acid import | Biological Process | 5 | 0 | 0.000868 |
| GO:0048146 | positive regulation of fibroblast proliferation | Biological Process | 4 | 1 | 0.000868 |
| GO:0001227 | DNA-binding transcription repressor activity, RNA polymerase II-specific | Molecular Function | 12 | 1 | 0.000882 |
| GO:0000082 | G1/S transition of mitotic cell cycle | Biological Process | 8 | 3 | 0.000885 |
| GO:0045765 | regulation of angiogenesis | Biological Process | 11 | 2 | 0.00089 |
| GO:0072216 | positive regulation of metanephros development | Biological Process | 3 | 0 | 0.00089 |
| GO:1901722 | regulation of cell proliferation involved in kidney development | Biological Process | 3 | 0 | 0.00089 |
| GO:0045639 | positive regulation of myeloid cell differentiation | Biological Process | 6 | 1 | 0.00089 |
| GO:0090066 | regulation of anatomical structure size | Biological Process | 11 | 7 | 0.000913 |
| GO:0009887 | animal organ morphogenesis | Biological Process | 20 | 10 | 0.000931 |
| GO:0046545 | development of primary female sexual characteristics | Biological Process | 6 | 1 | 0.000944 |
| GO:0051896 | regulation of protein kinase B signaling | Biological Process | 10 | 1 | 0.000949 |
| GO:0070849 | response to epidermal growth factor | Biological Process | 4 | 1 | 0.000952 |
| GO:0030855 | epithelial cell differentiation | Biological Process | 19 | 5 | 0.000988 |
| GO:0071363 | cellular response to growth factor stimulus | Biological Process | 15 | 7 | 0.001033 |
| GO:0071456 | cellular response to hypoxia | Biological Process | 9 | 1 | 0.001047 |
| GO:0044389 | ubiquitin-like protein ligase binding | Molecular Function | 9 | 4 | 0.00105 |
| GO:0043407 | negative regulation of MAP kinase activity | Biological Process | 5 | 1 | 0.001054 |
| GO:0008630 | intrinsic apoptotic signaling pathway in response to DNA damage | Biological Process | 6 | 1 | 0.001058 |
| GO:0022407 | regulation of cell-cell adhesion | Biological Process | 13 | 3 | 0.001073 |
| GO:0007423 | sensory organ development | Biological Process | 9 | 10 | 0.001093 |
| GO:0043227 | membrane-bounded organelle | Cellular Component | 155 | 55 | 0.001118 |
| GO:0070482 | response to oxygen levels | Biological Process | 14 | 1 | 0.001171 |
| GO:1901363 | heterocyclic compound binding | Molecular Function | 87 | 27 | 0.001171 |
| GO:2001237 | negative regulation of extrinsic apoptotic signaling pathway | Biological Process | 6 | 1 | 0.001183 |
| GO:0044344 | cellular response to fibroblast growth factor stimulus | Biological Process | 6 | 2 | 0.001196 |
| GO:0002683 | negative regulation of immune system process | Biological Process | 19 | 2 | 0.001236 |
| GO:0072132 | mesenchyme morphogenesis | Biological Process | 4 | 1 | 0.001243 |
| GO:2000134 | negative regulation of G1/S transition of mitotic cell cycle | Biological Process | 5 | 2 | 0.00125 |
| GO:0032449 | CBM complex | Cellular Component | 2 | 0 | 0.001257 |
| GO:1903440 | amylin receptor complex | Cellular Component | 2 | 0 | 0.001257 |
| GO:0001503 | ossification | Biological Process | 8 | 7 | 0.001293 |
| GO:0097643 | amylin receptor activity | Molecular Function | 2 | 0 | 0.001327 |
| GO:0005161 | platelet-derived growth factor receptor binding | Molecular Function | 2 | 1 | 0.001341 |
| GO:0086008 | voltage-gated potassium channel activity involved in cardiac muscle cell action potential repolarization | Molecular Function | 1 | 2 | 0.001341 |
| GO:0010464 | regulation of mesenchymal cell proliferation | Biological Process | 3 | 1 | 0.001348 |
| GO:0060603 | mammary gland duct morphogenesis | Biological Process | 3 | 1 | 0.001348 |
| GO:0021773 | striatal medium spiny neuron differentiation | Biological Process | 1 | 1 | 0.001357 |
| GO:0042701 | progesterone secretion | Biological Process | 1 | 1 | 0.001357 |
| GO:0060319 | primitive erythrocyte differentiation | Biological Process | 1 | 1 | 0.001357 |
| GO:0071409 | cellular response to cycloheximide | Biological Process | 2 | 0 | 0.001357 |
| GO:0097068 | response to thyroxine | Biological Process | 2 | 0 | 0.001357 |
| GO:1990410 | adrenomedullin receptor signaling pathway | Biological Process | 2 | 0 | 0.001357 |
| GO:2000182 | regulation of progesterone biosynthetic process | Biological Process | 2 | 0 | 0.001357 |
| GO:0048638 | regulation of developmental growth | Biological Process | 10 | 3 | 0.00137 |
| GO:0055006 | cardiac cell development | Biological Process | 4 | 2 | 0.001377 |
| GO:0098915 | membrane repolarization during ventricular cardiac muscle cell action potential | Biological Process | 1 | 2 | 0.001385 |
| GO:0003013 | circulatory system process | Biological Process | 14 | 4 | 0.001403 |
| GO:0001818 | negative regulation of cytokine production | Biological Process | 12 | 0 | 0.001418 |
| GO:0030879 | mammary gland development | Biological Process | 6 | 2 | 0.001438 |
| GO:0043010 | camera-type eye development | Biological Process | 7 | 6 | 0.001448 |
| GO:1902807 | negative regulation of cell cycle G1/S phase transition | Biological Process | 5 | 2 | 0.001468 |
| GO:0090183 | regulation of kidney development | Biological Process | 5 | 0 | 0.00147 |
| GO:0071156 | regulation of cell cycle arrest | Biological Process | 7 | 0 | 0.001547 |
| GO:1902105 | regulation of leukocyte differentiation | Biological Process | 10 | 2 | 0.001549 |
| GO:1903706 | regulation of hemopoiesis | Biological Process | 15 | 2 | 0.001551 |
| GO:0003007 | heart morphogenesis | Biological Process | 7 | 4 | 0.001563 |
| GO:0032526 | response to retinoic acid | Biological Process | 4 | 3 | 0.001629 |
| GO:0005005 | transmembrane-ephrin receptor activity | Molecular Function | 1 | 2 | 0.001632 |
| GO:0071774 | response to fibroblast growth factor | Biological Process | 6 | 2 | 0.001643 |
| GO:0031331 | positive regulation of cellular catabolic process | Biological Process | 14 | 1 | 0.001646 |
| GO:0001892 | embryonic placenta development | Biological Process | 4 | 2 | 0.001665 |
| GO:0014706 | striated muscle tissue development | Biological Process | 12 | 2 | 0.001672 |
| GO:1903587 | regulation of blood vessel endothelial cell proliferation involved in sprouting angiogenesis | Biological Process | 2 | 1 | 0.001685 |
| GO:0032649 | regulation of interferon-gamma production | Biological Process | 6 | 1 | 0.001714 |
| GO:0007167 | enzyme linked receptor protein signaling pathway | Biological Process | 19 | 10 | 0.001715 |
| GO:0030324 | lung development | Biological Process | 7 | 2 | 0.001717 |
| GO:0006977 | DNA damage response, signal transduction by p53 class mediator resulting in cell cycle arrest | Biological Process | 5 | 0 | 0.001726 |
| GO:0043542 | endothelial cell migration | Biological Process | 8 | 2 | 0.001732 |
| GO:0016773 | phosphotransferase activity, alcohol group as acceptor | Molecular Function | 12 | 9 | 0.001769 |
| GO:0005730 | nucleolus | Cellular Component | 23 | 3 | 0.001787 |
| GO:0032609 | interferon-gamma production | Biological Process | 6 | 1 | 0.001803 |
| GO:0007169 | transmembrane receptor protein tyrosine kinase signaling pathway | Biological Process | 15 | 7 | 0.001821 |
| GO:0034641 | cellular nitrogen compound metabolic process | Biological Process | 100 | 26 | 0.001865 |
| GO:0072431 | signal transduction involved in mitotic G1 DNA damage checkpoint | Biological Process | 5 | 0 | 0.001866 |
| GO:1902400 | intracellular signal transduction involved in G1 DNA damage checkpoint | Biological Process | 5 | 0 | 0.001866 |
| GO:1904705 | regulation of vascular smooth muscle cell proliferation | Biological Process | 5 | 0 | 0.001866 |
| GO:1990874 | vascular smooth muscle cell proliferation | Biological Process | 5 | 0 | 0.001866 |
| GO:0019827 | stem cell population maintenance | Biological Process | 4 | 4 | 0.001872 |
| GO:0045637 | regulation of myeloid cell differentiation | Biological Process | 9 | 2 | 0.001885 |
| GO:0031625 | ubiquitin protein ligase binding | Molecular Function | 9 | 3 | 0.001939 |
| GO:0001654 | eye development | Biological Process | 7 | 7 | 0.00194 |
| GO:0044843 | cell cycle G1/S phase transition | Biological Process | 8 | 3 | 0.001944 |
| GO:0034620 | cellular response to unfolded protein | Biological Process | 8 | 0 | 0.001953 |
| GO:0097159 | organic cyclic compound binding | Molecular Function | 87 | 27 | 0.001975 |
| GO:0052548 | regulation of endopeptidase activity | Biological Process | 13 | 2 | 0.001984 |
| GO:0018108 | peptidyl-tyrosine phosphorylation | Biological Process | 10 | 4 | 0.001988 |
| GO:0046660 | female sex differentiation | Biological Process | 6 | 1 | 0.001992 |
| GO:0030323 | respiratory tube development | Biological Process | 7 | 2 | 0.001997 |
| GO:0005102 | signaling receptor binding | Molecular Function | 30 | 10 | 0.002003 |
| GO:0043406 | positive regulation of MAP kinase activity | Biological Process | 10 | 1 | 0.002004 |
| GO:1902402 | signal transduction involved in mitotic DNA damage checkpoint | Biological Process | 5 | 0 | 0.002014 |
| GO:1902403 | signal transduction involved in mitotic DNA integrity checkpoint | Biological Process | 5 | 0 | 0.002014 |
| GO:0048569 | post-embryonic animal organ development | Biological Process | 2 | 1 | 0.002023 |
| GO:0071371 | cellular response to gonadotropin stimulus | Biological Process | 3 | 0 | 0.002023 |
| GO:1904754 | positive regulation of vascular associated smooth muscle cell migration | Biological Process | 2 | 1 | 0.002023 |
| GO:1903439 | calcitonin family receptor complex | Cellular Component | 2 | 0 | 0.002074 |
| GO:0016301 | kinase activity | Molecular Function | 12 | 11 | 0.002091 |
| GO:0051385 | response to mineralocorticoid | Biological Process | 4 | 0 | 0.002106 |
| GO:0060021 | roof of mouth development | Biological Process | 3 | 3 | 0.002119 |
| GO:0060419 | heart growth | Biological Process | 5 | 1 | 0.002119 |
| GO:0098727 | maintenance of cell number | Biological Process | 4 | 4 | 0.002125 |
| GO:0018212 | peptidyl-tyrosine modification | Biological Process | 10 | 4 | 0.002138 |
| GO:0150063 | visual system development | Biological Process | 7 | 7 | 0.002138 |
| GO:0021879 | forebrain neuron differentiation | Biological Process | 2 | 3 | 0.00217 |
| GO:0005432 | calcium:sodium antiporter activity | Molecular Function | 1 | 1 | 0.00219 |
| GO:0015190 | L-leucine transmembrane transporter activity | Molecular Function | 2 | 0 | 0.00219 |
| GO:0045569 | TRAIL binding | Molecular Function | 2 | 0 | 0.00219 |
| GO:0071417 | cellular response to organonitrogen compound | Biological Process | 17 | 2 | 0.002211 |
| GO:0043229 | intracellular organelle | Cellular Component | 154 | 59 | 0.002218 |
| GO:0015827 | tryptophan transport | Biological Process | 2 | 0 | 0.002238 |
| GO:0030222 | eosinophil differentiation | Biological Process | 1 | 1 | 0.002238 |
| GO:0060426 | lung vasculature development | Biological Process | 2 | 0 | 0.002238 |
| GO:0098713 | leucine import across plasma membrane | Biological Process | 2 | 0 | 0.002238 |
| GO:1901490 | regulation of lymphangiogenesis | Biological Process | 2 | 0 | 0.002238 |
| GO:1903801 | L-leucine import across plasma membrane | Biological Process | 2 | 0 | 0.002238 |
| GO:1904386 | response to L-phenylalanine derivative | Biological Process | 2 | 0 | 0.002238 |
| GO:1905664 | regulation of calcium ion import across plasma membrane | Biological Process | 2 | 0 | 0.002238 |
| GO:0110110 | positive regulation of animal organ morphogenesis | Biological Process | 4 | 2 | 0.002245 |
| GO:0034605 | cellular response to heat | Biological Process | 7 | 0 | 0.002303 |
| GO:0035019 | somatic stem cell population maintenance | Biological Process | 2 | 3 | 0.002335 |
| GO:0072413 | signal transduction involved in mitotic cell cycle checkpoint | Biological Process | 5 | 0 | 0.002335 |
| GO:0045638 | negative regulation of myeloid cell differentiation | Biological Process | 4 | 2 | 0.002377 |
| GO:0030308 | negative regulation of cell growth | Biological Process | 7 | 2 | 0.002397 |
| GO:0007250 | activation of NF-kappaB-inducing kinase activity | Biological Process | 3 | 0 | 0.002401 |
| GO:0021756 | striatum development | Biological Process | 2 | 1 | 0.002401 |
| GO:0051447 | negative regulation of meiotic cell cycle | Biological Process | 3 | 0 | 0.002401 |
| GO:1903707 | negative regulation of hemopoiesis | Biological Process | 6 | 2 | 0.002404 |
| GO:0048880 | sensory system development | Biological Process | 7 | 7 | 0.002466 |
| GO:1902475 | L-alpha-amino acid transmembrane transport | Biological Process | 5 | 0 | 0.002509 |
| GO:0001776 | leukocyte homeostasis | Biological Process | 5 | 1 | 0.002515 |
| GO:0009607 | response to biotic stimulus | Biological Process | 22 | 7 | 0.002545 |
| GO:0032965 | regulation of collagen biosynthetic process | Biological Process | 4 | 0 | 0.002577 |
| GO:0090049 | regulation of cell migration involved in sprouting angiogenesis | Biological Process | 3 | 1 | 0.002577 |
| GO:0002260 | lymphocyte homeostasis | Biological Process | 4 | 1 | 0.002692 |
| GO:0031974 | membrane-enclosed lumen | Cellular Component | 82 | 22 | 0.002705 |
| GO:0043233 | organelle lumen | Cellular Component | 82 | 22 | 0.002705 |
| GO:0070013 | intracellular organelle lumen | Cellular Component | 82 | 22 | 0.002705 |
| GO:0048511 | rhythmic process | Biological Process | 10 | 2 | 0.002705 |
| GO:0051726 | regulation of cell cycle | Biological Process | 26 | 5 | 0.002725 |
| GO:0005003 | ephrin receptor activity | Molecular Function | 1 | 2 | 0.002732 |
| GO:0008015 | blood circulation | Biological Process | 13 | 4 | 0.002755 |
| GO:0009896 | positive regulation of catabolic process | Biological Process | 15 | 1 | 0.002785 |
| GO:0060602 | branch elongation of an epithelium | Biological Process | 1 | 2 | 0.002819 |
| GO:1903204 | negative regulation of oxidative stress-induced neuron death | Biological Process | 3 | 0 | 0.002819 |
| GO:0032872 | regulation of stress-activated MAPK cascade | Biological Process | 8 | 2 | 0.002831 |
| GO:0045746 | negative regulation of Notch signaling pathway | Biological Process | 2 | 2 | 0.002838 |
| GO:1900744 | regulation of p38MAPK cascade | Biological Process | 4 | 0 | 0.002838 |
| GO:1902042 | negative regulation of extrinsic apoptotic signaling pathway via death domain receptors | Biological Process | 4 | 0 | 0.002838 |
| GO:1904018 | positive regulation of vasculature development | Biological Process | 7 | 2 | 0.002859 |
| GO:0002720 | positive regulation of cytokine production involved in immune response | Biological Process | 4 | 1 | 0.002884 |
| GO:0009986 | cell surface | Cellular Component | 16 | 8 | 0.002887 |
| GO:2001234 | negative regulation of apoptotic signaling pathway | Biological Process | 9 | 1 | 0.00292 |
| GO:0001657 | ureteric bud development | Biological Process | 4 | 2 | 0.002966 |
| GO:0010035 | response to inorganic substance | Biological Process | 17 | 1 | 0.003011 |
| GO:0009615 | response to virus | Biological Process | 7 | 6 | 0.003058 |
| GO:0001763 | morphogenesis of a branching structure | Biological Process | 6 | 3 | 0.003062 |
| GO:0060420 | regulation of heart growth | Biological Process | 4 | 1 | 0.003087 |
| GO:0051051 | negative regulation of transport | Biological Process | 12 | 3 | 0.003088 |
| GO:1901653 | cellular response to peptide | Biological Process | 13 | 1 | 0.003105 |
| GO:0070302 | regulation of stress-activated protein kinase signaling cascade | Biological Process | 8 | 2 | 0.003106 |
| GO:0014911 | positive regulation of smooth muscle cell migration | Biological Process | 3 | 1 | 0.003116 |
| GO:1902895 | positive regulation of pri-miRNA transcription by RNA polymerase II | Biological Process | 3 | 1 | 0.003116 |
| GO:0002367 | cytokine production involved in immune response | Biological Process | 5 | 1 | 0.003128 |
| GO:0002718 | regulation of cytokine production involved in immune response | Biological Process | 5 | 1 | 0.003128 |
| GO:0072163 | mesonephric epithelium development | Biological Process | 4 | 2 | 0.003128 |
| GO:0072164 | mesonephric tubule development | Biological Process | 4 | 2 | 0.003128 |
| GO:1901699 | cellular response to nitrogen compound | Biological Process | 17 | 3 | 0.003183 |
| GO:0097642 | calcitonin family receptor activity | Molecular Function | 2 | 0 | 0.003252 |
| GO:0006534 | cysteine metabolic process | Biological Process | 3 | 0 | 0.00328 |
| GO:0044346 | fibroblast apoptotic process | Biological Process | 3 | 0 | 0.00328 |
| GO:0072111 | cell proliferation involved in kidney development | Biological Process | 3 | 0 | 0.00328 |
| GO:1902107 | positive regulation of leukocyte differentiation | Biological Process | 8 | 0 | 0.00329 |
| GO:0000079 | regulation of cyclin-dependent protein serine/threonine kinase activity | Biological Process | 5 | 1 | 0.003298 |
| GO:0031571 | mitotic G1 DNA damage checkpoint | Biological Process | 5 | 0 | 0.003299 |
| GO:0046898 | response to cycloheximide | Biological Process | 2 | 0 | 0.003324 |
| GO:0072126 | positive regulation of glomerular mesangial cell proliferation | Biological Process | 2 | 0 | 0.003324 |
| GO:2000489 | regulation of hepatic stellate cell activation | Biological Process | 2 | 0 | 0.003324 |
| GO:0051591 | response to cAMP | Biological Process | 5 | 1 | 0.003474 |
| GO:1901216 | positive regulation of neuron death | Biological Process | 5 | 1 | 0.003474 |
| GO:1905897 | regulation of response to endoplasmic reticulum stress | Biological Process | 5 | 1 | 0.003474 |
| GO:0098772 | molecular function regulator | Molecular Function | 33 | 10 | 0.003477 |
| GO:0015893 | drug transport | Biological Process | 8 | 2 | 0.003505 |
| GO:0044783 | G1 DNA damage checkpoint | Biological Process | 5 | 0 | 0.003522 |
| GO:0044819 | mitotic G1/S transition checkpoint | Biological Process | 5 | 0 | 0.003522 |
| GO:0052547 | regulation of peptidase activity | Biological Process | 13 | 2 | 0.003577 |
| GO:0008138 | protein tyrosine/serine/threonine phosphatase activity | Molecular Function | 4 | 0 | 0.003583 |
| GO:0030278 | regulation of ossification | Biological Process | 4 | 5 | 0.003741 |
| GO:0014910 | regulation of smooth muscle cell migration | Biological Process | 4 | 1 | 0.003755 |
| GO:0042476 | odontogenesis | Biological Process | 5 | 2 | 0.003774 |
| GO:0072215 | regulation of metanephros development | Biological Process | 3 | 0 | 0.003784 |
| GO:0090050 | positive regulation of cell migration involved in sprouting angiogenesis | Biological Process | 2 | 1 | 0.003784 |
| GO:0098703 | calcium ion import across plasma membrane | Biological Process | 2 | 1 | 0.003784 |
| GO:0004860 | protein kinase inhibitor activity | Molecular Function | 4 | 1 | 0.003817 |
| GO:0001823 | mesonephros development | Biological Process | 4 | 2 | 0.003846 |
| GO:0001046 | core promoter sequence-specific DNA binding | Molecular Function | 4 | 0 | 0.003905 |
| GO:0001047 | core promoter binding | Molecular Function | 4 | 0 | 0.003905 |
| GO:0043433 | negative regulation of DNA-binding transcription factor activity | Biological Process | 8 | 0 | 0.00396 |
| GO:1901654 | response to ketone | Biological Process | 8 | 1 | 0.003991 |
| GO:0071230 | cellular response to amino acid stimulus | Biological Process | 5 | 0 | 0.003999 |
| GO:0071300 | cellular response to retinoic acid | Biological Process | 2 | 3 | 0.003999 |
| GO:0097327 | response to antineoplastic agent | Biological Process | 6 | 0 | 0.004043 |
| GO:1904029 | regulation of cyclin-dependent protein kinase activity | Biological Process | 5 | 1 | 0.004043 |
| GO:0010712 | regulation of collagen metabolic process | Biological Process | 4 | 0 | 0.004061 |
| GO:0032570 | response to progesterone | Biological Process | 3 | 1 | 0.004061 |
| GO:0050680 | negative regulation of epithelial cell proliferation | Biological Process | 1 | 6 | 0.004104 |
| GO:0051128 | regulation of cellular component organization | Biological Process | 41 | 16 | 0.004122 |
| GO:0051402 | neuron apoptotic process | Biological Process | 9 | 1 | 0.00418 |
| GO:0051098 | regulation of binding | Biological Process | 11 | 2 | 0.004243 |
| GO:0002027 | regulation of heart rate | Biological Process | 4 | 2 | 0.004248 |
| GO:0001707 | mesoderm formation | Biological Process | 4 | 1 | 0.004254 |
| GO:0003281 | ventricular septum development | Biological Process | 2 | 3 | 0.004254 |
| GO:0042698 | ovulation cycle | Biological Process | 4 | 1 | 0.004254 |
| GO:0010594 | regulation of endothelial cell migration | Biological Process | 7 | 1 | 0.004257 |
| GO:0038037 | G protein-coupled receptor dimeric complex | Cellular Component | 1 | 1 | 0.004272 |
| GO:0035970 | peptidyl-threonine dephosphorylation | Biological Process | 3 | 0 | 0.004332 |
| GO:0036303 | lymph vessel morphogenesis | Biological Process | 3 | 0 | 0.004332 |
| GO:0036499 | PERK-mediated unfolded protein response | Biological Process | 3 | 0 | 0.004332 |
| GO:0060343 | trabecula formation | Biological Process | 2 | 1 | 0.004332 |
| GO:1902656 | calcium ion import into cytosol | Biological Process | 2 | 1 | 0.004332 |
| GO:0060541 | respiratory system development | Biological Process | 7 | 2 | 0.004392 |
| GO:0035967 | cellular response to topologically incorrect protein | Biological Process | 8 | 0 | 0.004411 |
| GO:0060485 | mesenchyme development | Biological Process | 6 | 5 | 0.004412 |
| GO:0010463 | mesenchymal cell proliferation | Biological Process | 3 | 1 | 0.004414 |
| GO:0045646 | regulation of erythrocyte differentiation | Biological Process | 2 | 2 | 0.004414 |
| GO:0036293 | response to decreased oxygen levels | Biological Process | 12 | 1 | 0.004439 |
| GO:1901343 | negative regulation of vasculature development | Biological Process | 7 | 0 | 0.004456 |
| GO:1902106 | negative regulation of leukocyte differentiation | Biological Process | 4 | 2 | 0.00446 |
| GO:0005004 | GPI-linked ephrin receptor activity | Molecular Function | 0 | 2 | 0.004507 |
| GO:0015658 | branched-chain amino acid transmembrane transporter activity | Molecular Function | 2 | 0 | 0.004507 |
| GO:0006139 | nucleobase-containing compound metabolic process | Biological Process | 91 | 21 | 0.004521 |
| GO:0045926 | negative regulation of growth | Biological Process | 8 | 2 | 0.004554 |
| GO:0003149 | membranous septum morphogenesis | Biological Process | 0 | 2 | 0.004607 |
| GO:0021902 | commitment of neuronal cell to specific neuron type in forebrain | Biological Process | 0 | 2 | 0.004607 |
| GO:0031077 | post-embryonic camera-type eye development | Biological Process | 1 | 1 | 0.004607 |
| GO:0032808 | lacrimal gland development | Biological Process | 1 | 1 | 0.004607 |
| GO:0045650 | negative regulation of macrophage differentiation | Biological Process | 1 | 1 | 0.004607 |
| GO:0045656 | negative regulation of monocyte differentiation | Biological Process | 1 | 1 | 0.004607 |
| GO:0045906 | negative regulation of vasoconstriction | Biological Process | 1 | 1 | 0.004607 |
| GO:0060022 | hard palate development | Biological Process | 0 | 2 | 0.004607 |
| GO:0060449 | bud elongation involved in lung branching | Biological Process | 0 | 2 | 0.004607 |
| GO:0060462 | lung lobe development | Biological Process | 1 | 1 | 0.004607 |
| GO:0060463 | lung lobe morphogenesis | Biological Process | 1 | 1 | 0.004607 |
| GO:1901165 | positive regulation of trophoblast cell migration | Biological Process | 2 | 0 | 0.004607 |
| GO:0001667 | ameboidal-type cell migration | Biological Process | 10 | 4 | 0.004691 |
| GO:0003197 | endocardial cushion development | Biological Process | 3 | 1 | 0.004788 |
| GO:0015804 | neutral amino acid transport | Biological Process | 4 | 0 | 0.004788 |
| GO:0060443 | mammary gland morphogenesis | Biological Process | 3 | 1 | 0.004788 |
| GO:0015807 | L-amino acid transport | Biological Process | 5 | 0 | 0.004799 |
| GO:0021872 | forebrain generation of neurons | Biological Process | 2 | 3 | 0.004799 |
| GO:0048332 | mesoderm morphogenesis | Biological Process | 4 | 1 | 0.004799 |
| GO:0060562 | epithelial tube morphogenesis | Biological Process | 7 | 5 | 0.004817 |
| GO:0043280 | positive regulation of cysteine-type endopeptidase activity involved in apoptotic process | Biological Process | 7 | 0 | 0.00483 |
| GO:0019210 | kinase inhibitor activity | Molecular Function | 4 | 1 | 0.00486 |
| GO:0032868 | response to insulin | Biological Process | 10 | 1 | 0.004892 |
| GO:0072331 | signal transduction by p53 class mediator | Biological Process | 10 | 1 | 0.004892 |
| GO:0001819 | positive regulation of cytokine production | Biological Process | 15 | 1 | 0.00491 |
| GO:0006935 | chemotaxis | Biological Process | 13 | 6 | 0.004918 |
| GO:0006379 | mRNA cleavage | Biological Process | 3 | 0 | 0.004927 |
| GO:0032682 | negative regulation of chemokine production | Biological Process | 3 | 0 | 0.004927 |
| GO:0044342 | type B pancreatic cell proliferation | Biological Process | 3 | 0 | 0.004927 |
| GO:0060444 | branching involved in mammary gland duct morphogenesis | Biological Process | 3 | 0 | 0.004927 |
| GO:0086013 | membrane repolarization during cardiac muscle cell action potential | Biological Process | 1 | 2 | 0.004927 |
| GO:0048469 | cell maturation | Biological Process | 6 | 2 | 0.005071 |
| GO:0055001 | muscle cell development | Biological Process | 6 | 2 | 0.005071 |
| GO:0031670 | cellular response to nutrient | Biological Process | 3 | 2 | 0.005089 |
| GO:0051881 | regulation of mitochondrial membrane potential | Biological Process | 5 | 0 | 0.005089 |
| GO:0072401 | signal transduction involved in DNA integrity checkpoint | Biological Process | 5 | 0 | 0.005089 |
| GO:0072422 | signal transduction involved in DNA damage checkpoint | Biological Process | 5 | 0 | 0.005089 |
| GO:0043523 | regulation of neuron apoptotic process | Biological Process | 8 | 1 | 0.005128 |
| GO:0042330 | taxis | Biological Process | 13 | 6 | 0.005163 |
| GO:0001558 | regulation of cell growth | Biological Process | 12 | 2 | 0.005203 |
| GO:0048666 | neuron development | Biological Process | 15 | 14 | 0.005273 |
| GO:0048644 | muscle organ morphogenesis | Biological Process | 3 | 2 | 0.005391 |
| GO:0008327 | methyl-CpG binding | Molecular Function | 2 | 1 | 0.005399 |
| GO:0045862 | positive regulation of proteolysis | Biological Process | 12 | 1 | 0.005414 |
| GO:0019838 | growth factor binding | Molecular Function | 4 | 3 | 0.005536 |
| GO:0010631 | epithelial cell migration | Biological Process | 9 | 2 | 0.005549 |
| GO:0001783 | B cell apoptotic process | Biological Process | 3 | 0 | 0.005568 |
| GO:0021544 | subpallium development | Biological Process | 2 | 1 | 0.005568 |
| GO:0060045 | positive regulation of cardiac muscle cell proliferation | Biological Process | 2 | 1 | 0.005568 |
| GO:0032964 | collagen biosynthetic process | Biological Process | 4 | 0 | 0.005598 |
| GO:0042059 | negative regulation of epidermal growth factor receptor signaling pathway | Biological Process | 3 | 1 | 0.005598 |
| GO:0007254 | JNK cascade | Biological Process | 7 | 2 | 0.005614 |
| GO:1990316 | Atg1/ULK1 kinase complex | Cellular Component | 2 | 0 | 0.005641 |
| GO:0002009 | morphogenesis of an epithelium | Biological Process | 11 | 6 | 0.005648 |
| GO:0006725 | cellular aromatic compound metabolic process | Biological Process | 93 | 22 | 0.005669 |
| GO:1901564 | organonitrogen compound metabolic process | Biological Process | 95 | 35 | 0.005706 |
| GO:0055013 | cardiac muscle cell development | Biological Process | 4 | 1 | 0.005706 |
| GO:0070227 | lymphocyte apoptotic process | Biological Process | 5 | 0 | 0.005706 |
| GO:1903670 | regulation of sprouting angiogenesis | Biological Process | 4 | 1 | 0.005706 |
| GO:0022414 | reproductive process | Biological Process | 29 | 6 | 0.005721 |
| GO:0019900 | kinase binding | Molecular Function | 17 | 4 | 0.00577 |
| GO:0070888 | E-box binding | Molecular Function | 3 | 1 | 0.005806 |
| GO:0000003 | reproduction | Biological Process | 29 | 6 | 0.005901 |
| GO:0071902 | positive regulation of protein serine/threonine kinase activity | Biological Process | 11 | 1 | 0.005937 |
| GO:0090132 | epithelium migration | Biological Process | 9 | 2 | 0.005975 |
| GO:0014909 | smooth muscle cell migration | Biological Process | 4 | 1 | 0.006034 |
| GO:0072395 | signal transduction involved in cell cycle checkpoint | Biological Process | 5 | 0 | 0.006034 |
| GO:0042149 | cellular response to glucose starvation | Biological Process | 4 | 0 | 0.006035 |
| GO:0090199 | regulation of release of cytochrome c from mitochondria | Biological Process | 4 | 0 | 0.006035 |
| GO:0019207 | kinase regulator activity | Molecular Function | 7 | 2 | 0.00607 |
| GO:0071214 | cellular response to abiotic stimulus | Biological Process | 12 | 0 | 0.006073 |
| GO:0104004 | cellular response to environmental stimulus | Biological Process | 12 | 0 | 0.006073 |
| GO:0006701 | progesterone biosynthetic process | Biological Process | 2 | 0 | 0.006081 |
| GO:0015820 | leucine transport | Biological Process | 2 | 0 | 0.006081 |
| GO:0021877 | forebrain neuron fate commitment | Biological Process | 0 | 2 | 0.006081 |
| GO:0060215 | primitive hemopoiesis | Biological Process | 1 | 1 | 0.006081 |
| GO:0060947 | cardiac vascular smooth muscle cell differentiation | Biological Process | 2 | 0 | 0.006081 |
| GO:2000271 | positive regulation of fibroblast apoptotic process | Biological Process | 2 | 0 | 0.006081 |
| GO:2000766 | negative regulation of cytoplasmic translation | Biological Process | 2 | 0 | 0.006081 |
| GO:0030098 | lymphocyte differentiation | Biological Process | 10 | 3 | 0.006154 |
| GO:0071887 | leukocyte apoptotic process | Biological Process | 6 | 0 | 0.006169 |
| GO:0010950 | positive regulation of endopeptidase activity | Biological Process | 8 | 0 | 0.006201 |
| GO:0051897 | positive regulation of protein kinase B signaling | Biological Process | 7 | 1 | 0.006201 |
| GO:0002021 | response to dietary excess | Biological Process | 3 | 0 | 0.006257 |
| GO:0043555 | regulation of translation in response to stress | Biological Process | 3 | 0 | 0.006257 |
| GO:0034976 | response to endoplasmic reticulum stress | Biological Process | 10 | 1 | 0.006274 |
| GO:0001649 | osteoblast differentiation | Biological Process | 4 | 5 | 0.006316 |
| GO:0072006 | nephron development | Biological Process | 6 | 1 | 0.006328 |
| GO:0072332 | intrinsic apoptotic signaling pathway by p53 class mediator | Biological Process | 5 | 0 | 0.006375 |
| GO:0008544 | epidermis development | Biological Process | 12 | 3 | 0.006449 |
| GO:0001775 | cell activation | Biological Process | 29 | 6 | 0.006471 |
| GO:0042398 | cellular modified amino acid biosynthetic process | Biological Process | 4 | 0 | 0.006494 |
| GO:0060038 | cardiac muscle cell proliferation | Biological Process | 3 | 1 | 0.006494 |
| GO:0061138 | morphogenesis of a branching epithelium | Biological Process | 6 | 2 | 0.006617 |
| GO:0043226 | organelle | Cellular Component | 158 | 63 | 0.006747 |
| GO:0002684 | positive regulation of immune system process | Biological Process | 27 | 3 | 0.006774 |
| GO:0010948 | negative regulation of cell cycle process | Biological Process | 10 | 2 | 0.006792 |
| GO:0090130 | tissue migration | Biological Process | 9 | 2 | 0.006906 |
| GO:1902893 | regulation of pri-miRNA transcription by RNA polymerase II | Biological Process | 3 | 1 | 0.006976 |
| GO:2000677 | regulation of transcription regulatory region DNA binding | Biological Process | 4 | 0 | 0.006976 |
| GO:0050927 | positive regulation of positive chemotaxis | Biological Process | 3 | 0 | 0.006994 |
| GO:0061082 | myeloid leukocyte cytokine production | Biological Process | 3 | 0 | 0.006994 |
| GO:0086011 | membrane repolarization during action potential | Biological Process | 1 | 2 | 0.006994 |
| GO:0099625 | ventricular cardiac muscle cell membrane repolarization | Biological Process | 1 | 2 | 0.006994 |
| GO:1903203 | regulation of oxidative stress-induced neuron death | Biological Process | 3 | 0 | 0.006994 |
| GO:0019901 | protein kinase binding | Molecular Function | 15 | 4 | 0.007039 |
| GO:0006865 | amino acid transport | Biological Process | 7 | 0 | 0.007066 |
| GO:0031100 | animal organ regeneration | Biological Process | 5 | 0 | 0.007096 |
| GO:0055017 | cardiac muscle tissue growth | Biological Process | 4 | 1 | 0.007096 |
| GO:0043900 | regulation of multi-organism process | Biological Process | 12 | 2 | 0.007149 |
| GO:0070578 | RISC-loading complex | Cellular Component | 2 | 0 | 0.007183 |
| GO:0002064 | epithelial cell development | Biological Process | 7 | 2 | 0.007287 |
| GO:2000278 | regulation of DNA biosynthetic process | Biological Process | 6 | 0 | 0.007337 |
| GO:0004672 | protein kinase activity | Molecular Function | 11 | 6 | 0.007365 |
| GO:0051607 | defense response to virus | Biological Process | 6 | 4 | 0.007383 |
| GO:0007492 | endoderm development | Biological Process | 4 | 1 | 0.007477 |
| GO:0050873 | brown fat cell differentiation | Biological Process | 2 | 2 | 0.007481 |
| GO:0061614 | pri-miRNA transcription by RNA polymerase II | Biological Process | 3 | 1 | 0.007481 |
| GO:0046649 | lymphocyte activation | Biological Process | 18 | 3 | 0.007588 |
| GO:0008406 | gonad development | Biological Process | 8 | 1 | 0.007706 |
| GO:0002315 | marginal zone B cell differentiation | Biological Process | 0 | 2 | 0.00774 |
| GO:0009629 | response to gravity | Biological Process | 2 | 0 | 0.00774 |
| GO:0010533 | regulation of activation of Janus kinase activity | Biological Process | 2 | 0 | 0.00774 |
| GO:0044557 | relaxation of smooth muscle | Biological Process | 1 | 1 | 0.00774 |
| GO:0050847 | progesterone receptor signaling pathway | Biological Process | 1 | 1 | 0.00774 |
| GO:0072124 | regulation of glomerular mesangial cell proliferation | Biological Process | 2 | 0 | 0.00774 |
| GO:0016049 | cell growth | Biological Process | 13 | 2 | 0.007741 |
| GO:0048863 | stem cell differentiation | Biological Process | 6 | 4 | 0.007767 |
| GO:0001945 | lymph vessel development | Biological Process | 3 | 0 | 0.007781 |
| GO:0035886 | vascular smooth muscle cell differentiation | Biological Process | 3 | 0 | 0.007781 |
| GO:0046885 | regulation of hormone biosynthetic process | Biological Process | 3 | 0 | 0.007781 |
| GO:0050926 | regulation of positive chemotaxis | Biological Process | 3 | 0 | 0.007781 |
| GO:0060259 | regulation of feeding behavior | Biological Process | 2 | 1 | 0.007781 |
| GO:1900745 | positive regulation of p38MAPK cascade | Biological Process | 3 | 0 | 0.007781 |
| GO:1904752 | regulation of vascular associated smooth muscle cell migration | Biological Process | 2 | 1 | 0.007781 |
| GO:0009064 | glutamine family amino acid metabolic process | Biological Process | 5 | 0 | 0.007873 |
| GO:0048145 | regulation of fibroblast proliferation | Biological Process | 4 | 1 | 0.007873 |
| GO:0007399 | nervous system development | Biological Process | 30 | 22 | 0.00798 |
| GO:0007566 | embryo implantation | Biological Process | 4 | 0 | 0.00801 |
| GO:0010596 | negative regulation of endothelial cell migration | Biological Process | 4 | 0 | 0.00801 |
| GO:1901185 | negative regulation of ERBB signaling pathway | Biological Process | 3 | 1 | 0.00801 |
| GO:0001837 | epithelial to mesenchymal transition | Biological Process | 3 | 4 | 0.008147 |
| GO:0035148 | tube formation | Biological Process | 4 | 3 | 0.008147 |
| GO:0022008 | neurogenesis | Biological Process | 22 | 16 | 0.008233 |
| GO:0001666 | response to hypoxia | Biological Process | 11 | 1 | 0.008253 |
| GO:0005737 | cytoplasm | Cellular Component | 134 | 60 | 0.008263 |
| GO:0048144 | fibroblast proliferation | Biological Process | 4 | 1 | 0.008282 |
| GO:0043200 | response to amino acid | Biological Process | 6 | 0 | 0.008312 |
| GO:0042110 | T cell activation | Biological Process | 13 | 2 | 0.008313 |
| GO:0001935 | endothelial cell proliferation | Biological Process | 6 | 1 | 0.008435 |
| GO:0048754 | branching morphogenesis of an epithelial tube | Biological Process | 5 | 2 | 0.008435 |
| GO:0062012 | regulation of small molecule metabolic process | Biological Process | 12 | 2 | 0.008477 |
| GO:0007219 | Notch signaling pathway | Biological Process | 4 | 4 | 0.008495 |
| GO:0005942 | phosphatidylinositol 3-kinase complex | Cellular Component | 1 | 2 | 0.00856 |
| GO:0006984 | ER-nucleus signaling pathway | Biological Process | 4 | 0 | 0.008562 |
| GO:0048699 | generation of neurons | Biological Process | 20 | 16 | 0.008593 |
| GO:0051345 | positive regulation of hydrolase activity | Biological Process | 16 | 5 | 0.008593 |
| GO:0010632 | regulation of epithelial cell migration | Biological Process | 8 | 1 | 0.008598 |
| GO:0035902 | response to immobilization stress | Biological Process | 2 | 1 | 0.008618 |
| GO:1904738 | vascular associated smooth muscle cell migration | Biological Process | 2 | 1 | 0.008618 |
| GO:0031623 | receptor internalization | Biological Process | 5 | 1 | 0.008657 |
| GO:0002088 | lens development in camera-type eye | Biological Process | 2 | 3 | 0.008707 |
| GO:1905039 | carboxylic acid transmembrane transport | Biological Process | 7 | 0 | 0.008731 |
| GO:2000045 | regulation of G1/S transition of mitotic cell cycle | Biological Process | 5 | 2 | 0.008731 |
| GO:0030522 | intracellular receptor signaling pathway | Biological Process | 8 | 4 | 0.008791 |
| GO:0050767 | regulation of neurogenesis | Biological Process | 11 | 11 | 0.008806 |
| GO:0016772 | transferase activity, transferring phosphorus-containing groups | Molecular Function | 13 | 11 | 0.008837 |
| GO:0051336 | regulation of hydrolase activity | Biological Process | 21 | 10 | 0.008961 |
| GO:2001056 | positive regulation of cysteine-type endopeptidase activity | Biological Process | 7 | 0 | 0.009035 |
| GO:0045596 | negative regulation of cell differentiation | Biological Process | 12 | 8 | 0.009052 |
| GO:0045137 | development of primary sexual characteristics | Biological Process | 8 | 1 | 0.009073 |
| GO:0007610 | behavior | Biological Process | 12 | 5 | 0.009116 |
| GO:0001706 | endoderm formation | Biological Process | 3 | 1 | 0.009139 |
| GO:0070231 | T cell apoptotic process | Biological Process | 4 | 0 | 0.009139 |
| GO:0071158 | positive regulation of cell cycle arrest | Biological Process | 5 | 0 | 0.009145 |
| GO:0002831 | regulation of response to biotic stimulus | Biological Process | 5 | 2 | 0.009346 |
| GO:1903825 | organic acid transmembrane transport | Biological Process | 7 | 0 | 0.009346 |
| GO:0015173 | aromatic amino acid transmembrane transporter activity | Molecular Function | 2 | 0 | 0.009375 |
| GO:0016303 | 1-phosphatidylinositol-3-kinase activity | Molecular Function | 0 | 2 | 0.009375 |
| GO:1902555 | endoribonuclease complex | Cellular Component | 3 | 0 | 0.009409 |
| GO:1902554 | serine/threonine protein kinase complex | Cellular Component | 4 | 1 | 0.00947 |
| GO:0003676 | nucleic acid binding | Molecular Function | 63 | 14 | 0.009501 |
| GO:0060674 | placenta blood vessel development | Biological Process | 2 | 1 | 0.009506 |
| GO:0050679 | positive regulation of epithelial cell proliferation | Biological Process | 6 | 2 | 0.009572 |
| GO:0001553 | luteinization | Biological Process | 1 | 1 | 0.009578 |
| GO:0048755 | branching morphogenesis of a nerve | Biological Process | 0 | 2 | 0.009578 |
| GO:0051712 | positive regulation of killing of cells of other organism | Biological Process | 2 | 0 | 0.009578 |
| GO:0070561 | vitamin D receptor signaling pathway | Biological Process | 1 | 1 | 0.009578 |
| GO:0072110 | glomerular mesangial cell proliferation | Biological Process | 2 | 0 | 0.009578 |
| GO:0072203 | cell proliferation involved in metanephros development | Biological Process | 2 | 0 | 0.009578 |
| GO:0090193 | positive regulation of glomerulus development | Biological Process | 2 | 0 | 0.009578 |
| GO:2000048 | negative regulation of cell-cell adhesion mediated by cadherin | Biological Process | 2 | 0 | 0.009578 |
| GO:2000252 | negative regulation of feeding behavior | Biological Process | 1 | 1 | 0.009578 |
| GO:0030162 | regulation of proteolysis | Biological Process | 18 | 4 | 0.009649 |
| GO:0001541 | ovarian follicle development | Biological Process | 4 | 0 | 0.00974 |
| GO:0010332 | response to gamma radiation | Biological Process | 4 | 0 | 0.00974 |
| GO:0046854 | phosphatidylinositol phosphorylation | Biological Process | 2 | 2 | 0.00974 |
| GO:0016525 | negative regulation of angiogenesis | Biological Process | 6 | 0 | 0.009753 |
| GO:0006974 | cellular response to DNA damage stimulus | Biological Process | 21 | 2 | 0.009912 |
| GO:0042692 | muscle cell differentiation | Biological Process | 9 | 3 | 0.00995 |
| GO:0015179 | L-amino acid transmembrane transporter activity | Molecular Function | 4 | 0 | 0.009984 |
| GO:0055002 | striated muscle cell development | Biological Process | 5 | 2 | 0.009991 |
| GO:0017147 | Wnt-protein binding | Molecular Function | 0 | 3 | 0.010136 |
| GO:0033612 | receptor serine/threonine kinase binding | Molecular Function | 3 | 0 | 0.010136 |
| GO:0034763 | negative regulation of transmembrane transport | Biological Process | 6 | 0 | 0.010139 |
| GO:0045667 | regulation of osteoblast differentiation | Biological Process | 2 | 4 | 0.010139 |
| GO:1901360 | organic cyclic compound metabolic process | Biological Process | 95 | 22 | 0.010172 |
| GO:0022610 | biological adhesion | Biological Process | 22 | 12 | 0.010215 |
| GO:0051707 | response to other organism | Biological Process | 19 | 7 | 0.010259 |
| GO:1905348 | endonuclease complex | Cellular Component | 3 | 0 | 0.010307 |
| GO:0043331 | response to dsRNA | Biological Process | 2 | 2 | 0.010367 |
| GO:0043207 | response to external biotic stimulus | Biological Process | 19 | 7 | 0.010377 |
| GO:0001782 | B cell homeostasis | Biological Process | 2 | 1 | 0.010446 |
| GO:0003401 | axis elongation | Biological Process | 1 | 2 | 0.010446 |
| GO:0007176 | regulation of epidermal growth factor-activated receptor activity | Biological Process | 3 | 0 | 0.010446 |
| GO:0036475 | neuron death in response to oxidative stress | Biological Process | 3 | 0 | 0.010446 |
| GO:0070229 | negative regulation of lymphocyte apoptotic process | Biological Process | 3 | 0 | 0.010446 |
| GO:0071480 | cellular response to gamma radiation | Biological Process | 3 | 0 | 0.010446 |
| GO:0003206 | cardiac chamber morphogenesis | Biological Process | 4 | 2 | 0.010536 |
| GO:0008637 | apoptotic mitochondrial changes | Biological Process | 6 | 0 | 0.010536 |
| GO:0007568 | aging | Biological Process | 8 | 3 | 0.010616 |
| GO:0010952 | positive regulation of peptidase activity | Biological Process | 8 | 0 | 0.010748 |
| GO:0003015 | heart process | Biological Process | 7 | 3 | 0.010898 |
| GO:0021700 | developmental maturation | Biological Process | 8 | 2 | 0.010898 |
| GO:2000181 | negative regulation of blood vessel morphogenesis | Biological Process | 6 | 0 | 0.010944 |
| GO:0043525 | positive regulation of neuron apoptotic process | Biological Process | 3 | 1 | 0.011019 |
| GO:0048008 | platelet-derived growth factor receptor signaling pathway | Biological Process | 4 | 0 | 0.011019 |
| GO:0007162 | negative regulation of cell adhesion | Biological Process | 8 | 2 | 0.011153 |
| GO:0016667 | oxidoreductase activity, acting on a sulfur group of donors | Molecular Function | 3 | 1 | 0.011268 |
| GO:1990841 | promoter-specific chromatin binding | Molecular Function | 4 | 0 | 0.011268 |
| GO:0015368 | calcium:cation antiporter activity | Molecular Function | 1 | 1 | 0.011346 |
| GO:0003231 | cardiac ventricle development | Biological Process | 3 | 3 | 0.011363 |
| GO:2001242 | regulation of intrinsic apoptotic signaling pathway | Biological Process | 6 | 1 | 0.011379 |
| GO:0002082 | regulation of oxidative phosphorylation | Biological Process | 3 | 0 | 0.011437 |
| GO:0051085 | chaperone cofactor-dependent protein refolding | Biological Process | 3 | 0 | 0.011437 |
| GO:0060317 | cardiac epithelial to mesenchymal transition | Biological Process | 1 | 2 | 0.011437 |
| GO:0071295 | cellular response to vitamin | Biological Process | 2 | 1 | 0.011437 |
| GO:1900101 | regulation of endoplasmic reticulum unfolded protein response | Biological Process | 3 | 0 | 0.011437 |
| GO:0014812 | muscle cell migration | Biological Process | 4 | 1 | 0.01157 |
| GO:0042058 | regulation of epidermal growth factor receptor signaling pathway | Biological Process | 4 | 1 | 0.01157 |
| GO:0002903 | negative regulation of B cell apoptotic process | Biological Process | 2 | 0 | 0.01159 |
| GO:0003334 | keratinocyte development | Biological Process | 1 | 1 | 0.01159 |
| GO:0036490 | regulation of translation in response to endoplasmic reticulum stress | Biological Process | 2 | 0 | 0.01159 |
| GO:0043619 | regulation of transcription from RNA polymerase II promoter in response to oxidative stress | Biological Process | 2 | 0 | 0.01159 |
| GO:0045654 | positive regulation of megakaryocyte differentiation | Biological Process | 1 | 1 | 0.01159 |
| GO:0046886 | positive regulation of hormone biosynthetic process | Biological Process | 2 | 0 | 0.01159 |
| GO:0055015 | ventricular cardiac muscle cell development | Biological Process | 2 | 0 | 0.01159 |
| GO:0061314 | Notch signaling involved in heart development | Biological Process | 1 | 1 | 0.01159 |
| GO:0086103 | G protein-coupled receptor signaling pathway involved in heart process | Biological Process | 2 | 0 | 0.01159 |
| GO:0090084 | negative regulation of inclusion body assembly | Biological Process | 2 | 0 | 0.01159 |
| GO:0007411 | axon guidance | Biological Process | 5 | 5 | 0.011678 |
| GO:1903522 | regulation of blood circulation | Biological Process | 7 | 3 | 0.011678 |
| GO:0055021 | regulation of cardiac muscle tissue growth | Biological Process | 3 | 1 | 0.011697 |
| GO:0035265 | organ growth | Biological Process | 6 | 1 | 0.011746 |
| GO:0005829 | cytosol | Cellular Component | 71 | 25 | 0.011935 |
| GO:0097485 | neuron projection guidance | Biological Process | 5 | 5 | 0.011947 |
| GO:0004714 | transmembrane receptor protein tyrosine kinase activity | Molecular Function | 3 | 3 | 0.012071 |
| GO:0042475 | odontogenesis of dentin-containing tooth | Biological Process | 4 | 1 | 0.012103 |
| GO:0051019 | mitogen-activated protein kinase binding | Molecular Function | 3 | 0 | 0.012114 |
| GO:0071889 | 14-3-3 protein binding | Molecular Function | 2 | 1 | 0.012114 |
| GO:0005874 | microtubule | Cellular Component | 8 | 5 | 0.012147 |
| GO:0008083 | growth factor activity | Molecular Function | 7 | 0 | 0.012207 |
| GO:0032496 | response to lipopolysaccharide | Biological Process | 9 | 2 | 0.012314 |
| GO:0001836 | release of cytochrome c from mitochondria | Biological Process | 4 | 0 | 0.012401 |
| GO:0002763 | positive regulation of myeloid leukocyte differentiation | Biological Process | 4 | 0 | 0.012401 |
| GO:0046483 | heterocycle metabolic process | Biological Process | 91 | 21 | 0.012451 |
| GO:0055023 | positive regulation of cardiac muscle tissue growth | Biological Process | 2 | 1 | 0.01248 |
| GO:0006112 | energy reserve metabolic process | Biological Process | 3 | 2 | 0.012652 |
| GO:0006919 | activation of cysteine-type endopeptidase activity involved in apoptotic process | Biological Process | 5 | 0 | 0.012652 |
| GO:0060284 | regulation of cell development | Biological Process | 13 | 11 | 0.012814 |
| GO:0003205 | cardiac chamber development | Biological Process | 4 | 3 | 0.012901 |
| GO:0032874 | positive regulation of stress-activated MAPK cascade | Biological Process | 5 | 2 | 0.012901 |
| GO:0009743 | response to carbohydrate | Biological Process | 8 | 1 | 0.012971 |
| GO:0030968 | endoplasmic reticulum unfolded protein response | Biological Process | 6 | 0 | 0.013152 |
| GO:0045598 | regulation of fat cell differentiation | Biological Process | 3 | 3 | 0.013152 |
| GO:0030291 | protein serine/threonine kinase inhibitor activity | Molecular Function | 2 | 1 | 0.01318 |
| GO:0003333 | amino acid transmembrane transport | Biological Process | 5 | 0 | 0.013218 |
| GO:0032436 | positive regulation of proteasomal ubiquitin-dependent protein catabolic process | Biological Process | 4 | 1 | 0.013218 |
| GO:0004861 | cyclin-dependent protein serine/threonine kinase inhibitor activity | Molecular Function | 1 | 1 | 0.013481 |
| GO:0035004 | phosphatidylinositol 3-kinase activity | Molecular Function | 0 | 2 | 0.013481 |
| GO:0006536 | glutamate metabolic process | Biological Process | 3 | 0 | 0.013577 |
| GO:0032633 | interleukin-4 production | Biological Process | 3 | 0 | 0.013577 |
| GO:0032673 | regulation of interleukin-4 production | Biological Process | 3 | 0 | 0.013577 |
| GO:0048536 | spleen development | Biological Process | 2 | 1 | 0.013577 |
| GO:0086005 | ventricular cardiac muscle cell action potential | Biological Process | 1 | 2 | 0.013577 |
| GO:1902235 | regulation of endoplasmic reticulum stress-induced intrinsic apoptotic signaling pathway | Biological Process | 3 | 0 | 0.013577 |
| GO:0071383 | cellular response to steroid hormone stimulus | Biological Process | 6 | 3 | 0.013616 |
| GO:0071375 | cellular response to peptide hormone stimulus | Biological Process | 10 | 1 | 0.013648 |
| GO:0070304 | positive regulation of stress-activated protein kinase signaling cascade | Biological Process | 5 | 2 | 0.013715 |
| GO:0000185 | activation of MAPKKK activity | Biological Process | 2 | 0 | 0.013769 |
| GO:0006995 | cellular response to nitrogen starvation | Biological Process | 2 | 0 | 0.013769 |
| GO:0010739 | positive regulation of protein kinase A signaling | Biological Process | 2 | 0 | 0.013769 |
| GO:0015803 | branched-chain amino acid transport | Biological Process | 2 | 0 | 0.013769 |
| GO:0032966 | negative regulation of collagen biosynthetic process | Biological Process | 2 | 0 | 0.013769 |
| GO:0043562 | cellular response to nitrogen levels | Biological Process | 2 | 0 | 0.013769 |
| GO:0048096 | chromatin-mediated maintenance of transcription | Biological Process | 2 | 0 | 0.013769 |
| GO:0060670 | branching involved in labyrinthine layer morphogenesis | Biological Process | 1 | 1 | 0.013769 |
| GO:1990440 | positive regulation of transcription from RNA polymerase II promoter in response to endoplasmic reticulum stress | Biological Process | 2 | 0 | 0.013769 |
| GO:0098659 | inorganic cation import across plasma membrane | Biological Process | 2 | 3 | 0.0138 |
| GO:0099587 | inorganic ion import across plasma membrane | Biological Process | 2 | 3 | 0.0138 |
| GO:0051145 | smooth muscle cell differentiation | Biological Process | 3 | 1 | 0.013889 |
| GO:1902686 | mitochondrial outer membrane permeabilization involved in programmed cell death | Biological Process | 4 | 0 | 0.013889 |
| GO:0033273 | response to vitamin | Biological Process | 4 | 1 | 0.0144 |
| GO:0048812 | neuron projection morphogenesis | Biological Process | 8 | 10 | 0.014403 |
| GO:0002685 | regulation of leukocyte migration | Biological Process | 6 | 2 | 0.01453 |
| GO:1901215 | negative regulation of neuron death | Biological Process | 8 | 0 | 0.01453 |
| GO:0043509 | activin A complex | Cellular Component | 1 | 0 | 0.01464 |
| GO:0044195 | nucleoplasmic reticulum | Cellular Component | 1 | 0 | 0.01464 |
| GO:0044225 | apical pole of neuron | Cellular Component | 1 | 0 | 0.01464 |
| GO:0048180 | activin complex | Cellular Component | 1 | 0 | 0.01464 |
| GO:0061828 | apical tubulobulbar complex | Cellular Component | 0 | 1 | 0.01464 |
| GO:0061829 | basal tubulobulbar complex | Cellular Component | 0 | 1 | 0.01464 |
| GO:0097140 | BIM-BCL-xl complex | Cellular Component | 1 | 0 | 0.01464 |
| GO:0097141 | BIM-BCL-2 complex | Cellular Component | 1 | 0 | 0.01464 |
| GO:1990332 | Ire1 complex | Cellular Component | 1 | 0 | 0.01464 |
| GO:0032102 | negative regulation of response to external stimulus | Biological Process | 13 | 2 | 0.014643 |
| GO:0035924 | cellular response to vascular endothelial growth factor stimulus | Biological Process | 4 | 0 | 0.014674 |
| GO:0043388 | positive regulation of DNA binding | Biological Process | 4 | 0 | 0.014674 |
| GO:0060135 | maternal process involved in female pregnancy | Biological Process | 4 | 0 | 0.014674 |
| GO:0043537 | negative regulation of blood vessel endothelial cell migration | Biological Process | 3 | 0 | 0.014726 |
| GO:0048821 | erythrocyte development | Biological Process | 3 | 0 | 0.014726 |
| GO:0070306 | lens fiber cell differentiation | Biological Process | 2 | 1 | 0.014726 |
| GO:0006006 | glucose metabolic process | Biological Process | 6 | 2 | 0.014916 |
| GO:0019318 | hexose metabolic process | Biological Process | 6 | 3 | 0.014977 |
| GO:0005294 | neutral L-amino acid secondary active transmembrane transporter activity | Molecular Function | 1 | 0 | 0.015048 |
| GO:0008482 | sulfite oxidase activity | Molecular Function | 0 | 1 | 0.015048 |
| GO:0008859 | exoribonuclease II activity | Molecular Function | 1 | 0 | 0.015048 |
| GO:0015327 | cystine:glutamate antiporter activity | Molecular Function | 1 | 0 | 0.015048 |
| GO:0015328 | cystine secondary active transmembrane transporter activity | Molecular Function | 1 | 0 | 0.015048 |
| GO:0019912 | cyclin-dependent protein kinase activating kinase activity | Molecular Function | 1 | 0 | 0.015048 |
| GO:0030377 | urokinase plasminogen activator receptor activity | Molecular Function | 1 | 0 | 0.015048 |
| GO:0031700 | adrenomedullin receptor binding | Molecular Function | 1 | 0 | 0.015048 |
| GO:0031798 | type 1 metabotropic glutamate receptor binding | Molecular Function | 0 | 1 | 0.015048 |
| GO:0032440 | 2-alkenal reductase [NAD(P)] activity | Molecular Function | 1 | 0 | 0.015048 |
| GO:0032841 | calcitonin binding | Molecular Function | 1 | 0 | 0.015048 |
| GO:0033746 | histone demethylase activity (H3-R2 specific) | Molecular Function | 1 | 0 | 0.015048 |
| GO:0033749 | histone demethylase activity (H4-R3 specific) | Molecular Function | 1 | 0 | 0.015048 |
| GO:0035226 | glutamate-cysteine ligase catalytic subunit binding | Molecular Function | 1 | 0 | 0.015048 |
| GO:0035798 | 2-alkenal reductase (NADP+) activity | Molecular Function | 1 | 0 | 0.015048 |
| GO:0036185 | 13-lipoxin reductase activity | Molecular Function | 1 | 0 | 0.015048 |
| GO:0036463 | TRAIL receptor activity | Molecular Function | 1 | 0 | 0.015048 |
| GO:0044374 | sequence-specific DNA binding, bending | Molecular Function | 1 | 0 | 0.015048 |
| GO:0044377 | RNA polymerase II proximal promoter sequence-specific DNA binding, bending | Molecular Function | 1 | 0 | 0.015048 |
| GO:0047464 | heparosan-N-sulfate-glucuronate 5-epimerase activity | Molecular Function | 0 | 1 | 0.015048 |
| GO:0047933 | glucose-1,6-bisphosphate synthase activity | Molecular Function | 0 | 1 | 0.015048 |
| GO:0070119 | ciliary neurotrophic factor binding | Molecular Function | 1 | 0 | 0.015048 |
| GO:0070551 | endoribonuclease activity, cleaving siRNA-paired mRNA | Molecular Function | 1 | 0 | 0.015048 |
| GO:0097257 | leukotriene B4 12-hydroxy dehydrogenase activity | Molecular Function | 1 | 0 | 0.015048 |
| GO:0102200 | N-acetylphosphatidylethanolamine-hydrolysing phospholipas activity | Molecular Function | 0 | 1 | 0.015048 |
| GO:1901680 | sulfur-containing amino acid secondary active transmembrane transporter activity | Molecular Function | 1 | 0 | 0.015048 |
| GO:0003002 | regionalization | Biological Process | 7 | 4 | 0.015089 |
| GO:0001100 | negative regulation of exit from mitosis | Biological Process | 1 | 0 | 0.015217 |
| GO:0001808 | negative regulation of type IV hypersensitivity | Biological Process | 1 | 0 | 0.015217 |
| GO:0002575 | basophil chemotaxis | Biological Process | 1 | 0 | 0.015217 |
| GO:0009444 | pyruvate oxidation | Biological Process | 1 | 0 | 0.015217 |
| GO:0010383 | cell wall polysaccharide metabolic process | Biological Process | 1 | 0 | 0.015217 |
| GO:0010849 | regulation of proton-transporting ATPase activity, rotational mechanism | Biological Process | 1 | 0 | 0.015217 |
| GO:0018395 | peptidyl-lysine hydroxylation to 5-hydroxy-L-lysine | Biological Process | 1 | 0 | 0.015217 |
| GO:0021594 | rhombomere formation | Biological Process | 1 | 0 | 0.015217 |
| GO:0021595 | rhombomere structural organization | Biological Process | 1 | 0 | 0.015217 |
| GO:0021659 | rhombomere 3 structural organization | Biological Process | 1 | 0 | 0.015217 |
| GO:0021660 | rhombomere 3 formation | Biological Process | 1 | 0 | 0.015217 |
| GO:0021664 | rhombomere 5 morphogenesis | Biological Process | 1 | 0 | 0.015217 |
| GO:0021665 | rhombomere 5 structural organization | Biological Process | 1 | 0 | 0.015217 |
| GO:0021666 | rhombomere 5 formation | Biological Process | 1 | 0 | 0.015217 |
| GO:0034395 | regulation of transcription from RNA polymerase II promoter in response to iron | Biological Process | 1 | 0 | 0.015217 |
| GO:0035227 | regulation of glutamate-cysteine ligase activity | Biological Process | 1 | 0 | 0.015217 |
| GO:0035229 | positive regulation of glutamate-cysteine ligase activity | Biological Process | 1 | 0 | 0.015217 |
| GO:0035602 | fibroblast growth factor receptor signaling pathway involved in negative regulation of apoptotic process in bone marrow | Biological Process | 0 | 1 | 0.015217 |
| GO:0035603 | fibroblast growth factor receptor signaling pathway involved in hemopoiesis | Biological Process | 0 | 1 | 0.015217 |
| GO:0035604 | fibroblast growth factor receptor signaling pathway involved in positive regulation of cell proliferation in bone marrow | Biological Process | 0 | 1 | 0.015217 |
| GO:0035607 | fibroblast growth factor receptor signaling pathway involved in orbitofrontal cortex development | Biological Process | 0 | 1 | 0.015217 |
| GO:0035853 | chromosome passenger complex localization to spindle midzone | Biological Process | 1 | 0 | 0.015217 |
| GO:0035921 | desmosome disassembly | Biological Process | 0 | 1 | 0.015217 |
| GO:0036290 | protein trans-autophosphorylation | Biological Process | 1 | 0 | 0.015217 |
| GO:0036497 | eIF2alpha dephosphorylation in response to endoplasmic reticulum stress | Biological Process | 1 | 0 | 0.015217 |
| GO:0038097 | positive regulation of mast cell activation by Fc-epsilon receptor signaling pathway | Biological Process | 1 | 0 | 0.015217 |
| GO:0045975 | positive regulation of translation, ncRNA-mediated | Biological Process | 1 | 0 | 0.015217 |
| GO:0046293 | formaldehyde biosynthetic process | Biological Process | 1 | 0 | 0.015217 |
| GO:0046722 | lactic acid secretion | Biological Process | 1 | 0 | 0.015217 |
| GO:0051543 | regulation of elastin biosynthetic process | Biological Process | 1 | 0 | 0.015217 |
| GO:0051545 | negative regulation of elastin biosynthetic process | Biological Process | 1 | 0 | 0.015217 |
| GO:0051913 | regulation of synaptic plasticity by chemical substance | Biological Process | 1 | 0 | 0.015217 |
| GO:0051914 | positive regulation of synaptic plasticity by chemical substance | Biological Process | 1 | 0 | 0.015217 |
| GO:0051915 | induction of synaptic plasticity by chemical substance | Biological Process | 1 | 0 | 0.015217 |
| GO:0060034 | notochord cell differentiation | Biological Process | 1 | 0 | 0.015217 |
| GO:0060035 | notochord cell development | Biological Process | 1 | 0 | 0.015217 |
| GO:0060598 | dichotomous subdivision of terminal units involved in mammary gland duct morphogenesis | Biological Process | 1 | 0 | 0.015217 |
| GO:0061301 | cerebellum vasculature morphogenesis | Biological Process | 0 | 1 | 0.015217 |
| GO:0061956 | penetration of cumulus oophorus | Biological Process | 1 | 0 | 0.015217 |
| GO:0070054 | mRNA splicing, via endonucleolytic cleavage and ligation | Biological Process | 1 | 0 | 0.015217 |
| GO:0070077 | histone arginine demethylation | Biological Process | 1 | 0 | 0.015217 |
| GO:0070078 | histone H3-R2 demethylation | Biological Process | 1 | 0 | 0.015217 |
| GO:0070079 | histone H4-R3 demethylation | Biological Process | 1 | 0 | 0.015217 |
| GO:0070962 | positive regulation of neutrophil mediated killing of bacterium | Biological Process | 1 | 0 | 0.015217 |
| GO:0070963 | positive regulation of neutrophil mediated killing of gram-negative bacterium | Biological Process | 1 | 0 | 0.015217 |
| GO:0070981 | L-asparagine biosynthetic process | Biological Process | 1 | 0 | 0.015217 |
| GO:0071301 | cellular response to vitamin B1 | Biological Process | 1 | 0 | 0.015217 |
| GO:0071606 | chemokine (C-C motif) ligand 4 production | Biological Process | 1 | 0 | 0.015217 |
| GO:0071643 | regulation of chemokine (C-C motif) ligand 4 production | Biological Process | 1 | 0 | 0.015217 |
| GO:0071644 | negative regulation of chemokine (C-C motif) ligand 4 production | Biological Process | 1 | 0 | 0.015217 |
| GO:0071678 | olfactory bulb axon guidance | Biological Process | 0 | 1 | 0.015217 |
| GO:0071852 | fungal-type cell wall organization or biogenesis | Biological Process | 1 | 0 | 0.015217 |
| GO:0071877 | regulation of adenylate cyclase-inhibiting adrenergic receptor signaling pathway | Biological Process | 1 | 0 | 0.015217 |
| GO:0071966 | fungal-type cell wall polysaccharide metabolic process | Biological Process | 1 | 0 | 0.015217 |
| GO:0072303 | positive regulation of glomerular metanephric mesangial cell proliferation | Biological Process | 1 | 0 | 0.015217 |
| GO:0080170 | hydrogen peroxide transmembrane transport | Biological Process | 0 | 1 | 0.015217 |
| GO:0086096 | adenylate cyclase-inhibiting adrenergic receptor signaling pathway involved in heart process | Biological Process | 1 | 0 | 0.015217 |
| GO:0090095 | regulation of metanephric cap mesenchymal cell proliferation | Biological Process | 1 | 0 | 0.015217 |
| GO:0090096 | positive regulation of metanephric cap mesenchymal cell proliferation | Biological Process | 1 | 0 | 0.015217 |
| GO:0090625 | mRNA cleavage involved in gene silencing by siRNA | Biological Process | 1 | 0 | 0.015217 |
| GO:0110077 | vesicle-mediated intercellular transport | Biological Process | 1 | 0 | 0.015217 |
| GO:0140193 | regulation of adenylate cyclase-inhibiting adrenergic receptor signaling pathway involved in heart process | Biological Process | 1 | 0 | 0.015217 |
| GO:0140194 | negative regulation of adenylate cyclase-inhibiting adrenergic receptor signaling pathway involved in heart process | Biological Process | 1 | 0 | 0.015217 |
| GO:1900036 | positive regulation of cellular response to heat | Biological Process | 1 | 0 | 0.015217 |
| GO:1901494 | regulation of cysteine metabolic process | Biological Process | 1 | 0 | 0.015217 |
| GO:1901676 | positive regulation of histone H3-K27 acetylation | Biological Process | 1 | 0 | 0.015217 |
| GO:1901834 | regulation of deadenylation-independent decapping of nuclear-transcribed mRNA | Biological Process | 1 | 0 | 0.015217 |
| GO:1901835 | positive regulation of deadenylation-independent decapping of nuclear-transcribed mRNA | Biological Process | 1 | 0 | 0.015217 |
| GO:1902010 | negative regulation of translation in response to endoplasmic reticulum stress | Biological Process | 1 | 0 | 0.015217 |
| GO:1902038 | positive regulation of hematopoietic stem cell differentiation | Biological Process | 1 | 0 | 0.015217 |
| GO:1903570 | regulation of protein kinase D signaling | Biological Process | 1 | 0 | 0.015217 |
| GO:1903572 | positive regulation of protein kinase D signaling | Biological Process | 1 | 0 | 0.015217 |
| GO:1903916 | regulation of endoplasmic reticulum stress-induced eIF2 alpha dephosphorylation | Biological Process | 1 | 0 | 0.015217 |
| GO:1903917 | positive regulation of endoplasmic reticulum stress-induced eIF2 alpha dephosphorylation | Biological Process | 1 | 0 | 0.015217 |
| GO:1904246 | negative regulation of polynucleotide adenylyltransferase activity | Biological Process | 1 | 0 | 0.015217 |
| GO:1904404 | response to formaldehyde | Biological Process | 1 | 0 | 0.015217 |
| GO:1904619 | response to dimethyl sulfoxide | Biological Process | 1 | 0 | 0.015217 |
| GO:1904620 | cellular response to dimethyl sulfoxide | Biological Process | 1 | 0 | 0.015217 |
| GO:1904897 | regulation of hepatic stellate cell proliferation | Biological Process | 1 | 0 | 0.015217 |
| GO:1904899 | positive regulation of hepatic stellate cell proliferation | Biological Process | 1 | 0 | 0.015217 |
| GO:1905062 | positive regulation of cardioblast proliferation | Biological Process | 1 | 0 | 0.015217 |
| GO:1990579 | peptidyl-serine trans-autophosphorylation | Biological Process | 1 | 0 | 0.015217 |
| GO:1990840 | response to lectin | Biological Process | 1 | 0 | 0.015217 |
| GO:1990858 | cellular response to lectin | Biological Process | 1 | 0 | 0.015217 |
| GO:1990922 | hepatic stellate cell proliferation | Biological Process | 1 | 0 | 0.015217 |
| GO:2000661 | positive regulation of interleukin-1-mediated signaling pathway | Biological Process | 0 | 1 | 0.015217 |
| GO:2000723 | negative regulation of cardiac vascular smooth muscle cell differentiation | Biological Process | 1 | 0 | 0.015217 |
| GO:0030182 | neuron differentiation | Biological Process | 17 | 15 | 0.015223 |
| GO:2000027 | regulation of animal organ morphogenesis | Biological Process | 5 | 4 | 0.015332 |
| GO:0007409 | axonogenesis | Biological Process | 7 | 7 | 0.015336 |
| GO:0035794 | positive regulation of mitochondrial membrane permeability | Biological Process | 4 | 0 | 0.015487 |
| GO:0045576 | mast cell activation | Biological Process | 3 | 1 | 0.015487 |
| GO:0005996 | monosaccharide metabolic process | Biological Process | 7 | 3 | 0.015558 |
| GO:1901184 | regulation of ERBB signaling pathway | Biological Process | 4 | 1 | 0.015651 |
| GO:0097305 | response to alcohol | Biological Process | 8 | 1 | 0.015694 |
| GO:0032620 | interleukin-17 production | Biological Process | 3 | 0 | 0.015929 |
| GO:0032660 | regulation of interleukin-17 production | Biological Process | 3 | 0 | 0.015929 |
| GO:0033280 | response to vitamin D | Biological Process | 2 | 1 | 0.015929 |
| GO:1904707 | positive regulation of vascular smooth muscle cell proliferation | Biological Process | 3 | 0 | 0.015929 |
| GO:0045785 | positive regulation of cell adhesion | Biological Process | 11 | 2 | 0.015937 |
| GO:0007155 | cell adhesion | Biological Process | 21 | 12 | 0.015943 |
| GO:1901988 | negative regulation of cell cycle phase transition | Biological Process | 7 | 2 | 0.016062 |
| GO:0006983 | ER overload response | Biological Process | 2 | 0 | 0.016111 |
| GO:0010713 | negative regulation of collagen metabolic process | Biological Process | 2 | 0 | 0.016111 |
| GO:0010839 | negative regulation of keratinocyte proliferation | Biological Process | 0 | 2 | 0.016111 |
| GO:0015801 | aromatic amino acid transport | Biological Process | 2 | 0 | 0.016111 |
| GO:0036092 | phosphatidylinositol-3-phosphate biosynthetic process | Biological Process | 0 | 2 | 0.016111 |
| GO:0045741 | positive regulation of epidermal growth factor-activated receptor activity | Biological Process | 2 | 0 | 0.016111 |
| GO:0051709 | regulation of killing of cells of other organism | Biological Process | 2 | 0 | 0.016111 |
| GO:0060100 | positive regulation of phagocytosis, engulfment | Biological Process | 1 | 1 | 0.016111 |
| GO:0060213 | positive regulation of nuclear-transcribed mRNA poly(A) tail shortening | Biological Process | 2 | 0 | 0.016111 |
| GO:0060347 | heart trabecula formation | Biological Process | 2 | 0 | 0.016111 |
| GO:0060707 | trophoblast giant cell differentiation | Biological Process | 2 | 0 | 0.016111 |
| GO:0090192 | regulation of glomerulus development | Biological Process | 2 | 0 | 0.016111 |
| GO:1902043 | positive regulation of extrinsic apoptotic signaling pathway via death domain receptors | Biological Process | 2 | 0 | 0.016111 |
| GO:1905155 | positive regulation of membrane invagination | Biological Process | 1 | 1 | 0.016111 |
| GO:0098609 | cell-cell adhesion | Biological Process | 15 | 7 | 0.016175 |
| GO:0006749 | glutathione metabolic process | Biological Process | 4 | 0 | 0.016327 |
| GO:0045844 | positive regulation of striated muscle tissue development | Biological Process | 3 | 1 | 0.016327 |
| GO:0048636 | positive regulation of muscle organ development | Biological Process | 3 | 1 | 0.016327 |
| GO:0048568 | embryonic organ development | Biological Process | 8 | 5 | 0.016491 |
| GO:0015175 | neutral amino acid transmembrane transporter activity | Molecular Function | 3 | 0 | 0.016689 |
| GO:0045766 | positive regulation of angiogenesis | Biological Process | 5 | 2 | 0.016853 |
| GO:0030178 | negative regulation of Wnt signaling pathway | Biological Process | 3 | 5 | 0.016961 |
| GO:0042802 | identical protein binding | Molecular Function | 32 | 11 | 0.016986 |
| GO:0003203 | endocardial cushion morphogenesis | Biological Process | 2 | 1 | 0.017186 |
| GO:0030224 | monocyte differentiation | Biological Process | 2 | 1 | 0.017186 |
| GO:0051084 | 'de novo' posttranslational protein folding | Biological Process | 3 | 0 | 0.017186 |
| GO:0060421 | positive regulation of heart growth | Biological Process | 2 | 1 | 0.017186 |
| GO:0099622 | cardiac muscle cell membrane repolarization | Biological Process | 1 | 2 | 0.017186 |
| GO:1903131 | mononuclear cell differentiation | Biological Process | 2 | 1 | 0.017186 |
| GO:0014855 | striated muscle cell proliferation | Biological Process | 3 | 1 | 0.017195 |
| GO:0045600 | positive regulation of fat cell differentiation | Biological Process | 2 | 2 | 0.017195 |
| GO:1901863 | positive regulation of muscle tissue development | Biological Process | 3 | 1 | 0.017195 |
| GO:0046330 | positive regulation of JNK cascade | Biological Process | 4 | 2 | 0.017306 |
| GO:0045787 | positive regulation of cell cycle | Biological Process | 11 | 1 | 0.017306 |
| GO:0043112 | receptor metabolic process | Biological Process | 5 | 2 | 0.017339 |
| GO:1902806 | regulation of cell cycle G1/S phase transition | Biological Process | 5 | 2 | 0.017339 |
| GO:0048738 | cardiac muscle tissue development | Biological Process | 6 | 2 | 0.017393 |
| GO:0120039 | plasma membrane bounded cell projection morphogenesis | Biological Process | 8 | 10 | 0.017396 |
| GO:0002237 | response to molecule of bacterial origin | Biological Process | 9 | 2 | 0.01763 |
| GO:0007631 | feeding behavior | Biological Process | 4 | 1 | 0.01766 |
| GO:0034766 | negative regulation of ion transmembrane transport | Biological Process | 5 | 0 | 0.01766 |
| GO:0051960 | regulation of nervous system development | Biological Process | 11 | 12 | 0.017866 |
| GO:0046683 | response to organophosphorus | Biological Process | 5 | 1 | 0.017882 |
| GO:0035259 | glucocorticoid receptor binding | Molecular Function | 2 | 0 | 0.018223 |
| GO:0048858 | cell projection morphogenesis | Biological Process | 8 | 10 | 0.018334 |
| GO:0050730 | regulation of peptidyl-tyrosine phosphorylation | Biological Process | 7 | 2 | 0.018403 |
| GO:1902911 | protein kinase complex | Cellular Component | 4 | 1 | 0.018443 |
| GO:0017148 | negative regulation of translation | Biological Process | 6 | 0 | 0.018471 |
| GO:0072175 | epithelial tube formation | Biological Process | 3 | 3 | 0.018471 |
| GO:0043516 | regulation of DNA damage response, signal transduction by p53 class mediator | Biological Process | 2 | 1 | 0.018497 |
| GO:0060428 | lung epithelium development | Biological Process | 2 | 1 | 0.018497 |
| GO:0007567 | parturition | Biological Process | 1 | 1 | 0.01861 |
| GO:0010561 | negative regulation of glycoprotein biosynthetic process | Biological Process | 2 | 0 | 0.01861 |
| GO:0045986 | negative regulation of smooth muscle contraction | Biological Process | 1 | 1 | 0.01861 |
| GO:0060712 | spongiotrophoblast layer development | Biological Process | 2 | 0 | 0.01861 |
| GO:0060992 | response to fungicide | Biological Process | 2 | 0 | 0.01861 |
| GO:0061469 | regulation of type B pancreatic cell proliferation | Biological Process | 2 | 0 | 0.01861 |
| GO:0097154 | GABAergic neuron differentiation | Biological Process | 1 | 1 | 0.01861 |
| GO:1900103 | positive regulation of endoplasmic reticulum unfolded protein response | Biological Process | 2 | 0 | 0.01861 |
| GO:1901163 | regulation of trophoblast cell migration | Biological Process | 2 | 0 | 0.01861 |
| GO:1903025 | regulation of RNA polymerase II regulatory region sequence-specific DNA binding | Biological Process | 2 | 0 | 0.01861 |
| GO:0045321 | leukocyte activation | Biological Process | 24 | 6 | 0.018764 |
| GO:0030217 | T cell differentiation | Biological Process | 7 | 2 | 0.018816 |
| GO:0060415 | muscle tissue morphogenesis | Biological Process | 2 | 2 | 0.019016 |
| GO:1905710 | positive regulation of membrane permeability | Biological Process | 4 | 0 | 0.019016 |
| GO:0035051 | cardiocyte differentiation | Biological Process | 4 | 2 | 0.019072 |
| GO:0000164 | protein phosphatase type 1 complex | Cellular Component | 1 | 1 | 0.019776 |
| GO:1905037 | autophagosome organization | Biological Process | 3 | 2 | 0.019833 |
| GO:0007223 | Wnt signaling pathway, calcium modulating pathway | Biological Process | 1 | 2 | 0.019861 |
| GO:0042311 | vasodilation | Biological Process | 3 | 0 | 0.019861 |
| GO:0045742 | positive regulation of epidermal growth factor receptor signaling pathway | Biological Process | 3 | 0 | 0.019861 |
| GO:0060412 | ventricular septum morphogenesis | Biological Process | 1 | 2 | 0.019861 |
| GO:0060411 | cardiac septum morphogenesis | Biological Process | 2 | 2 | 0.01997 |
| GO:0099175 | regulation of postsynapse organization | Biological Process | 2 | 3 | 0.020595 |
| GO:0070851 | growth factor receptor binding | Molecular Function | 5 | 1 | 0.020606 |
| GO:0042813 | Wnt-activated receptor activity | Molecular Function | 0 | 2 | 0.02082 |
| GO:0050700 | CARD domain binding | Molecular Function | 2 | 0 | 0.02082 |
| GO:0010633 | negative regulation of epithelial cell migration | Biological Process | 4 | 0 | 0.020953 |
| GO:0016239 | positive regulation of macroautophagy | Biological Process | 4 | 0 | 0.020953 |
| GO:0032722 | positive regulation of chemokine production | Biological Process | 4 | 0 | 0.020953 |
| GO:2000573 | positive regulation of DNA biosynthetic process | Biological Process | 4 | 0 | 0.020953 |
| GO:0019904 | protein domain specific binding | Molecular Function | 13 | 5 | 0.020956 |
| GO:0045930 | negative regulation of mitotic cell cycle | Biological Process | 8 | 2 | 0.021145 |
| GO:0002070 | epithelial cell maturation | Biological Process | 2 | 0 | 0.02126 |
| GO:0032700 | negative regulation of interleukin-17 production | Biological Process | 2 | 0 | 0.02126 |
| GO:0042976 | activation of Janus kinase activity | Biological Process | 2 | 0 | 0.02126 |
| GO:0043518 | negative regulation of DNA damage response, signal transduction by p53 class mediator | Biological Process | 1 | 1 | 0.02126 |
| GO:0046321 | positive regulation of fatty acid oxidation | Biological Process | 2 | 0 | 0.02126 |
| GO:0050930 | induction of positive chemotaxis | Biological Process | 2 | 0 | 0.02126 |
| GO:0060099 | regulation of phagocytosis, engulfment | Biological Process | 1 | 1 | 0.02126 |
| GO:0060211 | regulation of nuclear-transcribed mRNA poly(A) tail shortening | Biological Process | 2 | 0 | 0.02126 |
| GO:0061450 | trophoblast cell migration | Biological Process | 2 | 0 | 0.02126 |
| GO:0072109 | glomerular mesangium development | Biological Process | 2 | 0 | 0.02126 |
| GO:0004722 | protein serine/threonine phosphatase activity | Molecular Function | 4 | 1 | 0.021262 |
| GO:0030669 | clathrin-coated endocytic vesicle membrane | Cellular Component | 2 | 2 | 0.021263 |
| GO:0043029 | T cell homeostasis | Biological Process | 3 | 0 | 0.02128 |
| GO:0006855 | drug transmembrane transport | Biological Process | 4 | 1 | 0.021375 |
| GO:0032963 | collagen metabolic process | Biological Process | 4 | 1 | 0.021375 |
| GO:0004713 | protein tyrosine kinase activity | Molecular Function | 3 | 3 | 0.021902 |
| GO:0046834 | lipid phosphorylation | Biological Process | 2 | 2 | 0.021965 |
| GO:0061180 | mammary gland epithelium development | Biological Process | 3 | 1 | 0.021965 |
| GO:0003279 | cardiac septum development | Biological Process | 2 | 3 | 0.022175 |
| GO:0044773 | mitotic DNA damage checkpoint | Biological Process | 5 | 0 | 0.022175 |
| GO:0032355 | response to estradiol | Biological Process | 6 | 0 | 0.022284 |
| GO:0098657 | import into cell | Biological Process | 16 | 8 | 0.022352 |
| GO:0019199 | transmembrane receptor protein kinase activity | Molecular Function | 3 | 3 | 0.022569 |
| GO:0001974 | blood vessel remodeling | Biological Process | 3 | 0 | 0.022752 |
| GO:0006458 | 'de novo' protein folding | Biological Process | 3 | 0 | 0.022752 |
| GO:0055025 | positive regulation of cardiac muscle tissue development | Biological Process | 2 | 1 | 0.022752 |
| GO:0060043 | regulation of cardiac muscle cell proliferation | Biological Process | 2 | 1 | 0.022752 |
| GO:1901186 | positive regulation of ERBB signaling pathway | Biological Process | 3 | 0 | 0.022752 |
| GO:0032990 | cell part morphogenesis | Biological Process | 8 | 10 | 0.022767 |
| GO:0060047 | heart contraction | Biological Process | 6 | 3 | 0.022842 |
| GO:0043500 | muscle adaptation | Biological Process | 5 | 0 | 0.022993 |
| GO:0048640 | negative regulation of developmental growth | Biological Process | 3 | 2 | 0.022993 |
| GO:0071479 | cellular response to ionizing radiation | Biological Process | 4 | 0 | 0.023007 |
| GO:0090398 | cellular senescence | Biological Process | 2 | 2 | 0.023007 |
| GO:0046935 | 1-phosphatidylinositol-3-kinase regulator activity | Molecular Function | 1 | 1 | 0.023562 |
| GO:0052742 | phosphatidylinositol kinase activity | Molecular Function | 0 | 2 | 0.023562 |
| GO:1990404 | protein ADP-ribosylase activity | Molecular Function | 1 | 1 | 0.023562 |
| GO:0048762 | mesenchymal cell differentiation | Biological Process | 3 | 5 | 0.02375 |
| GO:0048667 | cell morphogenesis involved in neuron differentiation | Biological Process | 8 | 8 | 0.023845 |
| GO:0003198 | epithelial to mesenchymal transition involved in endocardial cushion formation | Biological Process | 1 | 1 | 0.024056 |
| GO:0032352 | positive regulation of hormone metabolic process | Biological Process | 2 | 0 | 0.024056 |
| GO:0045475 | locomotor rhythm | Biological Process | 2 | 0 | 0.024056 |
| GO:0045651 | positive regulation of macrophage differentiation | Biological Process | 2 | 0 | 0.024056 |
| GO:0055012 | ventricular cardiac muscle cell differentiation | Biological Process | 2 | 0 | 0.024056 |
| GO:0060977 | coronary vasculature morphogenesis | Biological Process | 2 | 0 | 0.024056 |
| GO:0090336 | positive regulation of brown fat cell differentiation | Biological Process | 1 | 1 | 0.024056 |
| GO:1903960 | negative regulation of anion transmembrane transport | Biological Process | 2 | 0 | 0.024056 |
| GO:1905153 | regulation of membrane invagination | Biological Process | 1 | 1 | 0.024056 |
| GO:0032729 | positive regulation of interferon-gamma production | Biological Process | 3 | 1 | 0.024078 |
| GO:0038034 | signal transduction in absence of ligand | Biological Process | 3 | 1 | 0.024078 |
| GO:0086001 | cardiac muscle cell action potential | Biological Process | 1 | 3 | 0.024078 |
| GO:0097192 | extrinsic apoptotic signaling pathway in absence of ligand | Biological Process | 3 | 1 | 0.024078 |
| GO:2000243 | positive regulation of reproductive process | Biological Process | 4 | 0 | 0.024078 |
| GO:0010720 | positive regulation of cell development | Biological Process | 9 | 6 | 0.024201 |
| GO:0032350 | regulation of hormone metabolic process | Biological Process | 3 | 0 | 0.024279 |
| GO:0045823 | positive regulation of heart contraction | Biological Process | 3 | 0 | 0.024279 |
| GO:0019887 | protein kinase regulator activity | Molecular Function | 6 | 1 | 0.024554 |
| GO:0055007 | cardiac muscle cell differentiation | Biological Process | 4 | 1 | 0.024688 |
| GO:0060322 | head development | Biological Process | 12 | 8 | 0.024849 |
| GO:0002699 | positive regulation of immune effector process | Biological Process | 6 | 2 | 0.024856 |
| GO:0033627 | cell adhesion mediated by integrin | Biological Process | 2 | 2 | 0.025179 |
| GO:0043123 | positive regulation of I-kappaB kinase/NF-kappaB signaling | Biological Process | 6 | 1 | 0.025223 |
| GO:0009611 | response to wounding | Biological Process | 14 | 4 | 0.025423 |
| GO:0044774 | mitotic DNA integrity checkpoint | Biological Process | 5 | 0 | 0.025565 |
| GO:2000060 | positive regulation of ubiquitin-dependent protein catabolic process | Biological Process | 4 | 1 | 0.025565 |
| GO:0009913 | epidermal cell differentiation | Biological Process | 10 | 1 | 0.025806 |
| GO:0010212 | response to ionizing radiation | Biological Process | 6 | 0 | 0.025841 |
| GO:0051146 | striated muscle cell differentiation | Biological Process | 7 | 2 | 0.025848 |
| GO:0000096 | sulfur amino acid metabolic process | Biological Process | 3 | 0 | 0.02586 |
| GO:0030851 | granulocyte differentiation | Biological Process | 2 | 1 | 0.02586 |
| GO:0032689 | negative regulation of interferon-gamma production | Biological Process | 3 | 0 | 0.02586 |
| GO:0033003 | regulation of mast cell activation | Biological Process | 2 | 1 | 0.02586 |
| GO:0045616 | regulation of keratinocyte differentiation | Biological Process | 3 | 0 | 0.02586 |
| GO:1903524 | positive regulation of blood circulation | Biological Process | 4 | 0 | 0.026309 |
| GO:0045664 | regulation of neuron differentiation | Biological Process | 6 | 11 | 0.026507 |
| GO:0030544 | Hsp70 protein binding | Molecular Function | 3 | 0 | 0.026723 |
| GO:0001946 | lymphangiogenesis | Biological Process | 2 | 0 | 0.026994 |
| GO:0010566 | regulation of ketone biosynthetic process | Biological Process | 2 | 0 | 0.026994 |
| GO:0035278 | miRNA mediated inhibition of translation | Biological Process | 2 | 0 | 0.026994 |
| GO:0040033 | negative regulation of translation, ncRNA-mediated | Biological Process | 2 | 0 | 0.026994 |
| GO:0043558 | regulation of translational initiation in response to stress | Biological Process | 2 | 0 | 0.026994 |
| GO:0045974 | regulation of translation, ncRNA-mediated | Biological Process | 2 | 0 | 0.026994 |
| GO:0048521 | negative regulation of behavior | Biological Process | 1 | 1 | 0.026994 |
| GO:0051782 | negative regulation of cell division | Biological Process | 2 | 0 | 0.026994 |
| GO:0055119 | relaxation of cardiac muscle | Biological Process | 1 | 1 | 0.026994 |
| GO:0061158 | 3'-UTR-mediated mRNA destabilization | Biological Process | 2 | 0 | 0.026994 |
| GO:0090083 | regulation of inclusion body assembly | Biological Process | 2 | 0 | 0.026994 |
| GO:1900424 | regulation of defense response to bacterium | Biological Process | 2 | 0 | 0.026994 |
| GO:1903019 | negative regulation of glycoprotein metabolic process | Biological Process | 2 | 0 | 0.026994 |
| GO:2000269 | regulation of fibroblast apoptotic process | Biological Process | 2 | 0 | 0.026994 |
| GO:2001212 | regulation of vasculogenesis | Biological Process | 2 | 0 | 0.026994 |
| GO:2001267 | regulation of cysteine-type endopeptidase activity involved in apoptotic signaling pathway | Biological Process | 2 | 0 | 0.026994 |
| GO:0006417 | regulation of translation | Biological Process | 10 | 1 | 0.027167 |
| GO:0001889 | liver development | Biological Process | 5 | 1 | 0.027364 |
| GO:0014015 | positive regulation of gliogenesis | Biological Process | 4 | 0 | 0.02747 |
| GO:0033692 | cellular polysaccharide biosynthetic process | Biological Process | 1 | 3 | 0.02747 |
| GO:0045454 | cell redox homeostasis | Biological Process | 3 | 0 | 0.027495 |
| GO:1903672 | positive regulation of sprouting angiogenesis | Biological Process | 2 | 1 | 0.027495 |
| GO:0008016 | regulation of heart contraction | Biological Process | 6 | 2 | 0.027777 |
| GO:0032101 | regulation of response to external stimulus | Biological Process | 18 | 6 | 0.027878 |
| GO:1903037 | regulation of leukocyte cell-cell adhesion | Biological Process | 9 | 1 | 0.02807 |
| GO:0051082 | unfolded protein binding | Molecular Function | 5 | 0 | 0.028095 |
| GO:0072009 | nephron epithelium development | Biological Process | 4 | 1 | 0.028315 |
| GO:2000377 | regulation of reactive oxygen species metabolic process | Biological Process | 7 | 0 | 0.028552 |
| GO:0007548 | sex differentiation | Biological Process | 8 | 1 | 0.028558 |
| GO:0022409 | positive regulation of cell-cell adhesion | Biological Process | 8 | 1 | 0.028558 |
| GO:0072593 | reactive oxygen species metabolic process | Biological Process | 9 | 0 | 0.028558 |
| GO:0055024 | regulation of cardiac muscle tissue development | Biological Process | 3 | 1 | 0.028661 |
| GO:0061564 | axon development | Biological Process | 7 | 7 | 0.028941 |
| GO:2000241 | regulation of reproductive process | Biological Process | 6 | 0 | 0.028945 |
| GO:0060560 | developmental growth involved in morphogenesis | Biological Process | 4 | 4 | 0.029009 |
| GO:0002096 | polkadots | Cellular Component | 1 | 0 | 0.029067 |
| GO:0033257 | Bcl3/NF-kappaB2 complex | Cellular Component | 1 | 0 | 0.029067 |
| GO:0034686 | integrin alphav-beta8 complex | Cellular Component | 0 | 1 | 0.029067 |
| GO:0036284 | tubulobulbar complex | Cellular Component | 0 | 1 | 0.029067 |
| GO:0036488 | CHOP-C/EBP complex | Cellular Component | 1 | 0 | 0.029067 |
| GO:0043511 | inhibin complex | Cellular Component | 1 | 0 | 0.029067 |
| GO:0043512 | inhibin A complex | Cellular Component | 1 | 0 | 0.029067 |
| GO:0060187 | cell pole | Cellular Component | 1 | 0 | 0.029067 |
| GO:0070557 | PCNA-p21 complex | Cellular Component | 1 | 0 | 0.029067 |
| GO:0150056 | amylin receptor complex 1 | Cellular Component | 1 | 0 | 0.029067 |
| GO:0150057 | amylin receptor complex 2 | Cellular Component | 1 | 0 | 0.029067 |
| GO:1990597 | AIP1-IRE1 complex | Cellular Component | 1 | 0 | 0.029067 |
| GO:1990617 | CHOP-ATF4 complex | Cellular Component | 1 | 0 | 0.029067 |
| GO:1990630 | IRE1-RACK1-PP2A complex | Cellular Component | 1 | 0 | 0.029067 |
| GO:0002832 | negative regulation of response to biotic stimulus | Biological Process | 3 | 0 | 0.029184 |
| GO:0060711 | labyrinthine layer development | Biological Process | 2 | 1 | 0.029184 |
| GO:0061383 | trabecula morphogenesis | Biological Process | 2 | 1 | 0.029184 |
| GO:0070266 | necroptotic process | Biological Process | 3 | 0 | 0.029184 |
| GO:2000273 | positive regulation of signaling receptor activity | Biological Process | 3 | 0 | 0.029184 |
| GO:0008301 | DNA binding, bending | Molecular Function | 2 | 0 | 0.029456 |
| GO:0047834 | D-threo-aldose 1-dehydrogenase activity | Molecular Function | 2 | 0 | 0.029456 |
| GO:0043588 | skin development | Biological Process | 9 | 3 | 0.029523 |
| GO:1901991 | negative regulation of mitotic cell cycle phase transition | Biological Process | 6 | 2 | 0.02964 |
| GO:0007249 | I-kappaB kinase/NF-kappaB signaling | Biological Process | 7 | 2 | 0.029697 |
| GO:0008277 | regulation of G protein-coupled receptor signaling pathway | Biological Process | 5 | 1 | 0.029758 |
| GO:0014074 | response to purine-containing compound | Biological Process | 5 | 1 | 0.029758 |
| GO:0031056 | regulation of histone modification | Biological Process | 4 | 2 | 0.029758 |
| GO:0061008 | hepaticobiliary system development | Biological Process | 5 | 1 | 0.029758 |
| GO:0004053 | arginase activity | Molecular Function | 1 | 0 | 0.02987 |
| GO:0004066 | asparagine synthase (glutamine-hydrolyzing) activity | Molecular Function | 1 | 0 | 0.02987 |
| GO:0004392 | heme oxygenase (decyclizing) activity | Molecular Function | 1 | 0 | 0.02987 |
| GO:0004909 | interleukin-1, type I, activating receptor activity | Molecular Function | 0 | 1 | 0.02987 |
| GO:0004915 | interleukin-6 receptor activity | Molecular Function | 1 | 0 | 0.02987 |
| GO:0004948 | calcitonin receptor activity | Molecular Function | 1 | 0 | 0.02987 |
| GO:0005146 | leukemia inhibitory factor receptor binding | Molecular Function | 1 | 0 | 0.02987 |
| GO:0015196 | L-tryptophan transmembrane transporter activity | Molecular Function | 1 | 0 | 0.02987 |
| GO:0015218 | pyrimidine nucleotide transmembrane transporter activity | Molecular Function | 1 | 0 | 0.02987 |
| GO:0017098 | sulfonylurea receptor binding | Molecular Function | 0 | 1 | 0.02987 |
| GO:0019981 | interleukin-6 binding | Molecular Function | 1 | 0 | 0.02987 |
| GO:0033798 | thyroxine 5-deiodinase activity | Molecular Function | 0 | 1 | 0.02987 |
| GO:0033872 | [heparan sulfate]-glucosamine 3-sulfotransferase 3 activity | Molecular Function | 1 | 0 | 0.02987 |
| GO:0045550 | geranylgeranyl reductase activity | Molecular Function | 1 | 0 | 0.02987 |
| GO:0061928 | glutathione specific gamma-glutamylcyclotransferase activity | Molecular Function | 1 | 0 | 0.02987 |
| GO:0072570 | ADP-D-ribose binding | Molecular Function | 0 | 1 | 0.02987 |
| GO:0090624 | endoribonuclease activity, cleaving miRNA-paired mRNA | Molecular Function | 1 | 0 | 0.02987 |
| GO:0098770 | FBXO family protein binding | Molecular Function | 0 | 1 | 0.02987 |
| GO:0098808 | mRNA cap binding | Molecular Function | 1 | 0 | 0.02987 |
| GO:0099580 | ion antiporter activity involved in regulation of postsynaptic membrane potential | Molecular Function | 0 | 1 | 0.02987 |
| GO:1990715 | mRNA CDS binding | Molecular Function | 1 | 0 | 0.02987 |
| GO:0051129 | negative regulation of cellular component organization | Biological Process | 13 | 6 | 0.029909 |
| GO:0002902 | regulation of B cell apoptotic process | Biological Process | 2 | 0 | 0.030068 |
| GO:0033145 | positive regulation of intracellular steroid hormone receptor signaling pathway | Biological Process | 0 | 2 | 0.030068 |
| GO:0035729 | cellular response to hepatocyte growth factor stimulus | Biological Process | 2 | 0 | 0.030068 |
| GO:0042448 | progesterone metabolic process | Biological Process | 2 | 0 | 0.030068 |
| GO:0046629 | gamma-delta T cell activation | Biological Process | 2 | 0 | 0.030068 |
| GO:0060252 | positive regulation of glial cell proliferation | Biological Process | 2 | 0 | 0.030068 |
| GO:0071391 | cellular response to estrogen stimulus | Biological Process | 2 | 0 | 0.030068 |
| GO:0090036 | regulation of protein kinase C signaling | Biological Process | 2 | 0 | 0.030068 |
| GO:0031329 | regulation of cellular catabolic process | Biological Process | 19 | 3 | 0.030123 |
| GO:0001661 | conditioned taste aversion | Biological Process | 1 | 0 | 0.030204 |
| GO:0001712 | ectodermal cell fate commitment | Biological Process | 1 | 0 | 0.030204 |
| GO:0002268 | follicular dendritic cell differentiation | Biological Process | 1 | 0 | 0.030204 |
| GO:0002384 | hepatic immune response | Biological Process | 1 | 0 | 0.030204 |
| GO:0002884 | negative regulation of hypersensitivity | Biological Process | 1 | 0 | 0.030204 |
| GO:0003169 | coronary vein morphogenesis | Biological Process | 1 | 0 | 0.030204 |
| GO:0003195 | tricuspid valve formation | Biological Process | 1 | 0 | 0.030204 |
| GO:0006529 | asparagine biosynthetic process | Biological Process | 1 | 0 | 0.030204 |
| GO:0006788 | heme oxidation | Biological Process | 1 | 0 | 0.030204 |
| GO:0006864 | pyrimidine nucleotide transport | Biological Process | 1 | 0 | 0.030204 |
| GO:0007518 | myoblast fate determination | Biological Process | 1 | 0 | 0.030204 |
| GO:0010725 | regulation of primitive erythrocyte differentiation | Biological Process | 0 | 1 | 0.030204 |
| GO:0014806 | smooth muscle hyperplasia | Biological Process | 1 | 0 | 0.030204 |
| GO:0016107 | sesquiterpenoid catabolic process | Biological Process | 1 | 0 | 0.030204 |
| GO:0016487 | farnesol metabolic process | Biological Process | 1 | 0 | 0.030204 |
| GO:0016488 | farnesol catabolic process | Biological Process | 1 | 0 | 0.030204 |
| GO:0019060 | intracellular transport of viral protein in host cell | Biological Process | 0 | 1 | 0.030204 |
| GO:0019547 | arginine catabolic process to ornithine | Biological Process | 1 | 0 | 0.030204 |
| GO:0021526 | medial motor column neuron differentiation | Biological Process | 1 | 0 | 0.030204 |
| GO:0021593 | rhombomere morphogenesis | Biological Process | 1 | 0 | 0.030204 |
| GO:0021658 | rhombomere 3 morphogenesis | Biological Process | 1 | 0 | 0.030204 |
| GO:0021847 | ventricular zone neuroblast division | Biological Process | 0 | 1 | 0.030204 |
| GO:0030581 | symbiont intracellular protein transport in host | Biological Process | 0 | 1 | 0.030204 |
| GO:0031930 | mitochondria-nucleus signaling pathway | Biological Process | 1 | 0 | 0.030204 |
| GO:0032764 | negative regulation of mast cell cytokine production | Biological Process | 1 | 0 | 0.030204 |
| GO:0032877 | positive regulation of DNA endoreduplication | Biological Process | 1 | 0 | 0.030204 |
| GO:0033577 | protein glycosylation in endoplasmic reticulum | Biological Process | 0 | 1 | 0.030204 |
| GO:0034140 | negative regulation of toll-like receptor 3 signaling pathway | Biological Process | 1 | 0 | 0.030204 |
| GO:0034263 | positive regulation of autophagy in response to ER overload | Biological Process | 1 | 0 | 0.030204 |
| GO:0035854 | eosinophil fate commitment | Biological Process | 0 | 1 | 0.030204 |
| GO:0035936 | testosterone secretion | Biological Process | 1 | 0 | 0.030204 |
| GO:0036304 | umbilical cord morphogenesis | Biological Process | 1 | 0 | 0.030204 |
| GO:0036496 | regulation of translational initiation by eIF2 alpha dephosphorylation | Biological Process | 1 | 0 | 0.030204 |
| GO:0038018 | Wnt receptor catabolic process | Biological Process | 0 | 1 | 0.030204 |
| GO:0038190 | VEGF-activated neuropilin signaling pathway | Biological Process | 1 | 0 | 0.030204 |
| GO:0038195 | urokinase plasminogen activator signaling pathway | Biological Process | 1 | 0 | 0.030204 |
| GO:0042404 | thyroid hormone catabolic process | Biological Process | 0 | 1 | 0.030204 |
| GO:0043311 | positive regulation of eosinophil degranulation | Biological Process | 1 | 0 | 0.030204 |
| GO:0043377 | negative regulation of CD8-positive, alpha-beta T cell differentiation | Biological Process | 1 | 0 | 0.030204 |
| GO:0044533 | positive regulation of apoptotic process in other organism | Biological Process | 1 | 0 | 0.030204 |
| GO:0045210 | FasL biosynthetic process | Biological Process | 1 | 0 | 0.030204 |
| GO:0045585 | positive regulation of cytotoxic T cell differentiation | Biological Process | 1 | 0 | 0.030204 |
| GO:0045643 | regulation of eosinophil differentiation | Biological Process | 1 | 0 | 0.030204 |
| GO:0045645 | positive regulation of eosinophil differentiation | Biological Process | 1 | 0 | 0.030204 |
| GO:0045659 | negative regulation of neutrophil differentiation | Biological Process | 1 | 0 | 0.030204 |
| GO:0048320 | axial mesoderm formation | Biological Process | 1 | 0 | 0.030204 |
| GO:0048874 | host-mediated regulation of intestinal microbiota composition | Biological Process | 0 | 1 | 0.030204 |
| GO:0051542 | elastin biosynthetic process | Biological Process | 1 | 0 | 0.030204 |
| GO:0051708 | intracellular protein transport in other organism involved in symbiotic interaction | Biological Process | 0 | 1 | 0.030204 |
| GO:0052042 | positive regulation by symbiont of host programmed cell death | Biological Process | 1 | 0 | 0.030204 |
| GO:0052151 | positive regulation by symbiont of host apoptotic process | Biological Process | 1 | 0 | 0.030204 |
| GO:0052330 | positive regulation by organism of programmed cell death in other organism involved in symbiotic interaction | Biological Process | 1 | 0 | 0.030204 |
| GO:0052501 | positive regulation by organism of apoptotic process in other organism involved in symbiotic interaction | Biological Process | 1 | 0 | 0.030204 |
| GO:0060125 | negative regulation of growth hormone secretion | Biological Process | 0 | 1 | 0.030204 |
| GO:0060139 | positive regulation of apoptotic process by virus | Biological Process | 1 | 0 | 0.030204 |
| GO:0060279 | positive regulation of ovulation | Biological Process | 1 | 0 | 0.030204 |
| GO:0060400 | negative regulation of growth hormone receptor signaling pathway | Biological Process | 1 | 0 | 0.030204 |
| GO:0060529 | squamous basal epithelial stem cell differentiation involved in prostate gland acinus development | Biological Process | 0 | 1 | 0.030204 |
| GO:0060595 | fibroblast growth factor receptor signaling pathway involved in mammary gland specification | Biological Process | 0 | 1 | 0.030204 |
| GO:0060615 | mammary gland bud formation | Biological Process | 0 | 1 | 0.030204 |
| GO:0060667 | branch elongation involved in salivary gland morphogenesis | Biological Process | 0 | 1 | 0.030204 |
| GO:0060915 | mesenchymal cell differentiation involved in lung development | Biological Process | 0 | 1 | 0.030204 |
| GO:0060940 | epithelial to mesenchymal transition involved in cardiac fibroblast development | Biological Process | 0 | 1 | 0.030204 |
| GO:0061027 | umbilical cord development | Biological Process | 1 | 0 | 0.030204 |
| GO:0061300 | cerebellum vasculature development | Biological Process | 0 | 1 | 0.030204 |
| GO:0061394 | regulation of transcription from RNA polymerase II promoter in response to arsenic-containing substance | Biological Process | 1 | 0 | 0.030204 |
| GO:0061520 | Langerhans cell differentiation | Biological Process | 0 | 1 | 0.030204 |
| GO:0070563 | negative regulation of vitamin D receptor signaling pathway | Biological Process | 0 | 1 | 0.030204 |
| GO:0070950 | regulation of neutrophil mediated killing of bacterium | Biological Process | 1 | 0 | 0.030204 |
| GO:0070951 | regulation of neutrophil mediated killing of gram-negative bacterium | Biological Process | 1 | 0 | 0.030204 |
| GO:0070960 | positive regulation of neutrophil mediated cytotoxicity | Biological Process | 1 | 0 | 0.030204 |
| GO:0070961 | positive regulation of neutrophil mediated killing of symbiont cell | Biological Process | 1 | 0 | 0.030204 |
| GO:0070982 | L-asparagine metabolic process | Biological Process | 1 | 0 | 0.030204 |
| GO:0071460 | cellular response to cell-matrix adhesion | Biological Process | 0 | 1 | 0.030204 |
| GO:0071505 | response to mycophenolic acid | Biological Process | 1 | 0 | 0.030204 |
| GO:0071506 | cellular response to mycophenolic acid | Biological Process | 1 | 0 | 0.030204 |
| GO:0071881 | adenylate cyclase-inhibiting adrenergic receptor signaling pathway | Biological Process | 1 | 0 | 0.030204 |
| GO:0072185 | metanephric cap development | Biological Process | 1 | 0 | 0.030204 |
| GO:0072186 | metanephric cap morphogenesis | Biological Process | 1 | 0 | 0.030204 |
| GO:0072301 | regulation of metanephric glomerular mesangial cell proliferation | Biological Process | 1 | 0 | 0.030204 |
| GO:0090094 | metanephric cap mesenchymal cell proliferation involved in metanephros development | Biological Process | 1 | 0 | 0.030204 |
| GO:0090272 | negative regulation of fibroblast growth factor production | Biological Process | 1 | 0 | 0.030204 |
| GO:0097156 | fasciculation of motor neuron axon | Biological Process | 0 | 1 | 0.030204 |
| GO:0097534 | lymphoid lineage cell migration | Biological Process | 0 | 1 | 0.030204 |
| GO:0097535 | lymphoid lineage cell migration into thymus | Biological Process | 0 | 1 | 0.030204 |
| GO:1900135 | positive regulation of renin secretion into blood stream | Biological Process | 1 | 0 | 0.030204 |
| GO:1900923 | regulation of glycine import across plasma membrane | Biological Process | 1 | 0 | 0.030204 |
| GO:1900924 | negative regulation of glycine import across plasma membrane | Biological Process | 1 | 0 | 0.030204 |
| GO:1901551 | negative regulation of endothelial cell development | Biological Process | 1 | 0 | 0.030204 |
| GO:1902257 | negative regulation of apoptotic process involved in outflow tract morphogenesis | Biological Process | 1 | 0 | 0.030204 |
| GO:1902310 | positive regulation of peptidyl-serine dephosphorylation | Biological Process | 1 | 0 | 0.030204 |
| GO:1902336 | positive regulation of retinal ganglion cell axon guidance | Biological Process | 1 | 0 | 0.030204 |
| GO:1902359 | Notch signaling pathway involved in somitogenesis | Biological Process | 0 | 1 | 0.030204 |
| GO:1902366 | regulation of Notch signaling pathway involved in somitogenesis | Biological Process | 0 | 1 | 0.030204 |
| GO:1902367 | negative regulation of Notch signaling pathway involved in somitogenesis | Biological Process | 0 | 1 | 0.030204 |
| GO:1902568 | positive regulation of eosinophil activation | Biological Process | 1 | 0 | 0.030204 |
| GO:1903141 | negative regulation of establishment of endothelial barrier | Biological Process | 1 | 0 | 0.030204 |
| GO:1903786 | regulation of glutathione biosynthetic process | Biological Process | 1 | 0 | 0.030204 |
| GO:1903840 | response to arsenite(3-) | Biological Process | 1 | 0 | 0.030204 |
| GO:1903841 | cellular response to arsenite(3-) | Biological Process | 1 | 0 | 0.030204 |
| GO:1904245 | regulation of polynucleotide adenylyltransferase activity | Biological Process | 1 | 0 | 0.030204 |
| GO:1904556 | L-tryptophan transmembrane transport | Biological Process | 1 | 0 | 0.030204 |
| GO:1904585 | response to putrescine | Biological Process | 1 | 0 | 0.030204 |
| GO:1904586 | cellular response to putrescine | Biological Process | 1 | 0 | 0.030204 |
| GO:1905178 | regulation of cardiac muscle tissue regeneration | Biological Process | 1 | 0 | 0.030204 |
| GO:1905179 | negative regulation of cardiac muscle tissue regeneration | Biological Process | 1 | 0 | 0.030204 |
| GO:1905403 | negative regulation of activated CD8-positive, alpha-beta T cell apoptotic process | Biological Process | 1 | 0 | 0.030204 |
| GO:1990519 | pyrimidine nucleotide import into mitochondrion | Biological Process | 1 | 0 | 0.030204 |
| GO:1990646 | cellular response to prolactin | Biological Process | 1 | 0 | 0.030204 |
| GO:1990859 | cellular response to endothelin | Biological Process | 1 | 0 | 0.030204 |
| GO:2000016 | negative regulation of determination of dorsal identity | Biological Process | 1 | 0 | 0.030204 |
| GO:2000184 | positive regulation of progesterone biosynthetic process | Biological Process | 1 | 0 | 0.030204 |
| GO:2000276 | negative regulation of oxidative phosphorylation uncoupler activity | Biological Process | 1 | 0 | 0.030204 |
| GO:2000324 | positive regulation of glucocorticoid receptor signaling pathway | Biological Process | 0 | 1 | 0.030204 |
| GO:2000490 | negative regulation of hepatic stellate cell activation | Biological Process | 1 | 0 | 0.030204 |
| GO:2000843 | regulation of testosterone secretion | Biological Process | 1 | 0 | 0.030204 |
| GO:2000845 | positive regulation of testosterone secretion | Biological Process | 1 | 0 | 0.030204 |
| GO:0022602 | ovulation cycle process | Biological Process | 2 | 1 | 0.030927 |
| GO:0086009 | membrane repolarization | Biological Process | 1 | 2 | 0.030927 |
| GO:1900408 | negative regulation of cellular response to oxidative stress | Biological Process | 3 | 0 | 0.030927 |
| GO:1903202 | negative regulation of oxidative stress-induced cell death | Biological Process | 3 | 0 | 0.030927 |
| GO:2000107 | negative regulation of leukocyte apoptotic process | Biological Process | 3 | 0 | 0.030927 |
| GO:0016925 | protein sumoylation | Biological Process | 4 | 0 | 0.031134 |
| GO:0046902 | regulation of mitochondrial membrane permeability | Biological Process | 4 | 0 | 0.031134 |
| GO:1990748 | cellular detoxification | Biological Process | 5 | 0 | 0.031246 |
| GO:0002697 | regulation of immune effector process | Biological Process | 9 | 4 | 0.031357 |
| GO:0000187 | activation of MAPK activity | Biological Process | 6 | 0 | 0.031428 |
| GO:0002702 | positive regulation of production of molecular mediator of immune response | Biological Process | 4 | 1 | 0.032263 |
| GO:2000736 | regulation of stem cell differentiation | Biological Process | 4 | 1 | 0.032263 |
| GO:0051049 | regulation of transport | Biological Process | 26 | 11 | 0.032286 |
| GO:0009101 | glycoprotein biosynthetic process | Biological Process | 3 | 7 | 0.032362 |
| GO:0021536 | diencephalon development | Biological Process | 2 | 2 | 0.032416 |
| GO:0043154 | negative regulation of cysteine-type endopeptidase activity involved in apoptotic process | Biological Process | 4 | 0 | 0.032416 |
| GO:1900034 | regulation of cellular response to heat | Biological Process | 4 | 0 | 0.032416 |
| GO:0015171 | amino acid transmembrane transporter activity | Molecular Function | 4 | 0 | 0.032579 |
| GO:0042771 | intrinsic apoptotic signaling pathway in response to DNA damage by p53 class mediator | Biological Process | 3 | 0 | 0.032723 |
| GO:0043300 | regulation of leukocyte degranulation | Biological Process | 2 | 1 | 0.032723 |
| GO:0045023 | G0 to G1 transition | Biological Process | 2 | 1 | 0.032723 |
| GO:0051898 | negative regulation of protein kinase B signaling | Biological Process | 3 | 0 | 0.032723 |
| GO:0055010 | ventricular cardiac muscle tissue morphogenesis | Biological Process | 2 | 1 | 0.032723 |
| GO:0050769 | positive regulation of neurogenesis | Biological Process | 7 | 6 | 0.032736 |
| GO:0006955 | immune response | Biological Process | 37 | 10 | 0.0329 |
| GO:0031290 | retinal ganglion cell axon guidance | Biological Process | 1 | 1 | 0.033274 |
| GO:0061042 | vascular wound healing | Biological Process | 1 | 1 | 0.033274 |
| GO:0070262 | peptidyl-serine dephosphorylation | Biological Process | 2 | 0 | 0.033274 |
| GO:0070989 | oxidative demethylation | Biological Process | 1 | 1 | 0.033274 |
| GO:0097067 | cellular response to thyroid hormone stimulus | Biological Process | 2 | 0 | 0.033274 |
| GO:1900153 | positive regulation of nuclear-transcribed mRNA catabolic process, deadenylation-dependent decay | Biological Process | 2 | 0 | 0.033274 |
| GO:2000479 | regulation of cAMP-dependent protein kinase activity | Biological Process | 2 | 0 | 0.033274 |
| GO:0016538 | cyclin-dependent protein serine/threonine kinase regulator activity | Molecular Function | 2 | 1 | 0.033617 |
| GO:0031099 | regeneration | Biological Process | 6 | 1 | 0.033695 |
| GO:0061136 | regulation of proteasomal protein catabolic process | Biological Process | 5 | 2 | 0.033695 |
| GO:0003300 | cardiac muscle hypertrophy | Biological Process | 4 | 0 | 0.033728 |
| GO:0034198 | cellular response to amino acid starvation | Biological Process | 4 | 0 | 0.033728 |
| GO:0007519 | skeletal muscle tissue development | Biological Process | 6 | 0 | 0.034045 |
| GO:0046983 | protein dimerization activity | Molecular Function | 19 | 5 | 0.034226 |
| GO:0005516 | calmodulin binding | Molecular Function | 5 | 2 | 0.034308 |
| GO:0005976 | polysaccharide metabolic process | Biological Process | 2 | 3 | 0.03436 |
| GO:0072089 | stem cell proliferation | Biological Process | 3 | 2 | 0.03436 |
| GO:1901800 | positive regulation of proteasomal protein catabolic process | Biological Process | 4 | 1 | 0.03436 |
| GO:0032873 | negative regulation of stress-activated MAPK cascade | Biological Process | 3 | 0 | 0.034572 |
| GO:0046622 | positive regulation of organ growth | Biological Process | 2 | 1 | 0.034572 |
| GO:0061001 | regulation of dendritic spine morphogenesis | Biological Process | 1 | 2 | 0.034572 |
| GO:0070303 | negative regulation of stress-activated protein kinase signaling cascade | Biological Process | 3 | 0 | 0.034572 |
| GO:0008287 | protein serine/threonine phosphatase complex | Cellular Component | 2 | 1 | 0.034888 |
| GO:1903293 | phosphatase complex | Cellular Component | 2 | 1 | 0.034888 |
| GO:0005912 | adherens junction | Cellular Component | 7 | 7 | 0.035043 |
| GO:0000271 | polysaccharide biosynthetic process | Biological Process | 1 | 3 | 0.035071 |
| GO:0007422 | peripheral nervous system development | Biological Process | 4 | 0 | 0.035071 |
| GO:0042509 | regulation of tyrosine phosphorylation of STAT protein | Biological Process | 3 | 1 | 0.035071 |
| GO:0035258 | steroid hormone receptor binding | Molecular Function | 3 | 1 | 0.035212 |
| GO:0009749 | response to glucose | Biological Process | 6 | 1 | 0.035271 |
| GO:0010906 | regulation of glucose metabolic process | Biological Process | 4 | 1 | 0.035439 |
| GO:0007093 | mitotic cell cycle checkpoint | Biological Process | 6 | 0 | 0.035867 |
| GO:0021915 | neural tube development | Biological Process | 3 | 3 | 0.035867 |
| GO:0071695 | anatomical structure maturation | Biological Process | 5 | 1 | 0.035867 |
| GO:0035014 | phosphatidylinositol 3-kinase regulator activity | Molecular Function | 1 | 1 | 0.035869 |
| GO:0031175 | neuron projection development | Biological Process | 12 | 11 | 0.036253 |
| GO:0050688 | regulation of defense response to virus | Biological Process | 2 | 2 | 0.036445 |
| GO:2000106 | regulation of leukocyte apoptotic process | Biological Process | 4 | 0 | 0.036445 |
| GO:0031952 | regulation of protein autophosphorylation | Biological Process | 2 | 1 | 0.036475 |
| GO:0033628 | regulation of cell adhesion mediated by integrin | Biological Process | 2 | 1 | 0.036475 |
| GO:1902883 | negative regulation of response to oxidative stress | Biological Process | 3 | 0 | 0.036475 |
| GO:1904645 | response to amyloid-beta | Biological Process | 3 | 0 | 0.036475 |
| GO:0003012 | muscle system process | Biological Process | 7 | 5 | 0.036476 |
| GO:0033005 | positive regulation of mast cell activation | Biological Process | 1 | 1 | 0.036606 |
| GO:0035728 | response to hepatocyte growth factor | Biological Process | 2 | 0 | 0.036606 |
| GO:0036120 | cellular response to platelet-derived growth factor stimulus | Biological Process | 2 | 0 | 0.036606 |
| GO:0045655 | regulation of monocyte differentiation | Biological Process | 1 | 1 | 0.036606 |
| GO:0051900 | regulation of mitochondrial depolarization | Biological Process | 2 | 0 | 0.036606 |
| GO:0060749 | mammary gland alveolus development | Biological Process | 2 | 0 | 0.036606 |
| GO:0061081 | positive regulation of myeloid leukocyte cytokine production involved in immune response | Biological Process | 2 | 0 | 0.036606 |
| GO:0061377 | mammary gland lobule development | Biological Process | 2 | 0 | 0.036606 |
| GO:1905276 | regulation of epithelial tube formation | Biological Process | 1 | 1 | 0.036606 |
| GO:2000678 | negative regulation of transcription regulatory region DNA binding | Biological Process | 2 | 0 | 0.036606 |
| GO:0034762 | regulation of transmembrane transport | Biological Process | 11 | 4 | 0.036898 |
| GO:0009410 | response to xenobiotic stimulus | Biological Process | 8 | 1 | 0.037199 |
| GO:0048018 | receptor ligand activity | Molecular Function | 11 | 2 | 0.037455 |
| GO:0050865 | regulation of cell activation | Biological Process | 15 | 1 | 0.037522 |
| GO:0031346 | positive regulation of cell projection organization | Biological Process | 4 | 7 | 0.037593 |
| GO:0002688 | regulation of leukocyte chemotaxis | Biological Process | 5 | 0 | 0.03766 |
| GO:0007173 | epidermal growth factor receptor signaling pathway | Biological Process | 4 | 1 | 0.03766 |
| GO:0034249 | negative regulation of cellular amide metabolic process | Biological Process | 6 | 0 | 0.03775 |
| GO:0051147 | regulation of muscle cell differentiation | Biological Process | 5 | 1 | 0.03775 |
| GO:0018193 | peptidyl-amino acid modification | Biological Process | 23 | 5 | 0.037768 |
| GO:0007260 | tyrosine phosphorylation of STAT protein | Biological Process | 3 | 1 | 0.037849 |
| GO:0014897 | striated muscle hypertrophy | Biological Process | 4 | 0 | 0.037849 |
| GO:1903313 | positive regulation of mRNA metabolic process | Biological Process | 4 | 0 | 0.037849 |
| GO:1990928 | response to amino acid starvation | Biological Process | 4 | 0 | 0.037849 |
| GO:0015297 | antiporter activity | Molecular Function | 3 | 1 | 0.037965 |
| GO:0030900 | forebrain development | Biological Process | 5 | 6 | 0.038179 |
| GO:0007622 | rhythmic behavior | Biological Process | 3 | 0 | 0.03843 |
| GO:0043303 | mast cell degranulation | Biological Process | 2 | 1 | 0.03843 |
| GO:0097300 | programmed necrotic cell death | Biological Process | 3 | 0 | 0.03843 |
| GO:0071356 | cellular response to tumor necrosis factor | Biological Process | 8 | 1 | 0.038564 |
| GO:0034248 | regulation of cellular amide metabolic process | Biological Process | 11 | 1 | 0.03866 |
| GO:0002700 | regulation of production of molecular mediator of immune response | Biological Process | 5 | 1 | 0.038714 |
| GO:0019789 | SUMO transferase activity | Molecular Function | 2 | 0 | 0.039259 |
| GO:0001942 | hair follicle development | Biological Process | 2 | 2 | 0.039285 |
| GO:0032637 | interleukin-8 production | Biological Process | 4 | 0 | 0.039285 |
| GO:0032677 | regulation of interleukin-8 production | Biological Process | 4 | 0 | 0.039285 |
| GO:0006883 | cellular sodium ion homeostasis | Biological Process | 1 | 1 | 0.040061 |
| GO:0036119 | response to platelet-derived growth factor | Biological Process | 2 | 0 | 0.040061 |
| GO:0060713 | labyrinthine layer morphogenesis | Biological Process | 1 | 1 | 0.040061 |
| GO:1900151 | regulation of nuclear-transcribed mRNA catabolic process, deadenylation-dependent decay | Biological Process | 2 | 0 | 0.040061 |
| GO:1905208 | negative regulation of cardiocyte differentiation | Biological Process | 2 | 0 | 0.040061 |
| GO:1905809 | negative regulation of synapse organization | Biological Process | 0 | 2 | 0.040061 |
| GO:2000047 | regulation of cell-cell adhesion mediated by cadherin | Biological Process | 2 | 0 | 0.040061 |
| GO:0000904 | cell morphogenesis involved in differentiation | Biological Process | 9 | 9 | 0.040151 |
| GO:0070555 | response to interleukin-1 | Biological Process | 6 | 1 | 0.040289 |
| GO:0002279 | mast cell activation involved in immune response | Biological Process | 2 | 1 | 0.040438 |
| GO:0005978 | glycogen biosynthetic process | Biological Process | 1 | 2 | 0.040438 |
| GO:0009250 | glucan biosynthetic process | Biological Process | 1 | 2 | 0.040438 |
| GO:0043330 | response to exogenous dsRNA | Biological Process | 1 | 2 | 0.040438 |
| GO:0060425 | lung morphogenesis | Biological Process | 1 | 2 | 0.040438 |
| GO:0090102 | cochlea development | Biological Process | 1 | 2 | 0.040438 |
| GO:0051100 | negative regulation of binding | Biological Process | 5 | 1 | 0.04069 |
| GO:0014896 | muscle hypertrophy | Biological Process | 4 | 0 | 0.040751 |
| GO:0009746 | response to hexose | Biological Process | 6 | 1 | 0.041168 |
| GO:0009952 | anterior/posterior pattern specification | Biological Process | 6 | 1 | 0.041168 |
| GO:0016879 | ligase activity, forming carbon-nitrogen bonds | Molecular Function | 3 | 0 | 0.041342 |
| GO:0017046 | peptide hormone binding | Molecular Function | 3 | 0 | 0.041342 |
| GO:0022604 | regulation of cell morphogenesis | Biological Process | 6 | 7 | 0.041388 |
| GO:0045334 | clathrin-coated endocytic vesicle | Cellular Component | 2 | 2 | 0.041735 |
| GO:0014031 | mesenchymal cell development | Biological Process | 2 | 2 | 0.042247 |
| GO:0002448 | mast cell mediated immunity | Biological Process | 2 | 1 | 0.042497 |
| GO:0014009 | glial cell proliferation | Biological Process | 3 | 0 | 0.042497 |
| GO:0048662 | negative regulation of smooth muscle cell proliferation | Biological Process | 3 | 0 | 0.042497 |
| GO:0061028 | establishment of endothelial barrier | Biological Process | 3 | 0 | 0.042497 |
| GO:0086002 | cardiac muscle cell action potential involved in contraction | Biological Process | 1 | 2 | 0.042497 |
| GO:0061695 | transferase complex, transferring phosphorus-containing groups | Cellular Component | 5 | 3 | 0.042625 |
| GO:0002694 | regulation of leukocyte activation | Biological Process | 14 | 1 | 0.042724 |
| GO:0001959 | regulation of cytokine-mediated signaling pathway | Biological Process | 4 | 2 | 0.042729 |
| GO:0070161 | anchoring junction | Cellular Component | 7 | 7 | 0.042741 |
| GO:0005242 | inward rectifier potassium channel activity | Molecular Function | 0 | 2 | 0.042765 |
| GO:0016881 | acid-amino acid ligase activity | Molecular Function | 2 | 0 | 0.042765 |
| GO:0070412 | R-SMAD binding | Molecular Function | 2 | 0 | 0.042765 |
| GO:0060326 | cell chemotaxis | Biological Process | 8 | 1 | 0.042866 |
| GO:0006109 | regulation of carbohydrate metabolic process | Biological Process | 6 | 1 | 0.042964 |
| GO:0044446 | intracellular organelle part | Cellular Component | 117 | 45 | 0.04297 |
| GO:0031072 | heat shock protein binding | Molecular Function | 5 | 0 | 0.043098 |
| GO:0005896 | interleukin-6 receptor complex | Cellular Component | 1 | 0 | 0.043283 |
| GO:0031004 | potassium ion-transporting ATPase complex | Cellular Component | 0 | 1 | 0.043283 |
| GO:0038039 | G protein-coupled receptor heterodimeric complex | Cellular Component | 0 | 1 | 0.043283 |
| GO:0042585 | germinal vesicle | Cellular Component | 0 | 1 | 0.043283 |
| GO:0070110 | ciliary neurotrophic factor receptor complex | Cellular Component | 1 | 0 | 0.043283 |
| GO:0097598 | sperm cytoplasmic droplet | Cellular Component | 1 | 0 | 0.043283 |
| GO:1903143 | adrenomedullin receptor complex | Cellular Component | 1 | 0 | 0.043283 |
| GO:0032989 | cellular component morphogenesis | Biological Process | 13 | 12 | 0.043342 |
| GO:0003299 | muscle hypertrophy in response to stress | Biological Process | 2 | 0 | 0.043634 |
| GO:0014887 | cardiac muscle adaptation | Biological Process | 2 | 0 | 0.043634 |
| GO:0014898 | cardiac muscle hypertrophy in response to stress | Biological Process | 2 | 0 | 0.043634 |
| GO:0035929 | steroid hormone secretion | Biological Process | 1 | 1 | 0.043634 |
| GO:0071305 | cellular response to vitamin D | Biological Process | 1 | 1 | 0.043634 |
| GO:0090280 | positive regulation of calcium ion import | Biological Process | 2 | 0 | 0.043634 |
| GO:0098884 | postsynaptic neurotransmitter receptor internalization | Biological Process | 1 | 1 | 0.043634 |
| GO:0140239 | postsynaptic endocytosis | Biological Process | 1 | 1 | 0.043634 |
| GO:1901522 | positive regulation of transcription from RNA polymerase II promoter involved in cellular response to chemical stimulus | Biological Process | 2 | 0 | 0.043634 |
| GO:2000738 | positive regulation of stem cell differentiation | Biological Process | 2 | 0 | 0.043634 |
| GO:1903829 | positive regulation of cellular protein localization | Biological Process | 8 | 2 | 0.043665 |
| GO:0001656 | metanephros development | Biological Process | 3 | 1 | 0.043775 |
| GO:0022404 | molting cycle process | Biological Process | 2 | 2 | 0.043775 |
| GO:0022405 | hair cycle process | Biological Process | 2 | 2 | 0.043775 |
| GO:0042446 | hormone biosynthetic process | Biological Process | 3 | 1 | 0.043775 |
| GO:2000117 | negative regulation of cysteine-type endopeptidase activity | Biological Process | 4 | 0 | 0.043775 |
| GO:0001605 | adrenomedullin receptor activity | Molecular Function | 1 | 0 | 0.04447 |
| GO:0004800 | thyroxine 5'-deiodinase activity | Molecular Function | 0 | 1 | 0.04447 |
| GO:0004921 | interleukin-11 receptor activity | Molecular Function | 1 | 0 | 0.04447 |
| GO:0004965 | G protein-coupled GABA receptor activity | Molecular Function | 0 | 1 | 0.04447 |
| GO:0004980 | melanocyte-stimulating hormone receptor activity | Molecular Function | 1 | 0 | 0.04447 |
| GO:0005153 | interleukin-8 receptor binding | Molecular Function | 1 | 0 | 0.04447 |
| GO:0019970 | interleukin-11 binding | Molecular Function | 1 | 0 | 0.04447 |
| GO:0031208 | POZ domain binding | Molecular Function | 1 | 0 | 0.04447 |
| GO:0033142 | progesterone receptor binding | Molecular Function | 0 | 1 | 0.04447 |
| GO:0033829 | O-fucosylpeptide 3-beta-N-acetylglucosaminyltransferase activity | Molecular Function | 0 | 1 | 0.04447 |
| GO:0042015 | interleukin-20 binding | Molecular Function | 1 | 0 | 0.04447 |
| GO:0043183 | vascular endothelial growth factor receptor 1 binding | Molecular Function | 1 | 0 | 0.04447 |
| GO:0044729 | hemi-methylated DNA-binding | Molecular Function | 1 | 0 | 0.04447 |
| GO:0047718 | indanol dehydrogenase activity | Molecular Function | 1 | 0 | 0.04447 |
| GO:0061663 | NEDD8 ligase activity | Molecular Function | 1 | 0 | 0.04447 |
| GO:0070290 | N-acylphosphatidylethanolamine-specific phospholipase D activity | Molecular Function | 0 | 1 | 0.04447 |
| GO:0070699 | type II activin receptor binding | Molecular Function | 1 | 0 | 0.04447 |
| GO:0071074 | eukaryotic initiation factor eIF2 binding | Molecular Function | 1 | 0 | 0.04447 |
| GO:1905060 | calcium:cation antiporter activity involved in regulation of postsynaptic cytosolic calcium ion concentration | Molecular Function | 0 | 1 | 0.04447 |
| GO:0009069 | serine family amino acid metabolic process | Biological Process | 3 | 0 | 0.044609 |
| GO:0043392 | negative regulation of DNA binding | Biological Process | 3 | 0 | 0.044609 |
| GO:0051445 | regulation of meiotic cell cycle | Biological Process | 3 | 0 | 0.044609 |
| GO:0061014 | positive regulation of mRNA catabolic process | Biological Process | 3 | 0 | 0.044609 |
| GO:0043467 | regulation of generation of precursor metabolites and energy | Biological Process | 5 | 1 | 0.044831 |
| GO:0060538 | skeletal muscle organ development | Biological Process | 6 | 0 | 0.044831 |
| GO:0099173 | postsynapse organization | Biological Process | 3 | 3 | 0.044831 |
| GO:0000320 | re-entry into mitotic cell cycle | Biological Process | 1 | 0 | 0.044963 |
| GO:0001705 | ectoderm formation | Biological Process | 1 | 0 | 0.044963 |
| GO:0001807 | regulation of type IV hypersensitivity | Biological Process | 1 | 0 | 0.044963 |
| GO:0001907 | killing by symbiont of host cells | Biological Process | 1 | 0 | 0.044963 |
| GO:0002266 | follicular dendritic cell activation | Biological Process | 1 | 0 | 0.044963 |
| GO:0002901 | mature B cell apoptotic process | Biological Process | 1 | 0 | 0.044963 |
| GO:0002905 | regulation of mature B cell apoptotic process | Biological Process | 1 | 0 | 0.044963 |
| GO:0002906 | negative regulation of mature B cell apoptotic process | Biological Process | 1 | 0 | 0.044963 |
| GO:0003199 | endocardial cushion to mesenchymal transition involved in heart valve formation | Biological Process | 1 | 0 | 0.044963 |
| GO:0003275 | apoptotic process involved in outflow tract morphogenesis | Biological Process | 1 | 0 | 0.044963 |
| GO:0006211 | 5-methylcytosine catabolic process | Biological Process | 0 | 1 | 0.044963 |
| GO:0006714 | sesquiterpenoid metabolic process | Biological Process | 1 | 0 | 0.044963 |
| GO:0010266 | response to vitamin B1 | Biological Process | 1 | 0 | 0.044963 |
| GO:0010621 | negative regulation of transcription by transcription factor localization | Biological Process | 1 | 0 | 0.044963 |
| GO:0010983 | positive regulation of high-density lipoprotein particle clearance | Biological Process | 1 | 0 | 0.044963 |
| GO:0014040 | positive regulation of Schwann cell differentiation | Biological Process | 1 | 0 | 0.044963 |
| GO:0019857 | 5-methylcytosine metabolic process | Biological Process | 0 | 1 | 0.044963 |
| GO:0021569 | rhombomere 3 development | Biological Process | 1 | 0 | 0.044963 |
| GO:0021571 | rhombomere 5 development | Biological Process | 1 | 0 | 0.044963 |
| GO:0021769 | orbitofrontal cortex development | Biological Process | 0 | 1 | 0.044963 |
| GO:0031335 | regulation of sulfur amino acid metabolic process | Biological Process | 1 | 0 | 0.044963 |
| GO:0032641 | lymphotoxin A production | Biological Process | 1 | 0 | 0.044963 |
| GO:0032681 | regulation of lymphotoxin A production | Biological Process | 1 | 0 | 0.044963 |
| GO:0032761 | positive regulation of lymphotoxin A production | Biological Process | 1 | 0 | 0.044963 |
| GO:0032875 | regulation of DNA endoreduplication | Biological Process | 1 | 0 | 0.044963 |
| GO:0033140 | negative regulation of peptidyl-serine phosphorylation of STAT protein | Biological Process | 1 | 0 | 0.044963 |
| GO:0035279 | mRNA cleavage involved in gene silencing by miRNA | Biological Process | 1 | 0 | 0.044963 |
| GO:0035283 | central nervous system segmentation | Biological Process | 1 | 0 | 0.044963 |
| GO:0035284 | brain segmentation | Biological Process | 1 | 0 | 0.044963 |
| GO:0038086 | VEGF-activated platelet-derived growth factor receptor signaling pathway | Biological Process | 1 | 0 | 0.044963 |
| GO:0038091 | positive regulation of cell proliferation by VEGF-activated platelet derived growth factor receptor signaling pathway | Biological Process | 1 | 0 | 0.044963 |
| GO:0038154 | interleukin-11-mediated signaling pathway | Biological Process | 1 | 0 | 0.044963 |
| GO:0042942 | D-serine transport | Biological Process | 1 | 0 | 0.044963 |
| GO:0043309 | regulation of eosinophil degranulation | Biological Process | 1 | 0 | 0.044963 |
| GO:0043974 | histone H3-K27 acetylation | Biological Process | 1 | 0 | 0.044963 |
| GO:0044004 | disruption by symbiont of host cell | Biological Process | 1 | 0 | 0.044963 |
| GO:0045583 | regulation of cytotoxic T cell differentiation | Biological Process | 1 | 0 | 0.044963 |
| GO:0045658 | regulation of neutrophil differentiation | Biological Process | 1 | 0 | 0.044963 |
| GO:0046882 | negative regulation of follicle-stimulating hormone secretion | Biological Process | 1 | 0 | 0.044963 |
| GO:0048319 | axial mesoderm morphogenesis | Biological Process | 1 | 0 | 0.044963 |
| GO:0050689 | negative regulation of defense response to virus by host | Biological Process | 1 | 0 | 0.044963 |
| GO:0051728 | cell cycle switching, mitotic to meiotic cell cycle | Biological Process | 1 | 0 | 0.044963 |
| GO:0051729 | germline cell cycle switching, mitotic to meiotic cell cycle | Biological Process | 1 | 0 | 0.044963 |
| GO:0055011 | atrial cardiac muscle cell differentiation | Biological Process | 1 | 0 | 0.044963 |
| GO:0055014 | atrial cardiac muscle cell development | Biological Process | 1 | 0 | 0.044963 |
| GO:0060086 | circadian temperature homeostasis | Biological Process | 1 | 0 | 0.044963 |
| GO:0060184 | cell cycle switching | Biological Process | 1 | 0 | 0.044963 |
| GO:0060278 | regulation of ovulation | Biological Process | 1 | 0 | 0.044963 |
| GO:0060523 | prostate epithelial cord elongation | Biological Process | 0 | 1 | 0.044963 |
| GO:0060594 | mammary gland specification | Biological Process | 0 | 1 | 0.044963 |
| GO:0060648 | mammary gland bud morphogenesis | Biological Process | 0 | 1 | 0.044963 |
| GO:0060748 | tertiary branching involved in mammary gland duct morphogenesis | Biological Process | 1 | 0 | 0.044963 |
| GO:0060935 | cardiac fibroblast cell differentiation | Biological Process | 0 | 1 | 0.044963 |
| GO:0060936 | cardiac fibroblast cell development | Biological Process | 0 | 1 | 0.044963 |
| GO:0060938 | epicardium-derived cardiac fibroblast cell differentiation | Biological Process | 0 | 1 | 0.044963 |
| GO:0060939 | epicardium-derived cardiac fibroblast cell development | Biological Process | 0 | 1 | 0.044963 |
| GO:0070949 | regulation of neutrophil mediated killing of symbiont cell | Biological Process | 1 | 0 | 0.044963 |
| GO:0071464 | cellular response to hydrostatic pressure | Biological Process | 1 | 0 | 0.044963 |
| GO:0071641 | negative regulation of macrophage inflammatory protein 1 alpha production | Biological Process | 1 | 0 | 0.044963 |
| GO:0071930 | negative regulation of transcription involved in G1/S transition of mitotic cell cycle | Biological Process | 1 | 0 | 0.044963 |
| GO:0072262 | metanephric glomerular mesangial cell proliferation involved in metanephros development | Biological Process | 1 | 0 | 0.044963 |
| GO:0072717 | cellular response to actinomycin D | Biological Process | 1 | 0 | 0.044963 |
| GO:0090310 | negative regulation of methylation-dependent chromatin silencing | Biological Process | 0 | 1 | 0.044963 |
| GO:0097155 | fasciculation of sensory neuron axon | Biological Process | 0 | 1 | 0.044963 |
| GO:0098758 | response to interleukin-8 | Biological Process | 1 | 0 | 0.044963 |
| GO:0098759 | cellular response to interleukin-8 | Biological Process | 1 | 0 | 0.044963 |
| GO:0098795 | mRNA cleavage involved in gene silencing | Biological Process | 1 | 0 | 0.044963 |
| GO:1900133 | regulation of renin secretion into blood stream | Biological Process | 1 | 0 | 0.044963 |
| GO:1900623 | regulation of monocyte aggregation | Biological Process | 1 | 0 | 0.044963 |
| GO:1900625 | positive regulation of monocyte aggregation | Biological Process | 1 | 0 | 0.044963 |
| GO:1901674 | regulation of histone H3-K27 acetylation | Biological Process | 1 | 0 | 0.044963 |
| GO:1902256 | regulation of apoptotic process involved in outflow tract morphogenesis | Biological Process | 1 | 0 | 0.044963 |
| GO:1902263 | apoptotic process involved in embryonic digit morphogenesis | Biological Process | 1 | 0 | 0.044963 |
| GO:1902445 | regulation of mitochondrial membrane permeability involved in programmed necrotic cell death | Biological Process | 1 | 0 | 0.044963 |
| GO:1902811 | positive regulation of skeletal muscle fiber differentiation | Biological Process | 1 | 0 | 0.044963 |
| GO:1903984 | positive regulation of TRAIL-activated apoptotic signaling pathway | Biological Process | 1 | 0 | 0.044963 |
| GO:1904273 | L-alanine import across plasma membrane | Biological Process | 1 | 0 | 0.044963 |
| GO:1904425 | negative regulation of GTP binding | Biological Process | 1 | 0 | 0.044963 |
| GO:1904580 | regulation of intracellular mRNA localization | Biological Process | 1 | 0 | 0.044963 |
| GO:1904582 | positive regulation of intracellular mRNA localization | Biological Process | 1 | 0 | 0.044963 |
| GO:1904694 | negative regulation of vascular smooth muscle contraction | Biological Process | 0 | 1 | 0.044963 |
| GO:1905335 | regulation of aggrephagy | Biological Process | 1 | 0 | 0.044963 |
| GO:1905337 | positive regulation of aggrephagy | Biological Process | 1 | 0 | 0.044963 |
| GO:1905397 | activated CD8-positive, alpha-beta T cell apoptotic process | Biological Process | 1 | 0 | 0.044963 |
| GO:1905402 | regulation of activated CD8-positive, alpha-beta T cell apoptotic process | Biological Process | 1 | 0 | 0.044963 |
| GO:1905604 | negative regulation of maintenance of permeability of blood-brain barrier | Biological Process | 1 | 0 | 0.044963 |
| GO:1990442 | intrinsic apoptotic signaling pathway in response to nitrosative stress | Biological Process | 1 | 0 | 0.044963 |
| GO:1990839 | response to endothelin | Biological Process | 1 | 0 | 0.044963 |
| GO:2000211 | regulation of glutamate metabolic process | Biological Process | 1 | 0 | 0.044963 |
| GO:2000722 | regulation of cardiac vascular smooth muscle cell differentiation | Biological Process | 1 | 0 | 0.044963 |
| GO:2000820 | negative regulation of transcription from RNA polymerase II promoter involved in smooth muscle cell differentiation | Biological Process | 1 | 0 | 0.044963 |
| GO:0048013 | ephrin receptor signaling pathway | Biological Process | 1 | 3 | 0.045333 |
| GO:0098773 | skin epidermis development | Biological Process | 2 | 2 | 0.045333 |
| GO:1902882 | regulation of response to oxidative stress | Biological Process | 4 | 0 | 0.045333 |
| GO:1904063 | negative regulation of cation transmembrane transport | Biological Process | 4 | 0 | 0.045333 |
| GO:0045165 | cell fate commitment | Biological Process | 4 | 4 | 0.045969 |
| GO:0034284 | response to monosaccharide | Biological Process | 6 | 1 | 0.046706 |
| GO:0002886 | regulation of myeloid leukocyte mediated immunity | Biological Process | 2 | 1 | 0.046772 |
| GO:0006968 | cellular defense response | Biological Process | 3 | 0 | 0.046772 |
| GO:0009948 | anterior/posterior axis specification | Biological Process | 3 | 0 | 0.046772 |
| GO:0043536 | positive regulation of blood vessel endothelial cell migration | Biological Process | 2 | 1 | 0.046772 |
| GO:0060218 | hematopoietic stem cell differentiation | Biological Process | 3 | 1 | 0.046922 |
| GO:0050900 | leukocyte migration | Biological Process | 11 | 2 | 0.047173 |
| GO:0003283 | atrial septum development | Biological Process | 2 | 0 | 0.047321 |
| GO:0021854 | hypothalamus development | Biological Process | 2 | 0 | 0.047321 |
| GO:0035162 | embryonic hemopoiesis | Biological Process | 1 | 1 | 0.047321 |
| GO:0035994 | response to muscle stretch | Biological Process | 1 | 1 | 0.047321 |
| GO:0042026 | protein refolding | Biological Process | 2 | 0 | 0.047321 |
| GO:0045932 | negative regulation of muscle contraction | Biological Process | 1 | 1 | 0.047321 |
| GO:0051882 | mitochondrial depolarization | Biological Process | 2 | 0 | 0.047321 |
| GO:0055093 | response to hyperoxia | Biological Process | 2 | 0 | 0.047321 |
| GO:0071157 | negative regulation of cell cycle arrest | Biological Process | 2 | 0 | 0.047321 |
| GO:0090335 | regulation of brown fat cell differentiation | Biological Process | 1 | 1 | 0.047321 |
| GO:0016202 | regulation of striated muscle tissue development | Biological Process | 4 | 1 | 0.047383 |
| GO:0002696 | positive regulation of leukocyte activation | Biological Process | 10 | 1 | 0.047743 |
| GO:0000902 | cell morphogenesis | Biological Process | 12 | 11 | 0.048078 |
| GO:0010769 | regulation of cell morphogenesis involved in differentiation | Biological Process | 4 | 5 | 0.048279 |
| GO:0007420 | brain development | Biological Process | 10 | 8 | 0.0485 |
| GO:0034637 | cellular carbohydrate biosynthetic process | Biological Process | 1 | 3 | 0.048541 |
| GO:0010611 | regulation of cardiac muscle hypertrophy | Biological Process | 3 | 0 | 0.048985 |
| GO:0045744 | negative regulation of G protein-coupled receptor signaling pathway | Biological Process | 3 | 0 | 0.048985 |
| GO:0006941 | striated muscle contraction | Biological Process | 3 | 3 | 0.049227 |
| GO:0043167 | ion binding | Molecular Function | 70 | 39 | 0.049403 |
| GO:0009987 | cellular process | Biological Process | 177 | 78 | 0.049722 |

**Supplementary Table 5. The KEGG pathways enriched by 1153 aging/senescence-inducing genes.**

| **ID** | **Description** | **Class** | **Ratio** | ***P* value** |
| --- | --- | --- | --- | --- |
| ko05203 | Viral carcinogenesis | Human Diseases | 0.469 | 9.82E-48 |
| ko05166 | HTLV-I infection | Human Diseases | 0.398 | 1.50E-37 |
| ko05167 | Kaposi sarcoma-associated herpesvirus infection | Human Diseases | 0.428 | 1.14E-36 |
| ko05161 | Hepatitis B | Human Diseases | 0.455 | 6.95E-36 |
| ko04110 | Cell cycle | Cellular Processes | 0.512 | 3.43E-35 |
| ko05034 | Alcoholism | Human Diseases | 0.415 | 1.17E-34 |
| ko05215 | Prostate cancer | Human Diseases | 0.563 | 4.64E-34 |
| ko04068 | FoxO signaling pathway | Environmental Information Processing | 0.482 | 1.24E-33 |
| ko05206 | MicroRNAs in cancer | Human Diseases | 0.426 | 5.01E-33 |
| ko05200 | Pathways in cancer | Human Diseases | 0.254 | 1.09E-31 |
| ko04218 | Cellular senescence | Cellular Processes | 0.428 | 2.04E-31 |
| ko04933 | AGE-RAGE signaling pathway in diabetic complications | Human Diseases | 0.534 | 1.02E-30 |
| ko01522 | Endocrine resistance | Human Diseases | 0.51 | 2.63E-28 |
| ko05220 | Chronic myeloid leukemia | Human Diseases | 0.582 | 4.38E-28 |
| ko04010 | MAPK signaling pathway | Environmental Information Processing | 0.296 | 1.91E-25 |
| ko05212 | Pancreatic cancer | Human Diseases | 0.543 | 3.46E-25 |
| ko04722 | Neurotrophin signaling pathway | Organismal Systems | 0.437 | 4.71E-25 |
| ko05131 | Shigellosis | Human Diseases | 0.315 | 2.69E-24 |
| ko04935 | Growth hormone synthesis, secretion and action | Organismal Systems | 0.427 | 1.20E-23 |
| ko05214 | Glioma | Human Diseases | 0.519 | 1.56E-22 |
| ko05322 | Systemic lupus erythematosus | Human Diseases | 0.323 | 2.67E-22 |
| ko05223 | Non-small cell lung cancer | Human Diseases | 0.543 | 6.82E-22 |
| ko04140 | Autophagy - animal | Cellular Processes | 0.369 | 7.80E-21 |
| ko04917 | Prolactin signaling pathway | Organismal Systems | 0.5 | 9.99E-21 |
| ko05218 | Melanoma | Human Diseases | 0.5 | 3.17E-20 |
| ko04914 | Progesterone-mediated oocyte maturation | Organismal Systems | 0.418 | 3.42E-20 |
| ko05205 | Proteoglycans in cancer | Human Diseases | 0.311 | 3.92E-20 |
| ko01521 | EGFR tyrosine kinase inhibitor resistance | Human Diseases | 0.471 | 5.42E-20 |
| ko05162 | Measles | Human Diseases | 0.36 | 6.87E-20 |
| ko05219 | Bladder cancer | Human Diseases | 0.667 | 7.54E-20 |
| ko05224 | Breast cancer | Human Diseases | 0.346 | 5.46E-19 |
| ko05163 | Human cytomegalovirus infection | Human Diseases | 0.291 | 6.77E-19 |
| ko04668 | TNF signaling pathway | Environmental Information Processing | 0.395 | 1.38E-18 |
| ko04012 | ErbB signaling pathway | Environmental Information Processing | 0.443 | 2.26E-18 |
| ko05165 | Human papillomavirus infection | Human Diseases | 0.246 | 2.97E-18 |
| ko05225 | Hepatocellular carcinoma | Human Diseases | 0.326 | 3.09E-18 |
| ko05169 | Epstein-Barr virus infection | Human Diseases | 0.262 | 1.06E-17 |
| ko05418 | Fluid shear stress and atherosclerosis | Human Diseases | 0.345 | 2.87E-17 |
| ko05221 | Acute myeloid leukemia | Human Diseases | 0.472 | 3.10E-17 |
| ko05210 | Colorectal cancer | Human Diseases | 0.415 | 3.72E-17 |
| ko05135 | Yersinia infection | Human Diseases | 0.359 | 4.49E-17 |
| ko05202 | Transcriptional misregulation in cancers | Human Diseases | 0.261 | 1.13E-16 |
| ko05160 | Hepatitis C | Human Diseases | 0.319 | 1.39E-16 |
| ko05226 | Gastric cancer | Human Diseases | 0.327 | 1.78E-16 |
| ko05213 | Endometrial cancer | Human Diseases | 0.492 | 1.80E-16 |
| ko04620 | Toll-like receptor signaling pathway | Organismal Systems | 0.38 | 2.52E-16 |
| ko04115 | p53 signaling pathway | Cellular Processes | 0.458 | 2.82E-16 |
| ko04630 | Jak-STAT signaling pathway | Environmental Information Processing | 0.31 | 2.83E-16 |
| ko04211 | Longevity regulating pathway - mammal | Organismal Systems | 0.4 | 4.13E-16 |
| ko04151 | PI3K-Akt signaling pathway | Environmental Information Processing | 0.213 | 5.88E-16 |
| ko04380 | Osteoclast differentiation | Organismal Systems | 0.331 | 7.35E-15 |
| ko05235 | PD-L1 expression and PD-1 checkpoint pathway in cancer | Human Diseases | 0.374 | 1.34E-14 |
| ko04213 | Longevity regulating pathway - multiple species | Organismal Systems | 0.441 | 2.34E-14 |
| ko05230 | Central carbon metabolism in cancer | Human Diseases | 0.419 | 4.92E-14 |
| ko04625 | C-type lectin receptor signaling pathway | Organismal Systems | 0.352 | 5.64E-14 |
| ko01524 | Platinum drug resistance | Human Diseases | 0.413 | 7.67E-14 |
| ko04510 | Focal adhesion | Cellular Processes | 0.263 | 2.90E-13 |
| ko04926 | Relaxin signaling pathway | Organismal Systems | 0.311 | 3.31E-13 |
| ko04931 | Insulin resistance | Human Diseases | 0.333 | 4.03E-13 |
| ko05142 | Chagas disease (American trypanosomiasis) | Human Diseases | 0.339 | 4.37E-13 |
| ko05222 | Small cell lung cancer | Human Diseases | 0.362 | 4.88E-13 |
| ko04210 | Apoptosis | Cellular Processes | 0.303 | 4.89E-13 |
| ko05211 | Renal cell carcinoma | Human Diseases | 0.4 | 5.49E-13 |
| ko04066 | HIF-1 signaling pathway | Environmental Information Processing | 0.322 | 6.69E-13 |
| ko04550 | Signaling pathways regulating pluripotency of stem cells | Cellular Processes | 0.293 | 8.97E-13 |
| ko04657 | IL-17 signaling pathway | Organismal Systems | 0.351 | 1.39E-12 |
| ko04660 | T cell receptor signaling pathway | Organismal Systems | 0.324 | 4.26E-12 |
| ko05170 | Human immunodeficiency virus 1 infection | Human Diseases | 0.244 | 4.65E-12 |
| ko04370 | VEGF signaling pathway | Environmental Information Processing | 0.413 | 8.43E-12 |
| ko04912 | GnRH signaling pathway | Organismal Systems | 0.34 | 1.57E-11 |
| ko04919 | Thyroid hormone signaling pathway | Organismal Systems | 0.295 | 2.89E-11 |
| ko05145 | Toxoplasmosis | Human Diseases | 0.31 | 3.71E-11 |
| ko04014 | Ras signaling pathway | Environmental Information Processing | 0.228 | 3.85E-11 |
| ko04137 | Mitophagy - animal | Cellular Processes | 0.382 | 6.62E-11 |
| ko04114 | Oocyte meiosis | Cellular Processes | 0.281 | 8.15E-11 |
| ko04932 | Non-alcoholic fatty liver disease (NAFLD) | Human Diseases | 0.262 | 9.71E-11 |
| ko04659 | Th17 cell differentiation | Organismal Systems | 0.301 | 1.71E-10 |
| ko04217 | Necroptosis | Cellular Processes | 0.257 | 1.83E-10 |
| ko05133 | Pertussis | Human Diseases | 0.346 | 4.02E-10 |
| ko04930 | Type II diabetes mellitus | Human Diseases | 0.42 | 5.92E-10 |
| ko05231 | Choline metabolism in cancer | Human Diseases | 0.299 | 7.06E-10 |
| ko05216 | Thyroid cancer | Human Diseases | 0.474 | 9.30E-10 |
| ko04150 | mTOR signaling pathway | Environmental Information Processing | 0.252 | 1.05E-09 |
| ko05164 | Influenza A | Human Diseases | 0.24 | 1.92E-09 |
| ko05134 | Legionellosis | Human Diseases | 0.379 | 2.36E-09 |
| ko05132 | Salmonella infection | Human Diseases | 0.219 | 4.21E-09 |
| ko04621 | NOD-like receptor signaling pathway | Organismal Systems | 0.234 | 4.73E-09 |
| ko04152 | AMPK signaling pathway | Environmental Information Processing | 0.262 | 1.57E-08 |
| ko04910 | Insulin signaling pathway | Organismal Systems | 0.248 | 1.62E-08 |
| ko04920 | Adipocytokine signaling pathway | Organismal Systems | 0.329 | 2.51E-08 |
| ko04371 | Apelin signaling pathway | Environmental Information Processing | 0.248 | 2.58E-08 |
| ko05010 | Alzheimer disease | Human Diseases | 0.181 | 3.98E-08 |
| ko04136 | Autophagy - other eukaryotes | Cellular Processes | 0.441 | 7.92E-08 |
| ko04062 | Chemokine signaling pathway | Organismal Systems | 0.213 | 1.19E-07 |
| ko05020 | Prion diseases | Human Diseases | 0.429 | 1.27E-07 |
| ko04015 | Rap1 signaling pathway | Environmental Information Processing | 0.204 | 1.29E-07 |
| ko04071 | Sphingolipid signaling pathway | Environmental Information Processing | 0.248 | 1.66E-07 |
| ko04915 | Estrogen signaling pathway | Organismal Systems | 0.234 | 2.88E-07 |
| ko05120 | Epithelial cell signaling in Helicobacter pylori infection | Human Diseases | 0.3 | 5.79E-07 |
| ko04622 | RIG-I-like receptor signaling pathway | Organismal Systems | 0.296 | 7.52E-07 |
| ko04664 | Fc epsilon RI signaling pathway | Organismal Systems | 0.227 | 9.10E-07 |
| ko04934 | Cushing syndrome | Human Diseases | 0.209 | 3.23E-06 |
| ko04350 | TGF-beta signaling pathway | Environmental Information Processing | 0.25 | 3.54E-06 |
| ko04120 | Ubiquitin mediated proteolysis | Genetic Information Processing | 0.214 | 5.12E-06 |
| ko04658 | Th1 and Th2 cell differentiation | Organismal Systems | 0.238 | 9.11E-06 |
| ko03420 | Nucleotide excision repair | Genetic Information Processing | 0.319 | 1.03E-05 |
| ko04960 | Aldosterone-regulated sodium reabsorption | Organismal Systems | 0.333 | 1.16E-05 |
| ko04750 | Inflammatory mediator regulation of TRP channels | Organismal Systems | 0.233 | 1.30E-05 |
| ko04520 | Adherens junction | Cellular Processes | 0.264 | 1.59E-05 |
| ko05017 | Spinocerebellar ataxia | Human Diseases | 0.23 | 2.46E-05 |
| ko05152 | Tuberculosis | Human Diseases | 0.171 | 2.86E-05 |
| ko04072 | Phospholipase D signaling pathway | Environmental Information Processing | 0.17 | 9.09E-05 |
| ko04215 | Apoptosis - multiple species | Cellular Processes | 0.333 | 0.000101 |
| ko05014 | Amyotrophic lateral sclerosis (ALS) | Human Diseases | 0.259 | 0.000158 |
| ko04810 | Regulation of actin cytoskeleton | Cellular Processes | 0.167 | 0.000196 |
| ko04611 | Platelet activation | Organismal Systems | 0.192 | 0.000249 |
| ko04720 | Long-term potentiation | Organismal Systems | 0.232 | 0.000376 |
| ko04540 | Gap junction | Cellular Processes | 0.204 | 0.000619 |
| ko04928 | Parathyroid hormone synthesis, secretion and action | Organismal Systems | 0.193 | 0.000743 |
| ko03440 | Homologous recombination | Genetic Information Processing | 0.255 | 0.000801 |
| ko04929 | GnRH secretion | Organismal Systems | 0.224 | 0.000843 |
| ko03450 | Non-homologous end-joining | Genetic Information Processing | 0.429 | 0.000899 |
| ko04310 | Wnt signaling pathway | Environmental Information Processing | 0.168 | 0.00111 |
| ko04725 | Cholinergic synapse | Organismal Systems | 0.178 | 0.002118 |
| ko04390 | Hippo signaling pathway | Environmental Information Processing | 0.164 | 0.002367 |
| ko04610 | Complement and coagulation cascades | Organismal Systems | 0.187 | 0.003227 |
| ko04730 | Long-term depression | Organismal Systems | 0.21 | 0.003403 |
| ko04662 | B cell receptor signaling pathway | Organismal Systems | 0.161 | 0.003434 |
| ko05321 | Inflammatiory bowel disease (IBD) | Human Diseases | 0.2 | 0.003814 |
| ko05140 | Leishmaniasis | Human Diseases | 0.161 | 0.004171 |
| ko04216 | Ferroptosis | Cellular Processes | 0.233 | 0.004502 |
| ko05016 | Huntington disease | Human Diseases | 0.139 | 0.00456 |
| ko04670 | Leukocyte transendothelial migration | Organismal Systems | 0.169 | 0.004771 |
| ko04064 | NF-kappa B signaling pathway | Environmental Information Processing | 0.152 | 0.005742 |
| ko04060 | Cytokine-cytokine receptor interaction | Environmental Information Processing | 0.136 | 0.006131 |
| ko03460 | Fanconi anemia pathway | Genetic Information Processing | 0.204 | 0.008527 |
| ko04612 | Antigen processing and presentation | Organismal Systems | 0.182 | 0.009141 |
| ko04714 | Thermogenesis | Organismal Systems | 0.138 | 0.009164 |
| ko04921 | Oxytocin signaling pathway | Organismal Systems | 0.151 | 0.009437 |
| ko04916 | Melanogenesis | Organismal Systems | 0.167 | 0.010467 |
| ko04024 | cAMP signaling pathway | Environmental Information Processing | 0.139 | 0.01152 |
| ko04650 | Natural killer cell mediated cytotoxicity | Organismal Systems | 0.144 | 0.011761 |
| ko04623 | Cytosolic DNA-sensing pathway | Organismal Systems | 0.188 | 0.011981 |
| ko04728 | Dopaminergic synapse | Organismal Systems | 0.152 | 0.013352 |
| ko05332 | Graft-versus-host disease | Human Diseases | 0.222 | 0.014052 |
| ko04141 | Protein processing in endoplasmic reticulum | Genetic Information Processing | 0.143 | 0.017931 |
| ko00480 | Glutathione metabolism | Metabolism | 0.18 | 0.020808 |
| ko05100 | Bacterial invasion of epithelial cells | Human Diseases | 0.169 | 0.021217 |
| ko05144 | Malaria | Human Diseases | 0.182 | 0.025472 |
| ko04923 | Regulation of lipolysis in adipocyte | Organismal Systems | 0.175 | 0.025972 |
| ko04940 | Type I diabetes mellitus | Human Diseases | 0.188 | 0.027648 |
| ko05217 | Basal cell carcinoma | Human Diseases | 0.172 | 0.028876 |
| ko00620 | Pyruvate metabolism | Metabolism | 0.195 | 0.029662 |
| ko04340 | Hedgehog signaling pathway | Environmental Information Processing | 0.176 | 0.039335 |
| ko03410 | Base excision repair | Genetic Information Processing | 0.194 | 0.041396 |
| ko04013 | MAPK signaling pathway - fly | Environmental Information Processing | 0.267 | 0.041669 |
